# A phase 2 trial of the somatostatin analog pasireotide to prevent GI toxicity and acute GVHD in allogeneic hematopoietic stem cell transplant

**DOI:** 10.1371/journal.pone.0252995

**Published:** 2021-06-25

**Authors:** Sendhilnathan Ramalingam, Sharareh Siamakpour-Reihani, Lauren Bohannan, Yi Ren, Alexander Sibley, Jeff Sheng, Li Ma, Andrew B. Nixon, Jing Lyu, Daniel C. Parker, James Bain, Michael Muehlbauer, Olga Ilkayeva, Virginia Byers Kraus, Janet L. Huebner, Thomas Spitzer, Jami Brown, Jonathan U. Peled, Marcel van den Brink, Antonio Gomes, Taewoong Choi, Cristina Gasparetto, Mitchell Horwitz, Gwynn Long, Richard Lopez, David Rizzieri, Stefanie Sarantopoulos, Nelson Chao, Anthony D. Sung

**Affiliations:** 1 Division of Hematologic Malignancies and Cellular Therapy, Duke University School of Medicine, Durham, NC, United States of America; 2 Duke Cancer Institute, Durham, NC, United States of America; 3 Department of Statistical Science, Duke University, Durham, NC, United States of America; 4 Department of Medicine, Duke University, Durham, NC, United States of America; 5 Division of Geriatrics, Duke University School of Medicine, Durham, NC, United States of America; 6 Duke Molecular Physiology Institute and Department of Medicine, Duke University School of Medicine, Duke University, Durham, NC, United States of America; 7 Massachusetts General Hospital, Boston, MA, United States of America; 8 Department of Medicine, Massachusetts General Hospital, Boston, MA, United States of America; 9 Adult Bone Marrow Transplantation Service, Memorial Sloan Kettering Cancer Center and Weill Cornell Medical College, New York, NY, United States of America; University of Kentucky, UNITED STATES

## Abstract

**Background:**

Allogeneic hematopoietic stem cell transplantation (HCT) is an often curative intent treatment, however it is associated with significant gastrointestinal (GI) toxicity and treatment related mortality. Graft-versus-host disease is a significant contributor to transplant-related mortality. We performed a phase 2 trial of the somatostatin analog pasireotide to prevent gastrointestinal toxicity and GVHD after myeloablative allogeneic HCT.

**Methods:**

Patients received 0.9mg pasireotide every 12 hours from the day prior to conditioning through day +4 after HCT (or a maximum of 14 days). The primary outcomes were grade 3–4 gastrointestinal toxicity through day 30 and acute GVHD. Secondary outcomes were chronic GVHD, overall survival and relapse free survival at one year. Stool and blood samples were collected from before and after HCT for analyses of stool microbiome, local inflammatory markers, and systemic inflammatory and metabolic markers. Results were compared with matched controls.

**Results:**

Twenty-six patients received pasireotide and were compared to 52 matched contemporaneous controls using a 1–2 match. Grade 3–4 GI toxicity occurred in 21 (81%) patients who received pasireotide and 35 (67%) controls (p = 0.33). Acute GVHD occurred in 15 (58%) patients in the pasireotide group and 28 (54%) controls (p = 0.94). Chronic GVHD occurred in 16 patients in the pasireotide group (64%) versus 22 patients in the control group (42%) (p = 0.12). Overall survival at 1 year in the pasireotide group was 63% (95% CI: 47%,86%) versus 82% (95% CI: 72%, 93%) in controls (log-rank p = 0.006). Relapse-free survival rate at one year was 40% (95% CI: 25%, 65%) in the pasireotide group versus 78% (95% CI: 68%, 91%) in controls (log-rank p = 0.002). After controlling for the effect of relevant covariates, patients in the pasireotide group had attenuated post-HCT loss of microbial diversity. Analysis of systemic inflammatory markers and metabolomics demonstrated feasibility of such analyses in patients undergoing allogeneic HCT. Baseline level and pre-to-post transplant changes in several inflammatory markers (including MIP1a, MIP1b, TNFa, IL8Pro, and IL6) correlated with likelihood of survival.

**Conclusions:**

Pasireotide did not prevent gastrointestinal toxicity or acute GVHD compared to contemporaneous controls. Pasireotide was associated with numerically higher chronic GVHD and significantly decreased OS and RFS compared to contemporaneous controls. Pasireotide may provide a locally protective effect in the stool microbiome and in local inflammation as measured by stool calprotectin, stool beta-defensin, and stool diversity index.

## Introduction

Allogeneic hematopoietic stem cell transplantation (HCT) is a curative-intent treatment for malignant and non-malignant diseases; however, it can be associated with significant morbidity and treatment related mortality (TRM) approaching 30% [1–3]. Gastrointestinal (GI) toxicities are pervasive in allogeneic HCT; total body irradiation (TBI) and/or chemotherapy, commonly used in conditioning regimens to permit acceptance of the donor graft, damages the intestinal epithelium, facilitating exposure of the subendothelial matrix to digestive enzymes in the intestinal lumen, leading to tissue damage [4]. Damage to the intestinal epithelium contributes to the development of symptomatic GI toxicities as well as to the inflammatory and alloreactive milieu that causes graft versus host disease (GVHD) [5–7]. Pancreatic digestive enzymes play a particularly prominent role in epithelial damage [8]; in a canine model of TBI-induced GI injury, pancreatic duct ligation to limit pancreatic enzyme secretion attenuated mucosal injury after irradiation [9]. Inhibition of pancreatic enzyme secretion into the bowel lumen can also be achieved pharmacologically using somatostatin analogs [10, 11]. Administration of the somatostatin analog octreotide has previously been shown in a clinical study in humans to reduce severe radiation-induced GI toxicity [12].

Octreotide has a short half-life and requires frequent administration which can be impractical in many clinical situations. The somatostatin analog pasireotide has greater metabolic stability, allowing for less frequent administration [13]. In a pre-clinical mouse model, administration of pasireotide preserved mucosal surface area after TBI [14]. Furthermore, pasireotide significantly improved overall survival after TBI, an effect that was reversed by co-administration of pancreatic enzymes suggesting its efficacy is mediated by inhibiting pancreatic enzyme secretion [14].

Pasireotide has previously been shown in humans to be safe and efficacious in Cushing disease [15] and in preventing the development of postoperative pancreatic fistulas after pancreatic surgery [16]. Hypothesizing that pasireotide treatment would prevent GI toxicity and acute GI GVHD after myeloablative conditioning (MAC) in allogeneic HCT, we performed a phase 2 study of pasireotide in patients undergoing allogeneic HCT. Here we report clinical results of this study as well as results of microbiome, inflammatory testing, and metabolomics profiling of blood and stool samples.

## Materials and methods

### Patients

We enrolled patients between November of 2015 and October of 2018. To recruit patients representative of the larger allogeneic transplant populations at our centers we approached all adult patients planned to undergo myeloablative allogeneic transplants. Patients were recruited from the blood and marrow transplant clinics at the respective institutions. Conditioning chemotherapy and post-transplant monitoring/treatment took place in the inpatient stem cell transplant/intensive care units and the outpatient stem cell transplant day hospital/clinic. Eligible patients were adults age 18 years or older who planned to undergo allogeneic HCT with myeloablative conditioning from a matched sibling donor (MSD), a matched unrelated donor (MUD), or an umbilical cord blood (UCB) graft referred to the study team. Key exclusion criteria included pregnancy, clinically significant cardiac arrhythmias, risk factors for Torsades de Pointes including QT-interval prolongation (QTc >470 milliseconds) and family history of long QT syndrome, uncontrolled diabetes (Hemoglobin A1c >8), and hypocortisolism or pituitary hormone deficiency. Contemporaneous controls were selected from patients who received myeloablative allogeneic HCT from matched related, matched unrelated, and unrelated cord blood donors at our center between 2013–2019.

### Study oversight

This study (NCT02215070) was approved by the Duke University Health System and Partners Healthcare Institutional Review Boards (IRBs). Safety monitoring was supported by an independent data safety monitoring team that conducted regular monitoring visits on an annual basis after enrollment of the first 3 patients on study; findings were reviewed by the Safety Oversight Committee, also on an annual basis.

There are no products in development or marketed products associated with this research.

### Study design

This was a phase 2, single arm, multicenter trial with enrollment from 2015 to 2018. Patients received 0.9mg pasireotide by subcutaneous injection every 12 hours beginning the day prior to conditioning until the fourth day after transplant (Day +4); a maximum of 28 doses (14 days) of pasireotide were allowed. The administration schedule was chosen to provide adequate limitation of pancreatic exocrine sufficiency in the period surrounding exposure to the conditioning regimen. The 0.9mg dose has previously been studied in a phase 3 trial of pasireotide in the treatment of Cushing disease [15]. Adverse events, GI toxicity, and GVHD were monitored and patients were followed for at least one year after transplant. Given the risk of QT prolongation with pasireotide and the frequent use of QT-prolonging agents in HCT patients, an EKG was done at baseline and at 1, 1.5, and 2 hours after the first administration of pasireotide as well as when clinically indicated after that time to monitor for QT prolongation. Plasma and stool samples were planned for collection at baseline, day of transplant, day 7, and day 14 to monitor concentrations of inflammatory markers, stool microbiome diversity, and clostridia/bifidobacteria abundance. Descriptions of sample preparation, quality control, and assays used for biomarker and microbiome diversity testing are presented in the manuscript supplement.

### Controls

For clinical comparisons, contemporaneous controls were matched using the following parameters: Transplant diagnosis (acute leukemias, lymphomas, myelodysplastic syndrome [MDS]/myeloproliferative neoplasm [MPN]/other), donor (related, unrelated), graft (bone marrow, peripheral blood progenitor cell [PBPC], and cord), GVHD prophylaxis (tacrolimus/methotrexate), age, hematopoietic cell transplantation-specific comorbidity index (HCT-CI), and conditioning regimen (TBI-based, chemotherapy only). For biomarker and microbiome controls, samples were compared to available samples from myeloablative allogeneic transplants performed between 2013–2019 in our institution’s biorepository.

### Outcomes

The primary endpoints were 1) Grade 3–4 GI toxicity, defined as GI toxicity occurring from the day prior to conditioning (the day pasireotide was started) to day +30, and 2) acute GVHD. All toxicities were graded by the Common Terminology Criteria for Adverse Events (CTCAE) version 4. GI toxicity included abdominal pain, anorexia, bloating, constipation, diarrhea, dysgeusia, dyspepsia, mucositis, nausea, and vomiting. GVHD prophylaxis and antibacterial prophylaxis (with ciprofloxacin at Duke) was per institutional standards. Secondary endpoints were chronic GVHD, relapse free survival (RFS), and overall survival (OS). Exploratory endpoints were blood inflammatory biomarker concentrations, stool and blood metabolomics profile, stool microbiome diversity, and genus-level relative abundances of Clostridia and Bifidobacteria in the fecal microbiome.

### Statistical analysis

This study was planned to enroll 40 patients with matched contemporaneous controls to allow for 99% power to detect a 40% difference in GI toxicity and 80% power to detect a 25% difference in GI toxicity based on a one-sided type I error rate of 0.05. The initial statistical plan involved the use of parametric comparisons; however, due to low accrual the study was concluded early and the statistical plan was later changed to non-parametric analyses when needed, due to the lower than expected sample size.

A 1-to-2 match on controls was done. Caliper matching on propensity score [17] was used with the following covariates: Transplant diagnosis (acute leukemias, lymphomas, myelodysplastic syndrome [MDS]/myeloproliferative neoplasm [MPN]/other), donor (related, unrelated), graft (bone marrow, peripheral blood progenitor cell [PBPC], and cord), GVHD prophylaxis (tacrolimus/methotrexate), age, hematopoietic cell transplantation-specific comorbidity index (HCT-CI), and conditioning regimen (TBI-based, chemotherapy only). Hypothesis testing for comparison of proportions was done using the Chi-Square test and Fisher’s Exact Test, where appropriate; RFS and OS were compared between patients who received pasireotide and controls using the log-rank test. Competing risk analysis for cause-specific survival was tested using Gray’s test for equality of cumulative incidence functions. Analyses were conducted using R 3.5.0 (R Foundation for Statistical Computing, Vienna, Austria) and SAS version 9.4 (SAS Institute, Cary, NC). Description of the statistical analyses for microbiome and biomarker studies are presented in the Supplemental Methods section. P-values for biomarker studies were not adjusted for multiple testing due to small sample size.

## Results

### Study enrollment and study drug administration

**Fig 1:**
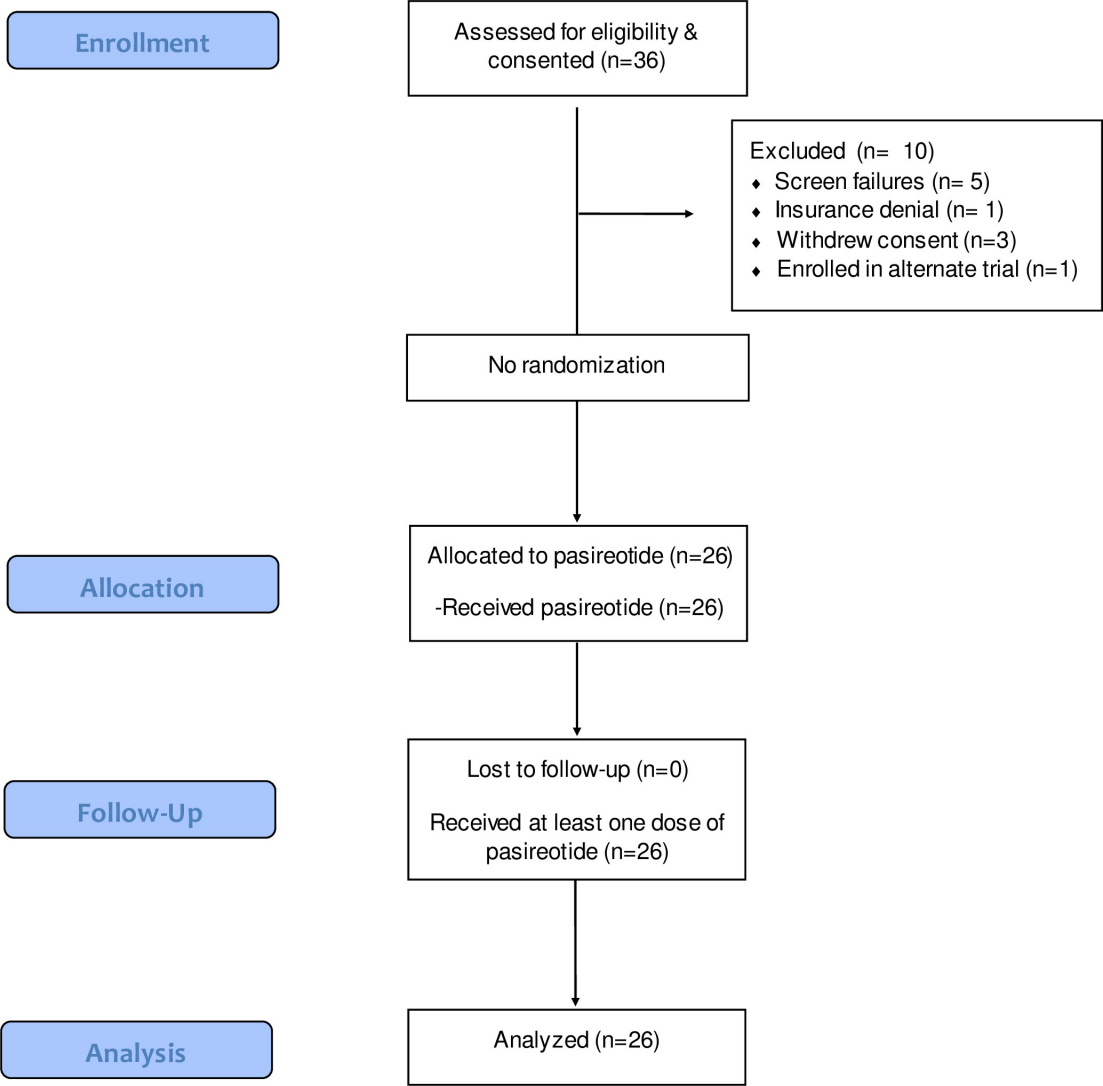
Consort diagram.

Thirty-six patients were consented for the study, and 26 patients at two centers received pasireotide (5 screen failures, 1 insurance denial, 3 withdrew consent, 1 switched to a different trial; Fig 1). Of the 26 patients who received the study drug, 21/26 (81%) were able to finish the treatment course with dosing adjustments where necessary; 23/26 (88%) were able to stay on treatment at least throughout conditioning until the day prior to transplant (ie. day -1). For those who did not finish the course of pasireotide treatment (n = 5), 1 patient withdrew consent, 1 patient developed secondary AV block, and 3 patients stopped due to nausea (one of whom was withdrawn per patient request); these 5 patients received pasireotide for 4, 4, 6, 8, and 10 days.

#### Demographics and transplant characteristics

At final analysis there were n = 26 patients in the pasireotide group and n = 52 matched controls. As shown in Table 1, matching was successful in ensuring a similar distribution of transplant diagnosis, conditioning regimen, donor, graft, and GVHD prophylaxis. Median age was 58 years in the pasireotide group and 51 years in the control group, however this was not statistically different (p = 0.96). The mean time to acute GVHD occurrence was 56.75 days in the control group and 68 days in the pasireotide group.

**Table 1:** Demographics of pasireotide and control groups.

		Pasireotide (n = 26)	Control (n = 52)	P-Value
		no. (%)	no. (%)	
**Age**	Median (IQR)	58 (41–61)	51 (36–52)	0.96
Days to engraftment	Median (IQR)	23 (18–34)	22 (18–32)	0.57
**Gender**	Male	17 (68%)	29 (56%)	0.25
**Race**	White	22 (85%)	43 (83%)	0.55
	Black	2 (8%)	7 (14%)	
	Other	2 (8%)	2 (4%)	
**Transplant Diagnosis**	Acute Leukemia	14 (54%)	31 (60%)	0.83
	MDS/MPNs/Other	9 (35%)	16 (31%)	
	Lymphoma	3 (12%)	5 (10%)	
**Conditioning**	Chemotherapy	17 (65%)	30 (58%)	0.63
	TBI	9 (35%)	22 (42%)	
**Donor**	Unrelated	17 (65%)	36 (69%)	0.73
	Related	9 (35%)	16 (31%)	
**Graft**	PBPC	16 (62%)	33 (64%)	0.65
	BM	7 (27%)	10 (19%)	
	Cord	3 (12%)	9 (17%)	
**GVHD Prophylaxis**	Tac+MTX	22 (85%)	42 (81%)	0.76
	Other	4 (15%)	10 (19%)	
**HCT-CI**	Median (IQR)	3 (2–3)	3 (2–3.5)	0.85

MDS-Myelodysplastic Syndrome, MPN-Myeloproliferative Neoplasm, TBI-Total Body Irradiation, BM-Bone Marrow, PBPC-Peripheral blood progenitor cell, Tac-Tacrolimus, MTX-Methotrexate.

### Primary outcomes—GI toxicity and acute GVHD—did not vary by treatment group

GI toxicities occurred in 100% of the pasireotide group and 100% of controls. There was no evidence of a statistical difference in grade 3–4 GI toxicity, occurring in 21 (81%) patients who received pasireotide and 35 (67%) controls (p = 0.33). There was also no evidence of a statistical difference in the development of acute GVHD, occurring in 15 (58%) patients in the pasireotide group and 28 (54%) controls (p = 0.94). Grade 2–4 acute GVHD also did not show evidence of variation between the groups, occurring in 10 patients in the pasireotide group (38%) versus 15 patients in the control group (29%) (p = 0.55, S1 Fig in S1 File). Specific sites of acute GVHD are shown in S1 Table in S2 File, and included numerically higher rates of upper GI (35% vs 21%), lower GI (23% versus 12%), and skin GVHD (50% vs 29%) in the pasireotide group and a numerically higher rate of liver GVHD in controls (6% vs 0%).

In the subgroup of patients who received TBI conditioning, there was no evidence of a statistical difference in grade 3–4 GI Toxicity, occurring in 8 (89%) patients who received pasireotide and 20 (91%) controls (p = 0.66, S2A Fig in S1 File). There was also no evidence of a statistical difference in the development of clinically significant (Grade 2–4) acute GVHD in those who received TBI conditioning, occurring in 3 (33%) patients in the pasireotide group and 8 (36%) controls (p = 0.99, S2B Fig in S1 File).

### Long term outcomes (chronic GVHD, RFS, OS)

There was no evidence of a statistical difference in chronic GVHD, which occurred in 16 patients in the pasireotide group (64%) versus 22 patients in the control group (42%) (p = 0.12). Incidence of chronic GVHD at specific sites is shown in S2 Table in S2 File and included numerically higher rates in the pasireotide group in skin (52% vs 38%), eye (24% vs 19%), mouth (32% vs 23%), GI (28% vs 6%) and liver (32% vs 10%) GVHD and numerically higher rates among controls in lung (12% vs 4%), joint/fascia (12% vs 8%), and genital (6% vs 4%) GVHD. Severe chronic GVHD (by NIH Consensus Criteria) occurred in 8 patients in the pasireotide group (32%) and 11 (21%) controls (S2 Table in S2 File).

One-year survival (Table 2) in the pasireotide group was 63% (95% CI: 47%,86%) versus 82% (95% CI: 72%, 93%) in controls (log-rank p = 0.006). RFS (Table 2) rate at one year was 40% (95% CI: 25%, 65%) in the pasireotide group versus 78% (95% CI: 68%, 91%) among controls (log-rank p = 0.002). Kaplan-Meier survival curves are displayed in S3A and S3B Fig in S1 File. Causes of death are summarized in Table 3; the majority of deaths were related to relapse in both groups (n = 8/57% of deaths among patients in the pasireotide group, n = 6/55% of deaths among controls).

**Table 2:** 1-year survival proportion with confidence intervals.

	Pasireotide		Control	
	Proportion	95% CI	Proportion	95% CI
Overall Survival	0.63	(0.47,0.86)	0.82	(0.72,0.93)	
Relapse-free Survival	0.40	(0.25,0.65)	0.78	(0.68,0.91)	

CI—Confidence interval.

**Table 3:** Summary of cause of death. NRM- non-relapse mortality.

	Pasireotide	Control	p-value
	no. (%)	no. (%)	
	n = 26	n = 52	
**Relapse**	8 (30.8%)	6 (11.5%)	0.056
**GVHD**	4 (15.4%)	1 (1.9%)	0.02
**Other NRM**	2 (7.7%)	4 (7.7%)	0.98

### Toxicities

Toxicities that occurred in over 40% of patients are shown in Table 4, with non-GI toxicities including fatigue, rash, febrile neutropenia, hypertension, hyperglycemia, and headache. A detailed list of all toxicities documented is shown in S3 Table in S2 File.

**Table 4:** Toxicities that occurred in >40 percent of patients; percentages adjusted for missing values.

	Pasireotide				Control			
	All		G3/4		All		G3/4	
	n	%	n	%	N	%	N	%
Nausea	25	100%	5	20%	43	83%	0	0%
Diarrhea	24	96%	6	24%	45	87%	5	10%
Mucositis	24	96%	14	56%	49	94%	32	62%
Anorexia	21	84%	5	20%	35	67%	8	15%
Fatigue	20	80%	0	0%	35	67%	0	0%
Vomiting	20	80%	2	8%	25	48%	0	0%
Rash	19	76%	0	0%	25	48%	0	0%
Febrile Neutropenia	17	68%	17	68%	33	63%	33	63%
Abdominal Pain	14	56%	0	0%	14	27%	0	0%
Hypertension	14	56%	9	36%	19	37%	13	25%
Hyperglycemia	12	48%	11	44%	15	29%	11	21%
Headache	11	44%	0	0%	25	48%	1	2%

### Effect of pasireotide on the microbiome and stool inflammatory markers

#### Microbiome diversity

**Fig 2:**
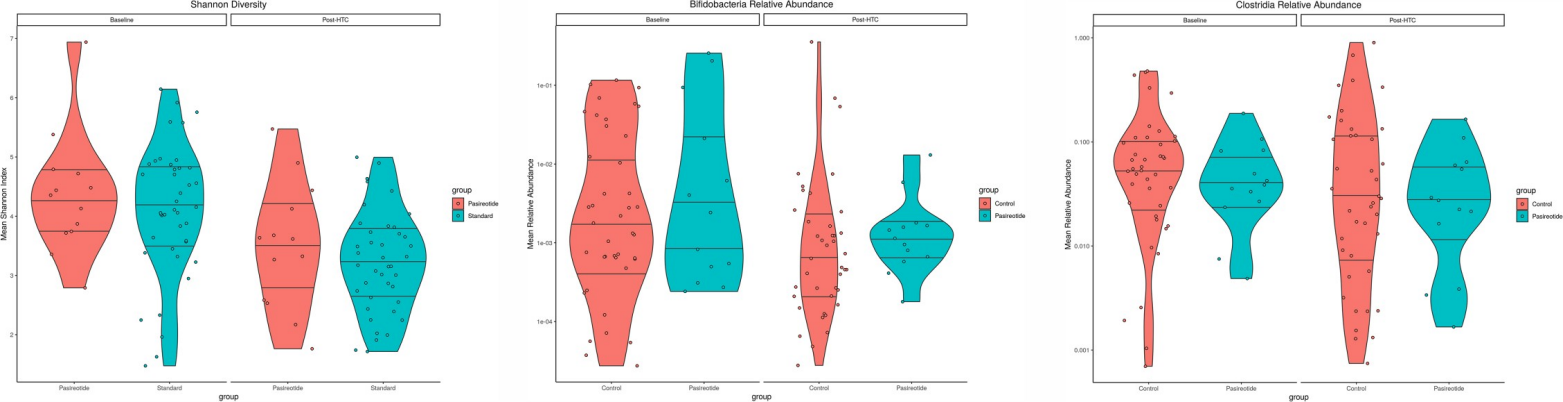
Microbiome results. (A) Mean Shannon Diversity at baseline and post-transplant. (B) Mean relative abundance of bifidobacteria in pasireotide and control groups at baseline and post-HCT. (C) Mean relative abundance of clostridia in pasireotide and control groups at baseline and post-HCT.

To examine the relationship between blocking pancreatic enzymes and the microbiome, we compared fecal microbiome profiles from the 13 patients in the pasireotide group and 41 controls in both pre- and post-transplant stool samples (Fig 2A). Mean baseline Shannon Index was similar between the two groups, with a median of 4.4 for the pasireotide group and 4.1 for controls. Using a multivariable regression model to control for the effects of baseline diversity, TBI, and when baseline samples were collected, there was initially no evidence of a statistical difference in mean post-transplant diversity between the two groups (p = 0.24), nor of an interaction between TBI and pasireotide treatment (p = 0.20). However, when the model was expanded to account for exposure to antibiotics active against anaerobic organisms, exposure to pasireotide was associated with higher mean post-transplant stool microbiome diversity than was observed in controls (S4 Table in S2 File, p = 0.0230). This difference was also present in an alternative model incorporating febrile neutropenia (FN) as a variable (S5 Table in S2 File, p = 0.0133).

#### Relative abundance of clostridia and bifidobacteria

The relative abundance (RA) of specific genera was also computed. In the pasireotide group, the RA of bifidobacteria fell from a median of 0.00242 at baseline to 0.00114 post-transplant. In the control group, median baseline RA was 0.00178 compared to 0.00046 post-transplant (Fig 2B). For RA of clostridia, post-transplant abundance fell to a median of 0.02739 from 0.03864 at baseline in the pasireotide group (Fig 2C). In the control group, the median post-transplant RA was 0.02863 compared to 0.05403 at baseline. For the selected clostridia clusters, only IV and XIVa were present. The median post-transplant RA was 0.02168 in comparison with 0.02475 at baseline in the pasireotide group. In the control group, the median post-transplant RA decreased to 0.00976 from 0.03079 at baseline. In the statistical analysis of RA of clostridia, no evidence was found for a difference in mean post-transplant RA for the two treatment groups, either across all clusters (p = 0.60) or when limited to clostridia IV and XIVa (p = 0.43).

#### Stool calprotectin and beta-defensin

To examine the effects of blocking pancreatic enzymes on local markers of inflammation, stool calprotectin and beta defensin levels were measured before and after HCT. In the pasireotide group, we did not detect a statistical difference in stool concentrations of calprotectin (median pre-HCT 11.1ng/g, post-HCT 8.6 ng/g; p = 0.9) or beta-defensin (median pre-HCT 24.0 ug/g, median post-HCT 15.4 ug/g; p = 0.7) between pre and post-transplant time points (Table 5). These data were not available for controls for purposes of comparison.

**Table 5:** Fecal biomarkers of inflammation.

Marker	Estimated mean diff	P-value
beta-defensin 2 (ng/g)	0.22578	0.718
Calprotectin, ug/g (comb)	-0.09848	0.897

### Inflammatory and metabolic changes after HCT in pasireotide and control groups

#### Blood inflammatory biomarker results

To examine the effect of pasireotide and HCT on systemic markers of inflammation, blood-based biomarkers for both inflammatory panels (54 Plex panel and the Duke Pepper Panel as described in the S5 File) were analyzed in n = 20 patients who received pasireotide. Due to the small sample size and large number of biomarkers analyzed, it was decided during the study design that p-values without multiplicity adjustment would be reported. We observed post-transplant increases in IL-15 (Interleukin 15), MCP1 (C-C motif chemokine ligand 2), CRP (C-reactive protein), SAA (serum amyloid A), IL-7 (interleukin 7), MCP4 (C-C motif chemokine ligand 13), TSLP (thymic stromal lymphopoietin), VEGF (vascular endothelial growth factor)-D, PIGF (phosphatidylinositol glycan anchor biosynthesis class F), IL-6 (interleukin 6), IL-8.Pro (interleukin 8), IL27 (interleukin 27), Eotaxin, TNFRI (TNF receptor superfamily member 1A), D-Dimer, IL-1RL/ST2 (suppression of tumorigenicity 2), MMP3 (matrix metallopeptidase 3), and TNFR2 (TNF receptor superfamily member 1B) and decreases in MDC (ADAM metallopeptidase domain 11), RANTES (C-C motif chemokine ligand 5), IL-12/IL-23p40 (interleukin 12B), Tie2 (TEK receptor tyrosine kinase), IL-16 (interleukin 16), bFGF (fibroblast growth factor 2), VEGF-C, IL-12/p70, TARC (C-C motif chemokine ligand 17), IL-17D (interleukin 17D), IL-6Ra (interleukin 6 receptor), and Paraoxonase (S6A and S6B Table in S2 File). Notably, we did not find evidence of a change in REG3A (regenerating family member 3 alpha) from pre-HCT to post-HCT in the pasireotide group (p = 0.22). Baseline levels of the following markers were associated with clinical outcomes: MIP-1b (C-C motif chemokine ligand 4), TNF-a (tumor necrosis factor alpha), IL-8.Pro, MIP-1a, IL-13 (interleukin 13), IL-6, IL-12p70, TSLP, and IL-22 (interleukin 22) with worsened OS, D-dimer with improved OS (HR 0.27, p = 0.03)), REG3A with increased risk of chronic GVHD (OR 8.98, P = 0.045), and ST2 with decreased risk of chronic GVHD (OR 0.15, p = 0.039). Furthermore, changes from baseline to post-HCT day 14 in MIP-1b (HR 8.71, p = 0.02), TNF-a (HR 2.64, p = 0.02), IL-8.Pro (HR 6, p = 0.047), and MIP-1a (HR 1.16, p = 0.01), and IL-6 (HR 1.38, p = 0.025) were associated with decreased OS. A full list of all markers analyzed and results of clinical correlation testing is show in the supplement.

#### Blood based metabolomics results

Three domains of the metabolome were analyzed in patients in the pasireotide group: (1) Conventional metabolic markers including 3-Hydroxybutyrate, lactate, non-esterified fatty acids, triglycerides, glycerol; (2) Amino acids (AA); and (3) Acylcarnitines (AC). No evidence of longitudinal changes were noted in the pasireotide group in conventional metabolic markers between baseline and post-HCT time points. However, longitudinal changes were noted between baseline and post-HCT in both the AA profile (including in the amino acid citrulline) and AC profile (S11 Table in S2 File). For the AA profile, decreases were noted in citrulline, proline, alanine, glycine, asparagine, and glutamine and increases were noted in phenylalanine and valine. In the AC profile, the pasireotide group had decreases in C8:1, C18, C8:1-OH/C6:1-DC, C8:1-DC, C16, C18:2, C5, C18:1, C5:1, C14, C12, C4-DC/Ci4-DC, C14:1-OH, C20:4, C10:3, C20, C10, C10:2, C20-OH/C18-DC, C3, C8, and C22. Furthermore, levels of C20-OH-C18-DC and C22 were associated with OS (HR 12.4, p = 0.027, and HR 6.73, p = 0.031, respectively); baseline levels of C18:2 and C18:1 were associated with cGVHD (OR 44.15 p = 0.019 and OR 19.7 p = 0.019, respectively). The longitudinal change in C18:2 was associated with decreased risk of cGVHD (OR 0.02, p = 0.037). No other associations of metabolomics baseline levels or longitudinal changes were observed to be associated with survival, GVHD, or TRM.

## Discussion

To our knowledge, this is the first prospective trial of the somatostatin analog pasireotide in allogeneic HCT. In this phase 2 study, we compared 26 patients treated with pasireotide from the day prior to conditioning to day +4 (or a maximum of 14 days) to 52 matched controls. We found no evidence of an effect of pasireotide on the development of grade 3/4 GI toxicity or in the development of acute GVHD after myeloablative allogeneic HCT. We also did not detect a benefit in terms of chronic GVHD, RFS, or OS. We did find that patients in the pasireotide group had significantly decreased OS and RFS at one year. This includes higher rates of both relapse-related deaths and GVHD-related deaths in the pasireotide group. Though it is possible this difference is a result of uncontrolled confounders, a negative effect of pasireotide cannot be ruled out.

These negative findings stand in contrast to preclinical studies that found pasireotide to be associated with improved survival after TBI [14]. Furthermore, clinical studies have found benefit to using another somatostatin analog, octreotide, for GI toxicity in HCT: (1) Octreotide reduced refractory diarrhea after HCT with a response rate of 82% in one study [18], (2) it is associated with improvement in diarrhea attributed to gastrointestinal GVHD [19], and (3) it has been shown to improve upper GI symptoms associated with HCT as well [20]. Outside of HCT, octreotide was also found to improve radiation-related GI toxicity in patients undergoing pelvic irradiation [12]. Furthermore, in a head to head comparison with octreotide in the treatment of acromegaly pasireotide was not associated with worsened GI toxicities [21]. It is not clear whether pharmacologic differences between pasireotide and octreotide could be contributing to this difference: Pasireotide has a cyclohexapeptide structure with greater metabolic stability than octreotide related to presence of a cysteine-cysteine bridge leading to improved stability of the amide bond in the cyclic ring [22]. Octreotide has specific affinity for somatostatin receptor 2 while pasireotide binds to somatostatin receptor 2 with lower affinity, but with somatostatin receptors 1, 3, and 5 with 5–40 fold higher affinity. It is rapidly absorbed, reaching peak concentration within 1 hour with terminal half life of 11.8 hours versus 2.3 hours for octreotide [13].

While our clinical endpoints do not suggest a benefit of pasireotide, we did note a positive effect of pasireotide on intestinal homeostasis both in terms of the microbiome and local markers of inflammation. Decreased microbiome diversity has previously been associated with the development of GVHD [23, 24] and worsened overall survival [25]. We found that after controlling for the effect of covariates including anaerobic antibiotics, patients in the pasireotide group tended to have greater post-transplant microbiome diversity as measured by the Shannon Index (S4 Table in S2 File) compared to controls, suggesting pasireotide may have an effect on preserving or improving stool microbiome diversity after allogeneic HCT. The pasireotide group was also observed to have numerically attenuated decreases in the relative abundance of bifidobacteria and clostridia clusters, which are associated with positive regulatory T-cell (T-reg) differentiation [26, 27] and decreased GVHD [28]. Although this difference was not statistically significant in a multivariable regression model, the analyses may have been limited by our small sample size. Furthermore, in the pasireotide group, measured markers of intestinal inflammation in the stool that we would expect to increase with myeloablative conditioning did not change: neither stool beta defensin (which is a well-characterized marker of inflammation in IBD [29–31]) nor calprotectin (which has been described as a potential marker for GI GVHD [32–35]) showed evidence of change from baseline to post-HCT. Taken together these microbiome and inflammatory marker results suggest a positive local effect of pasireotide in the gut and overall benefits may be seen with other somatostatin analogues.

Prior studies have suggested prognostic relevance of metabolomics in HCT [36, 37], and we found similar evidence in our study. Acylcarnitines are important substrates for fatty acid oxidation in alloreactive T-cell proliferation during GVHD [38, 39] and variation in baseline profiles have been associated with GVHD [36]. Indeed we found that compared to pre-HCT baseline blood samples, post-HCT blood samples were associated with decreases in 22 acylcarnitines. Elevated levels of C20-OH/C18-DC and C22 at baseline (pre-HCT) were also associated with worsened OS and elevated levels of C18:1 and C18:2 were associated with increased cGVHD. Furthermore we found that baseline levels of several inflammatory markers previously linked to worsened HCT outcomes (including TNF-a [40], IL12/p70 [41], IL-6 [42], and IL22 [43]) were associated with decreased survival. Pre-HCT levels of REG3A in the pasireotide group (implicated as a marker for GI GVHD [44, 45]) were also associated with the development of chronic GVHD. When comparing pre-HCT to post-HCT (D14) samples, changes in MIP-1a, MIP-1b, TNF-a, IL-8.Pro, and IL-6, were associated with increased risk of death in our sample (HR 1.16, 8.71, 2.64, 6, and 1.38 respectively, with p<0.05). Furthermore in the pasireotide group, pre-to-post-HCT changes were noted in markers previously associated with transplant outcomes including GVHD (with increases in CRP [46, 47], SAA [48], IL7 [49], TSLP [50], PlGF [51], IL6 [42], IL27 [52], TNFR1 [53], IL1RL/ST2 [54], MMP3 [55], TNFRII [56] and decreases in bFGF [57], IL12/p70 [41], IL6Ra [58], and Paraoxonase [48]), non-relapse mortality (D-Dimer [59]) and OS (Il1RL/ST2) [60]. Differences in sample size and collection media restricted our ability to compare inflammatory and metabolomics profiling between pasireotide and controls, however the associations and references listed above in the pasireotide group are consistent with prior reports of the prognostic relevance of inflammatory and metabolic markers in HCT. Lastly, in our sample, baseline ST2 (HR 0.15, p = 0.039) was associated with improved risk of chronic GVHD; however, prior reports have found that increased ST2 levels after HCT are associated with increased risk of refractory chronic GVHD/death [54, 60]. While this will require further evaluation, the discrepancy may be related to the time point of measurement (pre-HCT in our study versus post-HCT in prior literature).

There are several limitations to our study that may have decreased our ability to detect an effect of pasireotide as well as our interpretation of results. First, while we found evidence of a protective local effect of pasireotide on the microbiome and gut inflammation, we did not detect clinical differences in GI toxicity and acute GVHD. This may suggest a biochemically apparent benefit that was not clinically apparent, perhaps because of baseline differences unaccounted for in our matching, small sample size, or an overwhelming insult from myeloablative conditioning that overcame any benefit of pasireotide. Second, unlike pre-clinical models [14] and prior clinical studies of pasireotide in acromegaly [13] and Cushing disease [21], patients undergoing HCT are heavily pre-treated with prior courses of chemotherapy and their gut may be more prone to gastrointestinal toxicity from pasireotide, increasing the rate of GI toxicity in the pasireotide group. Additionally, our inflammatory/metabolomic analysis is limited in definitive comparisons as this was a single arm study and only matched contemporaneous controls were available. Finally, given the number of biomarkers and outcomes analyzed, and the small study population, we must consider the potential for false discoveries in our results due to multiple testing.

In conclusion, administration of the somatostatin analog pasireotide was not observed to ameliorate GI toxicity or acute GVHD after allogeneic HCT in this phase II trial. There may be an effect of pasireotide on attenuating inflammatory changes in the gut and the preservation of intestinal biodiversity. We also noted several prognostically relevant changes in the inflammatory/metabolomic profile in markers linked to HCT outcomes in the pasireotide group. Though these observations require validation in an independent study population, they present intriguing avenues for future investigation. Patients in the pasireotide group had decreased survival outcomes; though this may be a reflection of baseline differences biasing against the pasireotide group, a negative effect of pasireotide is possible and cannot be ruled out. Future exploration of the use of somatostatin analogs in HCT may include the use of octreotide, which has previously shown clinical efficacy in mitigating HCT-related gastrointestinal toxicities.

## Supporting information

S1 File S1 Fig Grade 2–4 Acute GVHD; S2 Fig Subgroup analysis among patients who received TBI conditioning. (A) Pasireotide did not reduce the rate of grade 3–4 GI toxicity, (B) Pasireotide did not reduce the rate of Grade 2–4 Acute GVHD; S3A Fig Kaplan-Meier curves for overall survival; S3B Fig Kaplan-Meier curves for relapse free survival.
(PPTX)

S2 File S1 Table Acute GVHD by site; S2 Table. Chronic GVHD by site. *n = 25 is adjustment for one missing value; S3 Table. All toxicities with CTCAE frequency by treatment group. Percentages adjusted for missing values; S4 Table. Model predicting Shannon diversity incorporating anaerobic antibiotic coverage; S5 Table. Model predicting Shannon diversity incorporating febrile neutropenia; S6A Table. Pasireotide group inflammatory changes baseline to day 14 median/range followed by significance testing (54 plex panel); S6B Table. Pasireotide group inflammatory changes baseline to day 14 median/range followed by significance testing (Pepper panel); S7A Table. Pasireotide group inflammatory overall survival correlations for baseline and ratio of change from baseline to day 14 (54 plex panel); S7B Table. Pasireotide group inflammatory overall survival correlations for baseline and ratio of change from baseline to day 14 (Pepper panel); S8A Table. Pasireotide group inflammatory aGVHD correlations (54-plex panel); S8B Table. Pasireotide group inflammatory aGVHD correlations (Pepper panel); S9A Table. Pasireotide group inflammatory cGVHD correlations (54-plex panel); S9B Table. Pasireotide group inflammatory cGVHD correlations (Pepper panel); S10A Table. Pasireotide group inflammatory TRM correlations (54-plex panel); S10B Table: Pasireotide group inflammatory TRM correlations (Pepper panel); S11 Table. Pasireotide group metabolomics change from baseline to D14, median/range followed by significance testing. Conventional-C, Amino Acid-AA, Acylcarnitines-AC; S12 Table. Pasireotide group metabolomics correlation to overall survival Conventional-C, Amino Acid-AA, Acylcarnitines-AC; S13 Table. Pasireotide group metabolomics correlation to aGVHD Conventional-C, Amino Acid-AA, Acylcarnitines-AC; S14 Table. Pasireotide group metabolomics correlation to cGVHD Conventional-C, Amino Acid-AA, Acylcarnitines-AC; S15 Table. Pasireotide group metabolomics correlation to TRM Conventional-C, Amino Acid-AA, Acylcarnitines-AC.
(DOCX)

S3 File (PDF)

S4 File Study protocol. (PDF)

S5 File Description of methods for blood-based biomarker analysis, blood and stool metabolomics, stool microbiome, statistical analysis for biomarker studies, and statistical analysis for microbiome studies. (DOCX)
