# Supplementary material for: A phase 2 trial of the somatostatin analog pasireotide to prevent GI toxicity and acute GVHD in allogeneic hematopoietic stem cell transplant
Source: PLoS One. 2021 Jun 25;16(6):e0252995. doi: 10.1371/journal.pone.0252995 (PMC8232534; doi:10.1371/journal.pone.0252995)
Supplement: S1 File — (A) Pasireotide did not reduce the rate of grade 3–4 GI toxicity, (B) Pasireotide did not reduce the rate of Grade 2–4 Acute GVHD; S3A Fig Kaplan-Meier curves for overall survival; S3B Fig Kaplan-Meier curves for relapse free survival. (PPTX) [file pone.0252995.s001.pptx]

## Slide 1
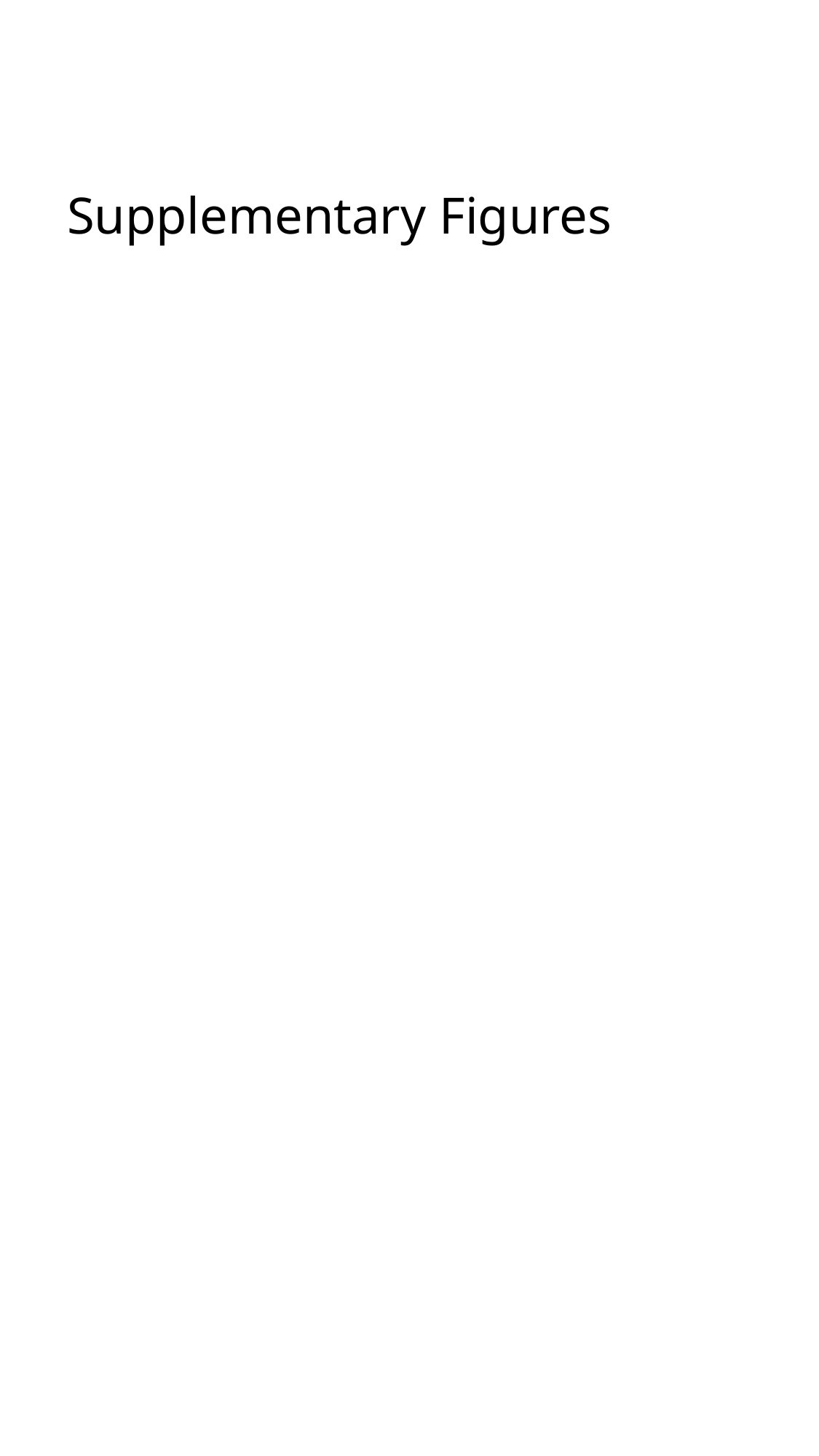

# Supplementary Figures

## Slide 2
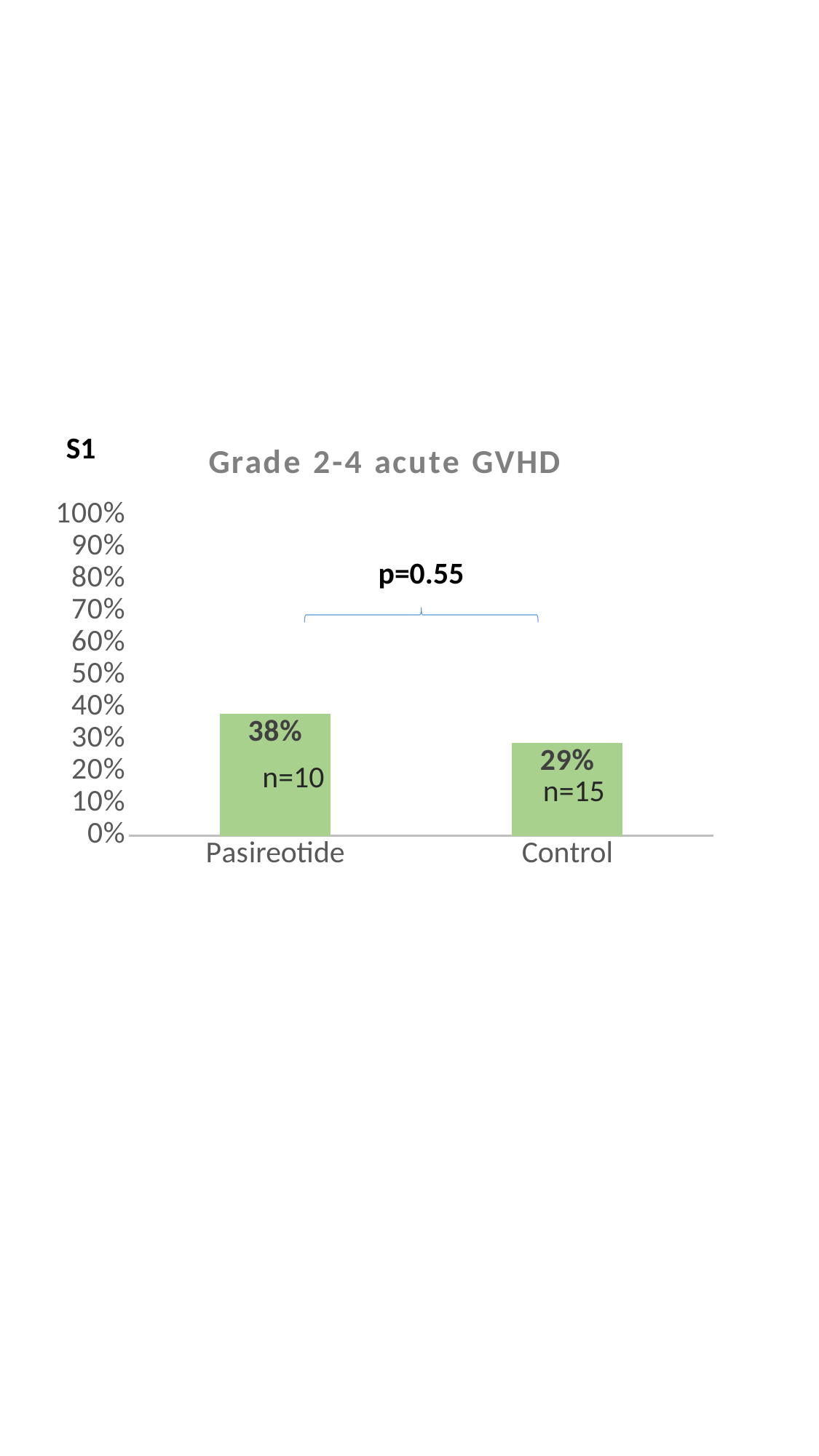

### Chart: Grade 2-4 acute GVHD
| Category | Any GI toxicity |
|---|---|
| Pasireotide | 0.38 |
| Control | 0.29 |
p=0.55
n=10
n=15
S1

## Slide 3
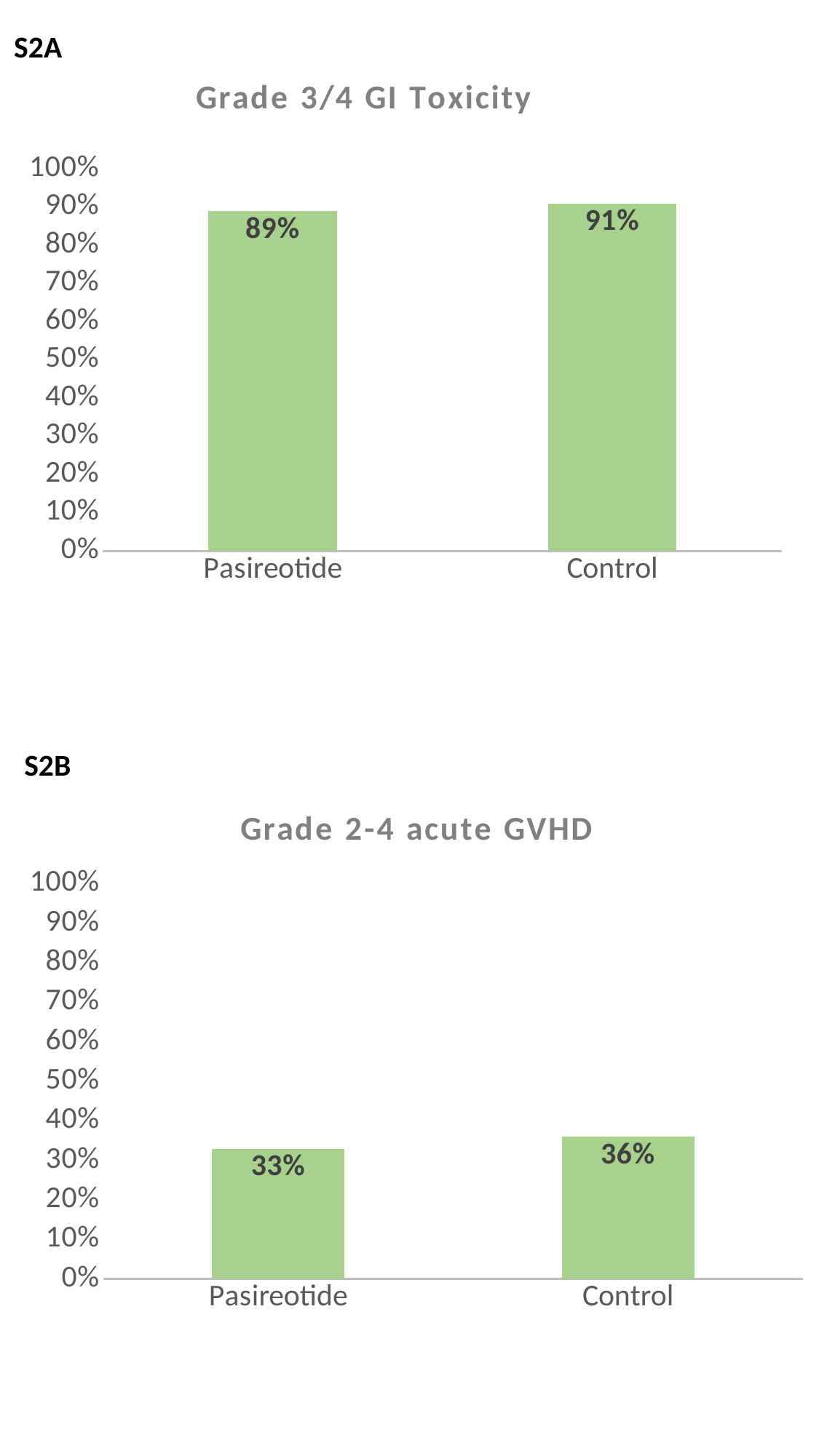

S2A
### Chart: Grade 3/4 GI Toxicity
| Category | Any GI toxicity |
|---|---|
| Pasireotide | 0.89 |
| Control | 0.91 |S2B
### Chart: Grade 2-4 acute GVHD
| Category | Any GI toxicity |
|---|---|
| Pasireotide | 0.33 |
| Control | 0.36 |

## Slide 4
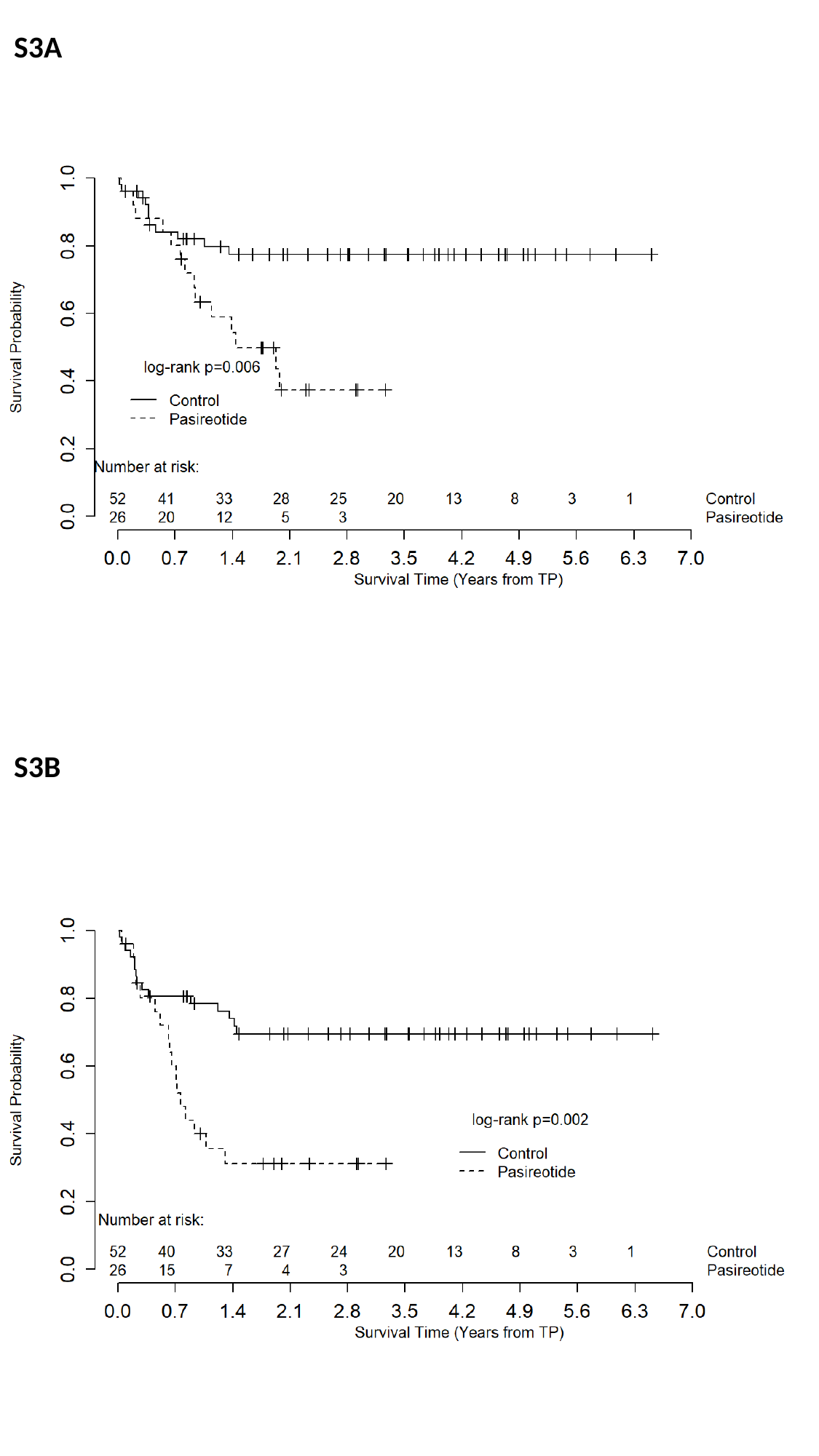

S3A
S3B
