# Supplementary material for: A phase 2 trial of the somatostatin analog pasireotide to prevent GI toxicity and acute GVHD in allogeneic hematopoietic stem cell transplant
Source: PLoS One. 2021 Jun 25;16(6):e0252995. doi: 10.1371/journal.pone.0252995 (PMC8232534; doi:10.1371/journal.pone.0252995)
Supplement: S2 File — *n = 25 is adjustment for one missing value; S3 Table. All toxicities with CTCAE frequency by treatment group. Percentages adjusted for missing values; S4 Table. Model predicting Shannon diversity incorporating anaerobic antibiotic coverage; S5 Table. Model predicting Shannon diversity incorporating febrile neutropenia; S6A Table. Pasireotide group inflammatory changes baseline to day 14 median/range followed by significance testing (54 plex panel); S6B Table. Pasireotide group inflammatory changes baseline to day 14 median/range followed by significance testing (Pepper panel); S7A Table. Pasireotide group inflammatory overall survival correlations for baseline and ratio of change from baseline to day 14 (54 plex panel); S7B Table. Pasireotide group inflammatory overall survival correlations for baseline and ratio of change from baseline to day 14 (Pepper panel); S8A Table. Pasireotide group inflammatory aGVHD correlations (54-plex panel); S8B Table. Pasireotide group inflammatory aGVHD correlations (Pepper panel); S9A Table. Pasireotide group inflammatory cGVHD correlations (54-plex panel); S9B Table. Pasireotide group inflammatory cGVHD correlations (Pepper panel); S10A Table. Pasireotide group inflammatory TRM correlations (54-plex panel); S10B Table: Pasireotide group inflammatory TRM correlations (Pepper panel); S11 Table. Pasireotide group metabolomics change from baseline to D14, median/range followed by significance testing. Conventional-C, Amino Acid-AA, Acylcarnitines-AC; S12 Table. Pasireotide group metabolomics correlation to overall survival Conventional-C, Amino Acid-AA, Acylcarnitines-AC; S13 Table. Pasireotide group metabolomics correlation to aGVHD Conventional-C, Amino Acid-AA, Acylcarnitines-AC; S14 Table. Pasireotide group metabolomics correlation to cGVHD Conventional-C, Amino Acid-AA, Acylcarnitines-AC; S15 Table. Pasireotide group metabolomics correlation to TRM Conventional-C, Amino Acid-AA, Acylcarnitines-AC. (DOCX) [file pone.0252995.s002.docx]

Table S1: Acute GVHD by site

| **Characteristic** | **Pasireotide**  **n=26**  **no. (%)** | **Control**  **n=52**  **no. (%)** |
| --- | --- | --- |
| Acute GVHD |  |  |
| Overall | 15 (58) | 28 (54) |
| G2-4 Overall | 10 (38) | 15(29) |
| Lower GI | 6 (23) | 6 (12) |
| UGI | 9 (35) | 11 (21) |
| Liver | 0 | 3 (6) |
| Skin | 13 (50) | 15 (29) |
| Other | 1 (4) | 0 |

Table S2: Chronic GVHD by site. *n=25 is adjustment for one missing value

| **Characteristic** | **Pasireotide**  **n=25***  **no. (%)** | **Control**  **n=52**  **no. (%)** |
| --- | --- | --- |
| Chronic GVHD |  |  |
| Overall | 16 (64) | 22 (42) |
| Severe (NIH Scale) | 8 (32) | 11 (21) |
| Skin | 13 (52) | 20 (38) |
| Eye | 6 (24) | 10 (19) |
| Mouth | 8 (32) | 12 (23) |
| GI | 7 (28) | 3 (6) |
| Liver | 8 (32) | 5 (10) |
| Lung | 1 (4) | 6 (12) |
| Joint | 2 (8) | 6 (12) |
| Genital | 1 (4) | 3 (6) |
| Other | 1 (4) | 0 |

Table S3: All toxicities with CTCAE frequency by treatment group; percentages adjusted for missing values

|  | **Pasireotide** | |  |  | **Cntl** |  |  |  |
| --- | --- | --- | --- | --- | --- | --- | --- | --- |
|  | **All** |  | **G3/4** |  | **All** |  | **G3/4** |  |
|  | **n** | **%** | **n** | **%** | **n** | **%** | **n** | **%** |
| **Nausea** | 25 | 100% | 5 | 20% | 43 | 83% | 0 | 0% |
| **Diarrhea** | 24 | 96% | 6 | 24% | 45 | 87% | 5 | 10% |
| **Mucositis** | 24 | 96% | 14 | 56% | 49 | 94% | 32 | 62% |
| **Anorexia** | 21 | 84% | 5 | 20% | 35 | 67% | 8 | 15% |
| **Fatigue** | 20 | 80% | 0 | 0% | 35 | 67% | 0 | 0% |
| **Vomiting** | 20 | 80% | 2 | 8% | 25 | 48% | 0 | 0% |
| **Rash** | 19 | 76% | 0 | 0% | 25 | 48% | 0 | 0% |
| **Febrile Neutropenia** | 17 | 68% | 17 | 68% | 33 | 63% | 33 | 63% |
| **Abdominal Pain** | 14 | 56% | 0 | 0% | 14 | 27% | 0 | 0% |
| **Hypertension** | 14 | 56% | 9 | 36% | 19 | 37% | 13 | 25% |
| **Hyperglycemia** | 12 | 48% | 11 | 44% | 15 | 29% | 11 | 21% |
| **Headache** | 11 | 44% | 0 | 0% | 25 | 48% | 1 | 2% |
| **QT prolongation** | 10 | 40% | 1 | 4% | 18 | 35% | 2 | 4% |
| **Dysgeusia** | 9 | 36% | 0 | 0% | 5 | 10% | 0 | 0% |
| **Constipation** | 8 | 32% | 0 | 0% | 14 | 27% | 0 | 0% |
| **Cough** | 8 | 32% | 0 | 0% | 8 | 15% | 0 | 0% |
| **Nasal Congestion** | 8 | 32% | 0 | 0% | 11 | 21% | 1 | 2% |
| **Anxiety** | 7 | 28% | 0 | 0% | 17 | 33% | 0 | 0% |
| **Sinus Bradycardia** | 7 | 28% | 0 | 0% | 3 | 6% | 0 | 0% |
| **Dyspnea** | 7 | 28% | 2 | 8% | 11 | 21% | 3 | 6% |
| **Hypoxia** | 7 | 28% | 5 | 20% | 12 | 23% | 9 | 17% |
| **Sepsis** | 7 | 28% | 7 | 28% | 5 | 10% | 5 | 10% |
| **Dysuria** | 6 | 24% | 0 | 0% | 10 | 19% | 0 | 0% |
| **Encephalopathy** | 6 | 24% | 2 | 8% | 10 | 19% | 3 | 6% |
| **Catheter-Related Infection** | 6 | 24% | 6 | 24% | 8 | 15% | 8 | 15% |
| **Bilirubin Elevation** | 6 | 24% | 2 | 8% | 4 | 8% | 4 | 8% |
| **Bloating** | 5 | 20% | 0 | 0% | 3 | 6% | 0 | 0% |
| **Dyspepsia** | 5 | 20% | 0 | 0% | 14 | 27% | 0 | 0% |
| **Enterocolitis** | 5 | 20% | 2 | 8% | 10 | 19% | 1 | 2% |
| **Inreased Creatinine** | 5 | 20% | 1 | 4% | 11 | 21% | 0 | 0% |
| **Hypotension** | 5 | 20% | 1 | 4% | 8 | 15% | 2 | 4% |
| **Fever** | 4 | 16% | 1 | 4% | 4 | 8% | 0 | 0% |
| **Insomnia** | 4 | 16% | 0 | 0% | 14 | 27% | 0 | 0% |
| **Lung Infection** | 4 | 16% | 4 | 16% | 7 | 13% | 5 | 10% |
| **Pulmonary Edema** | 4 | 16% | 3 | 12% | 2 | 4% | 1 | 2% |
| **Thrush** | 4 | 16% | 1 | 4% | 5 | 10% | 0 | 0% |
| **Viremia** | 4 | 16% | 2 | 8% | 12 | 23% | 10 | 19% |
| **Hallucination** | 3 | 12% | 0 | 0% | 5 | 10% | 0 | 0% |
| **HSV Reactivation** | 3 | 12% | 1 | 4% | 1 | 2% | 1 | 2% |
| **Hyperkalemia** | 3 | 12% | 0 | 0% | 6 | 12% | 1 | 2% |
| **Infusion Reaction** | 3 | 12% | 0 | 0% | 0 | 0% | 0 | 0% |
| **Rhinorrhea** | 3 | 12% | 0 | 0% | 5 | 10% | 0 | 0% |
| **Skin Infection** | 3 | 12% | 2 | 8% | 9 | 17% | 4 | 8% |
| **Urinary Retention** | 3 | 12% | 0 | 0% | 3 | 6% | 0 | 0% |
| **Allergic reaction** | 2 | 8% | 1 | 4% | 0 | 0% | 0 | 0% |
| **Chest pain** | 2 | 8% | 0 | 0% | 8 | 15% | 0 | 0% |
| **Dry Skin** | 2 | 8% | 0 | 0% | 0 | 0% | 0 | 0% |
| **Dry Eyes** | 2 | 8% | 0 | 0% | 1 | 2% | 0 | 0% |
| **Epistaxis** | 2 | 8% | 1 | 4% | 2 | 4% | 0 | 0% |
| **Peripheral Sensory Neuropathy** | 2 | 8% | 0 | 0% | 11 | 21% | 0 | 0% |
| **Pruritis** | 2 | 8% | 0 | 0% | 11 | 21% | 0 | 0% |
| **UTI** | 2 | 8% | 2 | 8% | 8 | 15% | 1 | 2% |
| **AST Elevation** | 2 | 8% | 1 | 4% | 6 | 12% | 0 | 0% |
| **ALT Elevation** | 2 | 8% | 0 | 0% | 5 | 10% | 2 | 4% |
| **Ileus** | 2 | 8% | 2 | 8% | 0 | 0% | 0 | 0% |
| **Dysphagia** | 1 | 4% | 0 | 0% | 2 | 4% | 0 | 0% |
| **Chills** | 1 | 4% | 0 | 0% | 3 | 6% | 0 | 0% |
| **2nd deg AVB** | 1 | 4% | 0 | 0% | 0 | 0% | 0 | 0% |
| **Bone Pain** | 1 | 4% | 0 | 0% | 2 | 4% | 0 | 0% |
| **Bladder Spasm** | 1 | 4% | 0 | 0% | 1 | 2% | 0 | 0% |
| **Dehydration** | 1 | 4% | 0 | 0% | 0 | 0% | 0 | 0% |
| **URI** | 1 | 4% | 0 | 0% | 2 | 4% | 0 | 0% |
| **Fracture** | 1 | 4% | 0 | 0% | 0 | 0% | 0 | 0% |
| **Hemoptysis** | 1 | 4% | 0 | 0% | 3 | 6% | 0 | 0% |
| **Hematuria** | 1 | 4% | 0 | 0% | 2 | 4% | 0 | 0% |
| **Hypernatremia** | 1 | 4% | 0 | 0% | 3 | 6% | 0 | 0% |
| **Hyponatremia** | 1 | 4% | 0 | 0% | 8 | 15% | 0 | 0% |
| **Otitis Media** | 1 | 4% | 0 | 0% | 0 | 0% | 0 | 0% |
| **Pain in Extremity** | 1 | 4% | 0 | 0% | 12 | 23% | 0 | 0% |
| **Pericardial effusion** | 1 | 4% | 0 | 0% | 1 | 2% | 0 | 0% |
| **Pleural Effusion** | 1 | 4% | 1 | 4% | **0** | 0% | 0 | 0% |
| **Prostatic Obstruction** | 1 | 4% | 0 | 0% | 0 | 0% | 0 | 0% |
| **Acne Rash** | 1 | 4% | 1 | 4% | 0 | 0% | 0 | 0% |
| **Toe Trauma** | 1 | 4% | 0 | 0% | 0 | 0% | 0 | 0% |
| **Thromboembolic event** | 1 | 4% | 0 | 0% | 3 | 6% | 0 | 0% |
| **Urinary incontinence** | 1 | 4% | 0 | 0% | 1 | 2% | 0 | 0% |
| **VOD** | 1 | 4% | 1 | 4% | 1 | 2% | 1 | 2% |
| **Alk Phos Elevation** | 1 | 4% | 0 | 0% | 0 | 0% | 0 | 0% |
| **Bowel Obstruction** | 1 | 4% | 1 | 4% | 0 | 0% | 0 | 0% |
| **Atrial Fibrillation** | 0 | 0% | 0 | 0% | 1 | 2% | 1 | 2% |
| **Acute Kidney Injury** | 0 | 0% | 0 | 0% | 2 | 4% | 2 | 4% |
| **Myocardial Infarction** | 0 | 0% | 0 | 0% | 1 | 2% | 0 | 0% |
| **Floaters** | 0 | 0% | 0 | 0% | 1 | 2% | 0 | 0% |
| **Folliculitis** | 0 | 0% | 0 | 0% | 0 | 0% | 0 | 0% |
| **Hiccups** | 0 | 0% | 0 | 0% | 4 | 8% | 0 | 0% |
| **Intracranial Hemorrhage** | 0 | 0% | 0 | 0% | 1 | 2% | 0 | 0% |
| **Hematoma** | 0 | 0% | 0 | 0% | 1 | 2% | 0 | 0% |
| **Myalgias** | 0 | 0% | 0 | 0% | 1 | 2% | 0 | 0% |
| **Joint Infection** | 0 | 0% | 0 | 0% | 1 | 2% | 0 | 0% |
| **Menorrhagia** | 0 | 0% | 0 | 0% | 2 | 4% | 1 | 2% |
| **Pneumonitis** | 0 | 0% | 0 | 0% | 4 | 8% | 0 | 0% |
| **PRES** | 0 | 0% | 0 | 0% | 2 | 4% | 2 | 4% |
| **Sinusitis** | 0 | 0% | 0 | 0% | 1 | 2% | 0 | 0% |

Table S4: Model predicting Shannon diversity incorporating anaerobic antibiotic coverage


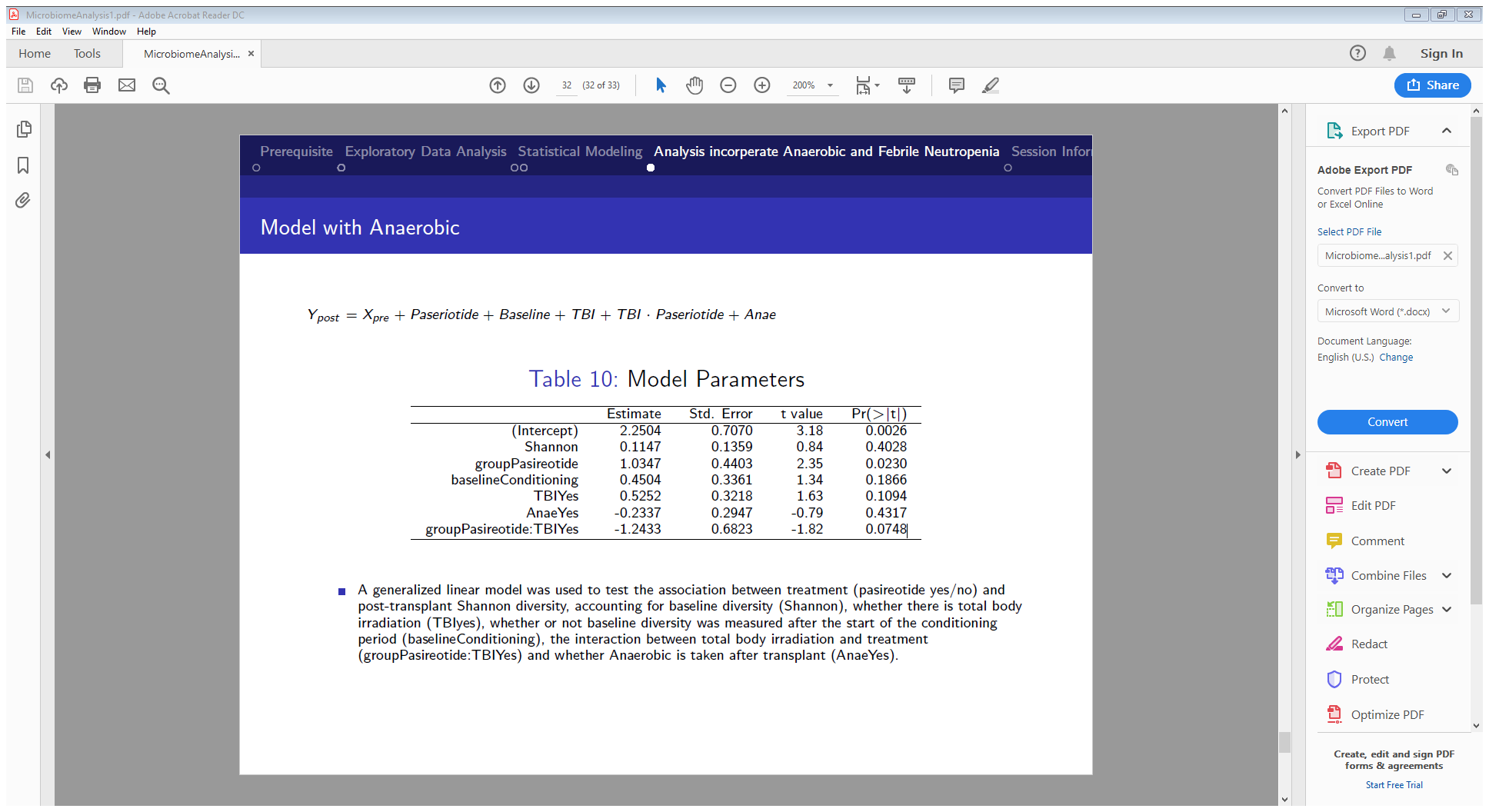


Table S5: Model predicting Shannon diversity incorporating febrile neutropenia


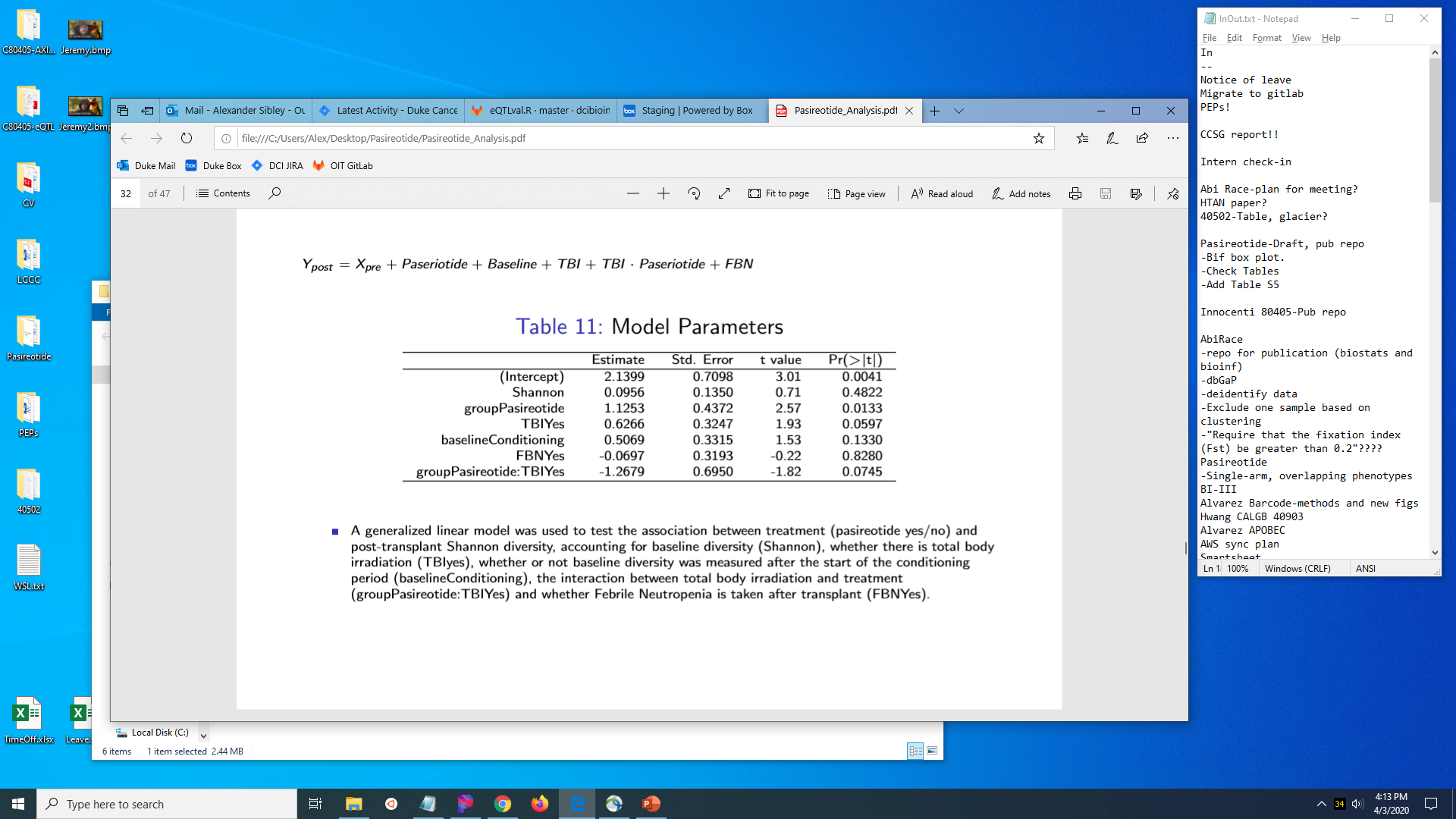


Table S6A. Pasireotide group inflammatory changes baseline to day 14 median/range followed by significance testing (54 plex panel)

Median/range:

| Biomarker | N.Pre | Median(range).Pre | N.D14 | Median(range).D14 |
| --- | --- | --- | --- | --- |
| bFGF | 20 | 3.9 (0.3 - 132.6) | 19 | 0.8 (0.3 - 17.3) |
| CRP | 20 | 6533.1 (309.6 - 144157.5) | 19 | 119055.5 (1520.2 - 144564.4) |
| Eotaxin | 20 | 145.2 (43 - 273.6) | 19 | 170.4 (83.3 - 275.4) |
| Eotaxin-3 | 20 | 9.1 (1.8 - 22) | 19 | 7.8 (4.5 - 113.9) |
| Flt-1 | 20 | 30.6 (17.4 - 2229.3) | 19 | 34.8 (17.2 - 1433.9) |
| GM-CSF | 13 | 0.1 (0 - 0.3) | 19 | 0.1 (0 - 1.3) |
| ICAM-1 | 20 | 515.2 (324.6 - 1278.5) | 19 | 574.2 (306.1 - 1303.1) |
| IFN-g | 20 | 7.3 (1.1 - 256.1) | 19 | 13 (3.1 - 258.7) |
| IL-10 | 20 | 0.8 (0.2 - 7.6) | 19 | 1.4 (0.3 - 40.8) |
| IL-12/IL-23p40 | 20 | 45.1 (16.3 - 178.9) | 19 | 7.5 (1.3 - 40.3) |
| IL-12p70 | 20 | 0.4 (0.1 - 4.3) | 19 | 0.4 (0.1 - 2.7) |
| IL-13 | 17 | 1.5 (0 - 31) | 17 | 1.6 (0.4 - 10.7) |
| IL-15 | 20 | 2 (1.1 - 7.8) | 19 | 19.5 (9.4 - 90.3) |
| IL-16 | 20 | 112.4 (36.9 - 212.2) | 19 | 55.2 (26.2 - 133.9) |
| IL-17A | 20 | 0.7 (0.2 - 2.7) | 19 | 0.7 (0.2 - 3.1) |
| IL-17A Gen B | 7 | 0.1 (0 - 0.3) | 5 | 0.1 (0 - 0.4) |
| IL-17A/F | 4 | 0.4 (0 - 0.8) | 1 | 0.3 (0.3 - 0.3) |
| IL-17D | 13 | 0.9 (0 - 9.1) | 9 | 0.2 (0 - 2.4) |
| IL-1b | 12 | 0.1 (0 - 80.5) | 16 | 0.1 (0 - 6.1) |
| IL-1RA | 20 | 57.1 (26.6 - 332.2) | 19 | 43.9 (11.8 - 191.7) |
| IL-2 | 20 | 0.7 (0.2 - 7.3) | 19 | 0.8 (0 - 3.4) |
| IL-21 | 8 | 0.5 (0 - 0.8) | 3 | 0.4 (0.3 - 1.5) |
| IL-22 | 18 | 0.1 (0 - 0.7) | 19 | 0.1 (0 - 0.9) |
| IL-27 | 20 | 797.4 (422.1 - 2352.5) | 19 | 925.9 (444.2 - 7204.1) |
| IL-31 | 8 | 0 (0 - 0.1) | 6 | 0 (0 - 0) |
| IL-4 | 19 | 0 (0 - 1.6) | 19 | 0 (0 - 0.5) |
| IL-5 | 4 | 0 (0 - 1.6) | 15 | 1 (0.1 - 10.1) |
| IL-6 | 20 | 2.2 (1 - 1481.7) | 19 | 27.6 (5.2 - 658.6) |
| IL-7 | 20 | 2.2 (1 - 5.5) | 19 | 4.4 (3.1 - 22.5) |
| IL-8.Pro | 20 | 30.4 (6.4 - 6977.3) | 19 | 173.3 (29 - 11933.7) |
| IL-9 | 2 | 0.3 (0.1 - 0.4) | 1 | 0 (0 - 0) |
| IP-10 | 20 | 99.5 (38.5 - 3792.3) | 19 | 104.8 (44.8 - 1296) |
| MCP-1 | 20 | 52.1 (29.4 - 599.7) | 19 | 239.8 (82.2 - 821.2) |
| MCP-4 | 20 | 56.3 (12.1 - 296.4) | 19 | 180.5 (36.2 - 566.2) |
| MDC | 20 | 127.4 (45 - 239.3) | 19 | 43.2 (18.4 - 146.3) |
| MIP-1a | 19 | 3.6 (0.7 - 456.8) | 18 | 5.5 (2.4 - 642.3) |
| MIP-1b | 20 | 17.9 (8.5 - 1352.7) | 19 | 33.6 (11.9 - 1504.6) |
| MIP-3a | 18 | 1.9 (0.2 - 17.3) | 18 | 3.1 (0.3 - 12.8) |
| PlGF | 20 | 5.3 (2.1 - 31.6) | 19 | 11.1 (4.6 - 26.9) |
| SAA | 20 | 5782 (508.5 - 218419.1) | 19 | 178245.5 (6158 - 212711.3) |
| TARC | 20 | 19.5 (2.7 - 106.7) | 19 | 7.7 (2 - 98.6) |
| Tie-2 | 20 | 2344.5 (1249.7 - 3259.5) | 19 | 1749.6 (1401.8 - 2403.3) |
| TNF-a | 20 | 6.3 (3 - 414.6) | 19 | 5.7 (2.3 - 179) |
| TNF-b | 15 | 0.1 (0 - 0.3) | 6 | 0 (0 - 0.3) |
| TSLP | 18 | 0.2 (0 - 0.6) | 18 | 0.5 (0.2 - 1) |
| VCAM-1 | 20 | 547.5 (450.9 - 1539.6) | 19 | 571.3 (281 - 1586) |
| VEGF-C | 20 | 14.4 (2.9 - 61.9) | 19 | 10.8 (3.8 - 23) |
| VEGF-D | 20 | 329.9 (203.1 - 592.8) | 19 | 443.2 (250.5 - 818.1) |
| VEGF.Angio | 20 | 46.6 (3.8 - 161.3) | 19 | 31.5 (3.2 - 248) |

Significance Testing:

| Marker | Estimated mean diff | P-value |
| --- | --- | --- |
| IL-15 | 3.204131 | 1.91e-14 |
| IL-12/IL-23p40 | -3.133895 | 2.18e-09 |
| CRP | 4.030925 | 8.35e-08 |
| SAA | 4.224968 | 2.77e-07 |
| MDC | -1.402647 | 1.38e-06 |
| MCP-1 | 1.949640 | 4.67e-06 |
| IL-7 | 1.431955 | 1.87e-05 |
| MCP-4 | 1.383078 | 3.81e-05 |
| Tie-2 | -0.375324 | 7.34e-05 |
| TSLP | 1.625077 | 0.000304 |
| VEGF-D | 0.542830 | 0.000305 |
| PlGF | 0.960063 | 0.000374 |
| IL-6 | 3.124650 | 0.000441 |
| IL-16 | -0.741357 | 0.000643 |
| IL-8.Pro | 2.244541 | 0.00436 |
| IL-27 | 0.470251 | 0.00611 |
| bFGF | -1.710040 | 0.0132 |
| VEGF-C | -0.661390 | 0.0235 |
| IL-12p70 | -0.524247 | 0.0393 |
| TARC | -1.095283 | 0.0374 |
| IL-17D | -2.277036 | 0.0431 |
| Eotaxin | 0.345915 | 0.0453 |
| MIP-3a | 0.806375 | 0.0802 |
| IL-1b | 2.314371 | 0.086 |
| IL-10 | 0.981052 | 0.0938 |
| IL-22 | -0.842518 | 0.0936 |
| TNF-b | -3.468137 | 0.154 |
| IL-13 | 0.827489 | 0.253 |
| IL-21 | 2.255904 | 0.259 |
| MIP-1b | 0.669480 | 0.262 |
| IL-17A | -0.360089 | 0.284 |
| MIP-1a | 0.745619 | 0.323 |
| VEGF.Angio | -0.400354 | 0.322 |
| IL-1RA | -0.294575 | 0.416 |
| IL-5 | 5.189689 | 0.432 |
| IFN-g | 0.440098 | 0.484 |
| IP-10 | -0.310587 | 0.496 |
| Flt-1 | -0.383222 | 0.521 |
| ICAM-1 | 0.075561 | 0.603 |
| VCAM-1 | -0.050230 | 0.653 |
| IL-2 | -0.133583 | 0.745 |
| IL-31 | 0.268564 | 0.822 |
| Eotaxin-3 | 0.055474 | 0.862 |
| IL-17A Gen B | -0.268231 | 0.935 |
| IL-4 | -0.033755 | 0.947 |
| TNF-a | 0.055276 | 0.916 |
| GM-CSF | -0.005962 | 0.994 |

Table S6B. Pasireotide group inflammatory changes baseline to day 14 median/range followed by significance testing (Pepper panel)

Median/Range:

| Biomarker | N.Pre | Median(range).Pre | N.D14 | Median(range).D14 |
| --- | --- | --- | --- | --- |
| MMP3 (pg/ml) | 20 | 10889.3857 (4709.1071 - 237898.8361) | 19 | 15736.6309 (4817.9467 - 50832.8957) |
| TNFRI (pg/ml) | 20 | 1475.7856 (849.6673 - 4240.5264) | 19 | 2145.1897 (1234.6176 - 14348.391) |
| TNFRII (pg/ml) | 20 | 7938.535 (4171.0223 - 20316.7696) | 19 | 8770.2166 (5553.605 - 72756.6884) |
| RANTES (pg/ml) | 20 | 41914.4758 (3122.0369 - 126635.2958) | 19 | 4322.9188 (797.5424 - 50529.8343) |
| IL1RL/ST2 (pg/ml) | 19 | 67.0119 (15.8177 - 131.8379) | 18 | 141.7028 (3.5586 - 514.3463) |
| IL6Ra (pg/ml) | 20 | 33432.5 (19697 - 55061) | 19 | 26715 (16928 - 35727) |
| D-dimer (ng/ml) | 20 | 466.115 (64.991 - 6820) | 19 | 1472.8 (495.3 - 6246.2) |
| REG3A (ng/ml) | 20 | 32.64 (10.231 - 85.242) | 19 | 33.309 (3.8317 - 414.78) |
| Paraoxonase (U/ul) | 20 | 0.0075 (0.0032 - 0.0167) | 19 | 0.0061 (0.0024 - 0.0158) |

Significance testing:

| Marker | Estimated mean diff | P-value |
| --- | --- | --- |
| IL6Ra (pg/ml) | -0.4332 | 4.45e-06 |
| TNFRI (pg/ml) | 0.6965 | 6.99e-05 |
| RANTES (pg/ml) | -2.3940 | 6.29e-05 |
| D-dimer (ng/ml) | 1.8018 | 0.000134 |
| IL1RL/ST2 (pg/ml) | 1.0751 | 0.00645 |
| MMP3 (pg/ml) | 0.5118 | 0.00861 |
| TNFRII (pg/ml) | 0.4287 | 0.0191 |
| Paraoxonase (U/ul) | -0.3167 | 0.0201 |
| REG3A (ng/ml) | 0.3713 | 0.217 |

Table S7A. Pasireotide group inflammatory overall survival correlations for baseline and ratio of change from baseline to day 14 (54 plex panel)

| Marker | HR-Base (95% CI) | P-Base | HR-Ratio (95% CI) | P-Ratio |
| --- | --- | --- | --- | --- |
| MIP-1b | 2.39 (1.37-4.15) | 0.002 | 8.71 (1.51-50.28) | 0.015 |
| TNF-a | 1.85 (1.22-2.79) | 0.004 | 2.64 (1.18-5.9) | 0.018 |
| IL-8.Pro | 2.08 (1.27-3.42) | 0.004 | 6 (1.02-35.22) | 0.047 |
| MIP-1a | 1.83 (1.19-2.84) | 0.006 | 1.16 (1.03-1.3) | 0.012 |
| IL-13 | 8.08 (1.72-38.06) | 0.008 | 1 (0.97-1.03) | 0.973 |
| IL-6 | 3.39 (1.2-9.56) | 0.021 | 1.38 (1.04-1.83) | 0.025 |
| IL-12p70 | 1.76 (1.07-2.88) | 0.026 | 0.66 (0.22-1.92) | 0.441 |
| TSLP | 3.5 (1.13-10.86) | 0.030 | 0.47 (0.04-5.32) | 0.540 |
| IL-22 | 2.53 (1.03-6.2) | 0.042 | 0.35 (0.09-1.3) | 0.116 |
| GM-CSF | 4.09 (0.99-16.85) | 0.051 | 0.4 (0.13-1.23) | 0.112 |
| CRP | 0.59 (0.33-1.04) | 0.067 | 0 (0-4.92) | 0.126 |
| IL-15 | 0.24 (0.04-1.33) | 0.103 | 0.8 (0.59-1.11) | 0.182 |
| IL-10 | 1.45 (0.88-2.38) | 0.145 | 0.97 (0.91-1.03) | 0.293 |
| IL-4 | 2.07 (0.74-5.81) | 0.166 | 0.11 (0.01-1.28) | 0.078 |
| MCP-1 | 0.6 (0.27-1.34) | 0.214 | 0.03 (0-2.55) | 0.120 |
| TNF-b | 3.67 (0.49-27.76) | 0.208 | 1.59 (0.37-6.88) | 0.538 |
| PlGF | 2.24 (0.54-9.27) | 0.266 | 1.04 (0.06-19.63) | 0.978 |
| Flt-1 | 1.33 (0.75-2.35) | 0.334 | 9.97 (0.8-123.81) | 0.074 |
| IL-1RA | 1.51 (0.63-3.62) | 0.357 | 5.58 (0.21-147.13) | 0.303 |
| Eotaxin-3 | 0.62 (0.23-1.66) | 0.342 | 0.9 (0.25-3.24) | 0.875 |
| IL-17A | 0.76 (0.4-1.45) | 0.403 | 0.9 (0.56-1.43) | 0.642 |
| IL-1b | 1.11 (0.79-1.56) | 0.552 | 0.07 (0-1.11) | 0.059 |
| IL-12/IL-23p40 | 0.74 (0.32-1.73) | 0.488 | 4.63 (0.41-52.51) | 0.217 |
| TARC | 1.4 (0.6-3.23) | 0.436 | 2.19 (0.65-7.36) | 0.204 |
| Eotaxin | 0.55 (0.1-2.99) | 0.490 | 0.01 (0-509.64) | 0.409 |
| IP-10 | 0.85 (0.52-1.4) | 0.525 | 0.27 (0.01-7.71) | 0.443 |
| IL-2 | 1.17 (0.7-1.95) | 0.545 | 0.89 (0.52-1.5) | 0.650 |
| VCAM-1 | 1.51 (0.44-5.23) | 0.512 | 0.32 (0-227518.26) | 0.869 |
| Tie-2 | 0.46 (0.02-12.91) | 0.649 | 0.13 (0-107044199039621344) | 0.923 |
| MIP-3a | 0.94 (0.54-1.66) | 0.844 | 1.07 (0.97-1.19) | 0.181 |
| IL-27 | 1.2 (0.47-3.04) | 0.708 | 294.57 (0-29757635.25) | 0.334 |
| MCP-4 | 0.93 (0.47-1.85) | 0.837 | 0.22 (0.01-7.74) | 0.405 |
| IFN-g | 1.04 (0.73-1.48) | 0.849 | 1.03 (0.94-1.13) | 0.490 |
| bFGF | 0.97 (0.7-1.35) | 0.857 | 1.02 (0.47-2.19) | 0.963 |
| IL-16 | 0.81 (0.25-2.62) | 0.726 | 1.15 (0-904.93) | 0.966 |
| SAA | 0.95 (0.56-1.61) | 0.843 | 0.67 (0.01-50.25) | 0.857 |
| VEGF-C | 0.9 (0.34-2.4) | 0.838 | 1.04 (0.03-33.37) | 0.980 |
| VEGF-D | 1.18 (0.25-5.66) | 0.838 | 0.29 (0-66052.16) | 0.844 |
| VEGF.Angio | 1.05 (0.45-2.42) | 0.913 | 3.37 (0.22-51.66) | 0.382 |
| MDC | 0.95 (0.33-2.75) | 0.925 | 4.59 (0-5715.45) | 0.675 |
| IL-7 | 0.96 (0.16-5.6) | 0.960 | 1 (0.98-1.03) | 0.722 |
| ICAM-1 | 1.01 (0.21-4.81) | 0.989 | 2.34 (0-101535.93) | 0.876 |
| IL-17A Gen B | NA | NA | NA | NA |
| IL-17D | NA | NA | NA | NA |
| IL-21 | NA | NA | NA | NA |
| IL-31 | NA | NA | NA | NA |
| IL-5 | NA | NA | NA | NA |

Table S7B. Pasireotide group inflammatory overall survival correlations for baseline and ratio of change from baseline to day 14 (Pepper panel)

| Marker | HR-Base (95% CI) | P-Base | HR-Ratio (95% CI) | P-Ratio |
| --- | --- | --- | --- | --- |
| D-dimer (ng/ml) | 0.27 (0.08-0.87) | 0.028 | 0 (0-0.22) | 0.018 |
| MMP3 (pg/ml) | 2.63 (0.91-7.55) | 0.073 | 0 (0-921.86) | 0.374 |
| TNFRII (pg/ml) | 1.2 (0.42-3.38) | 0.733 | 22.75 (0-1032120.64) | 0.568 |
| IL1RL/ST2 (pg/ml) | 0.81 (0.34-1.98) | 0.649 | 1.7 (0.19-15.37) | 0.637 |
| REG3A (ng/ml) | 0.75 (0.26-2.2) | 0.602 | 0.41 (0.02-8.03) | 0.557 |
| Paraoxonase (U/ul) | 0.66 (0.14-3.1) | 0.594 | 58.01 (0-7715745.73) | 0.500 |
| TNFRI (pg/ml) | 1.7 (0.49-5.95) | 0.407 | 4.48 (0-223380.73) | 0.786 |
| IL6Ra (pg/ml) | 1.6 (0.23-11.35) | 0.638 | 3.1 (0-44639398865193176) | 0.953 |
| RANTES (pg/ml) | 0.98 (0.59-1.62) | 0.928 | 27.96 (0.05-14757.93) | 0.298 |

Table S8A: Pasireotide group inflammatory aGVHD correlations (54-plex panel)

| Marker | OR-Base (95% CI) | P-Base | OR-Ratio (95% CI) | P-Ratio |
| --- | --- | --- | --- | --- |
| Flt-1 | 2.59 (0.69-9.71) | 0.157 | 2314.38 (0.09-58869221.82) | 0.134 |
| IL-22 | 2.28 (0.68-7.62) | 0.180 | 0.26 (0.05-1.4) | 0.116 |
| MIP-1b | 2.57 (0.77-8.61) | 0.126 | 47.24 (0.41-5424.37) | 0.111 |
| PlGF | 9929.94 (0.58-170826616.63) | 0.064 | 1713.01 (0.22-13287050.73) | 0.103 |
| TNF-a | 3.76 (0.72-19.65) | 0.116 | 42.01 (0.37-4733.67) | 0.121 |
| IL-8.Pro | 2.48 (0.71-8.67) | 0.153 | 6.55 (0.26-168.2) | 0.256 |
| IL-17A | 2.11 (0.64-6.99) | 0.220 | 1.42 (0.7-2.86) | 0.331 |
| IL-10 | 2.15 (0.84-5.51) | 0.113 | 0.93 (0.78-1.11) | 0.416 |
| IL-13 | 1.64 (0.76-3.54) | 0.206 | 0.95 (0.84-1.08) | 0.437 |
| VCAM-1 | 14.64 (0.48-445.12) | 0.123 | 1343.4 (0-720102529032.28) | 0.482 |
| IL-12p70 | 1.55 (0.75-3.17) | 0.234 | 1.33 (0.38-4.61) | 0.656 |
| IL-1RA | 1.93 (0.58-6.36) | 0.282 | 72.05 (0.23-22372.17) | 0.144 |
| IL-6 | 1.64 (0.66-4.05) | 0.286 | 1 (0.98-1.01) | 0.599 |
| GM-CSF | 2.16 (0.35-13.2) | 0.403 | 0.2 (0.03-1.65) | 0.136 |
| CRP | 0.56 (0.16-1.94) | 0.359 | 0 (0-69.04) | 0.233 |
| VEGF.Angio | 1.8 (0.47-6.93) | 0.392 | 17.48 (0.18-1709.46) | 0.221 |
| Tie-2 | 0.12 (0-15.46) | 0.392 | 0 (0-2.10925099623046e+22) | 0.799 |
| Eotaxin-3 | 0.49 (0.09-2.76) | 0.416 | 1.25 (0.1-15.76) | 0.865 |
| MCP-4 | 1.68 (0.45-6.34) | 0.443 | 0.38 (0-52.53) | 0.701 |
| IL-1b | 0.81 (0.32-2.01) | 0.646 | 0.03 (0-3.06) | 0.141 |
| IL-27 | 1.47 (0.17-12.79) | 0.728 | 28057928.82 (0.05-17060600902245170) | 0.097 |
| IL-4 | 0.95 (0.44-2.05) | 0.890 | 0.12 (0-4.45) | 0.251 |
| MDC | 0.79 (0.13-4.68) | 0.792 | 768.88 (0.02-29013121.77) | 0.217 |
| MIP-3a | 1.14 (0.5-2.59) | 0.752 | 2 (0.74-5.4) | 0.174 |
| VEGF-C | 0.98 (0.23-4.22) | 0.974 | 21.94 (0.1-4604.81) | 0.258 |
| MCP-1 | 0.71 (0.21-2.41) | 0.586 | 0.06 (0-10.21) | 0.281 |
| IL-2 | 1.02 (0.52-1.98) | 0.963 | 0.68 (0.31-1.48) | 0.334 |
| TARC | 0.7 (0.17-2.93) | 0.622 | 9.56 (0.13-716.28) | 0.305 |
| ICAM-1 | 1.11 (0.13-9.77) | 0.927 | 780.3 (0-18189217752.6) | 0.442 |
| TSLP | 1.08 (0.43-2.7) | 0.865 | 2.94 (0.13-67.71) | 0.500 |
| bFGF | 1.03 (0.66-1.62) | 0.897 | 1.19 (0.44-3.25) | 0.727 |
| IFN-g | 0.84 (0.47-1.5) | 0.559 | 1.12 (0.6-2.1) | 0.712 |
| IL-12/IL-23p40 | 0.83 (0.3-2.29) | 0.720 | 2.14 (0.03-161.65) | 0.729 |
| IL-7 | 1.62 (0.17-15.87) | 0.677 | 1.06 (0.86-1.32) | 0.580 |
| SAA | 1.03 (0.45-2.32) | 0.950 | 5.35 (0.01-4433.56) | 0.625 |
| Eotaxin | 0.65 (0.08-5.33) | 0.684 | 1.45 (0-2178215.37) | 0.959 |
| IL-15 | 0.9 (0.18-4.45) | 0.899 | 0.99 (0.85-1.15) | 0.927 |
| IP-10 | 1.04 (0.5-2.18) | 0.911 | 1.11 (0.01-162.56) | 0.968 |
| VEGF-D | 0.79 (0.06-10.65) | 0.860 | 1.61 (0-21725747.05) | 0.954 |
| IL-16 | 1.14 (0.2-6.34) | 0.882 | 1.01 (0-8643.6) | 0.998 |
| IL-17A Gen B | NA | NA | NA | NA |
| IL-17D | NA | NA | NA | NA |
| IL-21 | NA | NA | NA | NA |
| IL-31 | NA | NA | NA | NA |
| IL-5 | NA | NA | NA | NA |
| MIP-1a | NA | NA | NA | NA |
| TNF-b | NA | NA | NA | NA |

Table S8B: Pasireotide group inflammatory aGVHD correlations (Pepper panel)

| Marker | OR-Base (95% CI) | P-Base | OR-Ratio (95% CI) | P-Ratio |
| --- | --- | --- | --- | --- |
| MMP3 (pg/ml) | 1.55 (0.38-6.32) | 0.538 | 0 (0-8522.27) | 0.293 |
| RANTES (pg/ml) | 0.77 (0.32-1.88) | 0.572 | 530.33 (0.01-40309737.15) | 0.274 |
| IL1RL/ST2 (pg/ml) | 2.96 (0.31-28.4) | 0.348 | 429.97 (0.19-979216.63) | 0.124 |
| IL6Ra (pg/ml) | 8.54 (0.32-231.44) | 0.202 | 8.3806234061991e+19 (0-4.29054332116524e+48) | 0.174 |
| TNFRII (pg/ml) | 2.21 (0.32-15.33) | 0.423 | 17561.9 (0-139072980613147) | 0.401 |
| D-dimer (ng/ml) | 1.87 (0.43-8.06) | 0.401 | 0.49 (0-5120.2) | 0.880 |
| REG3A (ng/ml) | 1.48 (0.45-4.85) | 0.515 | 0.87 (0.02-32.02) | 0.938 |
| Paraoxonase (U/ul) | 0.68 (0.07-6.4) | 0.739 | 3472.15 (0-41457405020.93) | 0.327 |
| TNFRI (pg/ml) | 1.5 (0.19-11.79) | 0.699 | 53.12 (0-10574466932.6) | 0.684 |

Table S9A: Pasireotide group inflammatory cGVHD correlations (54-plex panel)

| Marker | OR-Base (95% CI) | P-Base | OR-Ratio (95% CI) | P-Ratio |
| --- | --- | --- | --- | --- |
| Eotaxin-3 | 2.88 (0.49-16.87) | 0.242 | 2.32 (0.21-25.63) | 0.491 |
| ICAM-1 | 12.8 (0.67-243.96) | 0.090 | 112.59 (0-1314176619.88) | 0.569 |
| IL-13 | 0.54 (0.24-1.2) | 0.131 | 0.92 (0.64-1.33) | 0.662 |
| IL-16 | 4.83 (0.63-37.01) | 0.129 | 8.14 (0-109710.61) | 0.666 |
| IL-17A | 2.21 (0.67-7.24) | 0.191 | 1.34 (0.69-2.6) | 0.395 |
| IL-1RA | 2.2 (0.66-7.34) | 0.201 | 13.57 (0.1-1894.73) | 0.301 |
| IP-10 | 2.45 (0.79-7.57) | 0.121 | 31.68 (0.07-13557.1) | 0.264 |
| MDC | 2.78 (0.46-16.62) | 0.264 | 0.02 (0-678.15) | 0.472 |
| PlGF | 4.34 (0.44-42.49) | 0.207 | 23.29 (0.27-2038.74) | 0.168 |
| TARC | 3.34 (0.61-18.27) | 0.165 | 1.61 (0.17-15.18) | 0.676 |
| TSLP | 1.75 (0.64-4.76) | 0.274 | 0.3 (0.01-6.31) | 0.438 |
| VCAM-1 | 42.29 (0.79-2256.89) | 0.065 | 161.55 (0-176469128817.07) | 0.632 |
| VEGF-C | 5.24 (0.79-34.54) | 0.085 | 73.25 (0.33-16412.83) | 0.120 |
| VEGF.Angio | 2.18 (0.54-8.89) | 0.276 | 0.52 (0.01-30.95) | 0.754 |
| IL-12/IL-23p40 | 2.47 (0.74-8.3) | 0.143 | 1.59 (0.03-84.11) | 0.818 |
| CRP | 1.47 (0.64-3.35) | 0.360 | 66.08 (0.03-139584.06) | 0.283 |
| GM-CSF | 2.57 (0.42-15.57) | 0.305 | 0.42 (0.08-2.2) | 0.305 |
| IFN-g | 1.53 (0.67-3.51) | 0.316 | 1.46 (0.27-7.74) | 0.660 |
| MIP-3a | 1.37 (0.69-2.72) | 0.371 | 0.93 (0.76-1.15) | 0.526 |
| TNF-b | 0.12 (0-9.05) | 0.339 | 0.42 (0.04-4.9) | 0.486 |
| MCP-4 | 0.59 (0.18-1.97) | 0.390 | 1.41 (0.01-270.11) | 0.898 |
| IL-12p70 | 0.78 (0.41-1.49) | 0.450 | 1.41 (0.45-4.36) | 0.553 |
| IL-1b | 1.34 (0.64-2.8) | 0.443 | 3.99 (0.04-389.57) | 0.554 |
| IL-8.Pro | 0.79 (0.44-1.43) | 0.432 | 0.25 (0.02-3.41) | 0.296 |
| bFGF | 1.11 (0.71-1.75) | 0.638 | 1.37 (0.51-3.7) | 0.533 |
| Flt-1 | 1.2 (0.48-3.02) | 0.694 | 16.69 (0.02-16301.21) | 0.423 |
| IL-6 | 0.88 (0.57-1.36) | 0.570 | 0.97 (0.88-1.08) | 0.592 |
| MIP-1b | 0.87 (0.48-1.55) | 0.627 | 0.36 (0.03-4.19) | 0.418 |
| SAA | 1.22 (0.51-2.9) | 0.659 | 95.64 (0.05-192918.6) | 0.240 |
| TNF-a | 1.22 (0.64-2.35) | 0.543 | 0.73 (0.21-2.52) | 0.619 |
| IL-2 | 0.87 (0.46-1.65) | 0.675 | 0.88 (0.43-1.81) | 0.730 |
| IL-15 | 0.73 (0.15-3.51) | 0.691 | 1 (0.86-1.16) | 0.991 |
| IL-10 | 0.89 (0.46-1.72) | 0.726 | 0.94 (0.8-1.09) | 0.409 |
| IL-4 | 0.91 (0.46-1.82) | 0.794 | 1.41 (0.52-3.86) | 0.502 |
| IL-27 | 1.24 (0.2-7.8) | 0.820 | 773299.28 (0.02-36768214223357.4) | 0.133 |
| MIP-1a | 1.03 (0.66-1.59) | 0.911 | 0.9 (0.7-1.16) | 0.418 |
| Tie-2 | 1.38 (0.01-200.63) | 0.900 | 0 (0-52674887120906.7) | 0.316 |
| MCP-1 | 0.95 (0.3-2.96) | 0.927 | 2.64 (0.02-362.78) | 0.699 |
| Eotaxin | 1.13 (0.16-8.09) | 0.901 | 0.54 (0-476681.59) | 0.930 |
| IL-22 | 1.02 (0.34-3.07) | 0.967 | 2.18 (0.36-13.22) | 0.395 |
| VEGF-D | 0.98 (0.08-12.6) | 0.989 | 0.51 (0-4366477.84) | 0.933 |
| IL-31 | 3.05 (0-Inf) | 1.000 | 0 (0-Inf) | 1.000 |
| IL-17A Gen B | NA | NA | NA | NA |
| IL-17D | NA | NA | NA | NA |
| IL-21 | NA | NA | NA | NA |
| IL-5 | NA | NA | NA | NA |
| IL-7 | NA | NA | NA | NA |

Table S9B: Pasireotide group inflammatory cGVHD correlations (Pepper panel)

| Marker | OR-Base (95% CI) | P-Base | OR-Ratio (95% CI) | P-Ratio |
| --- | --- | --- | --- | --- |
| IL1RL/ST2 (pg/ml) | 0.15 (0.03-0.91) | 0.039 | 16.82 (0.16-1763.85) | 0.234 |
| REG3A (ng/ml) | 8.98 (1.05-76.72) | 0.045 | 0.01 (0-2.3) | 0.095 |
| RANTES (pg/ml) | 1.9 (0.82-4.41) | 0.133 | 134.54 (0.01-1600855.17) | 0.306 |
| D-dimer (ng/ml) | 3.81 (0.75-19.4) | 0.107 | 877.31 (0.02-39362336.65) | 0.215 |
| TNFRII (pg/ml) | 4.85 (0.56-42.03) | 0.152 | 3127.66 (0-35213898574683.4) | 0.496 |
| TNFRI (pg/ml) | 2.3 (0.23-22.87) | 0.477 | 69952.87 (0-430084882072237) | 0.332 |
| IL6Ra (pg/ml) | 1.71 (0.09-33.55) | 0.722 | 0 (0-402949677402469696) | 0.574 |
| MMP3 (pg/ml) | 0.98 (0.26-3.7) | 0.979 | 0 (0-47464.75) | 0.451 |
| Paraoxonase (U/ul) | 1.03 (0.12-8.59) | 0.978 | 12.95 (0-25971184.14) | 0.729 |

Table S10A: Pasireotide group inflammatory TRM correlations (54-plex panel)

| Marker | OR-Base (95% CI) | P-Base | OR-Ratio (95% CI) | P-Ratio |
| --- | --- | --- | --- | --- |
| bFGF | 2.1 (0.7-6.3) | 0.186 | 11.32 (0.62-208.21) | 0.102 |
| IL-1RA | 4 (0.47-33.74) | 0.202 | 834.99 (0.12-6021393.57) | 0.138 |
| MCP-4 | 0.14 (0.01-1.5) | 0.103 | 0 (0-120.21) | 0.175 |
| MDC | 4.85 (0.52-45.66) | 0.168 | 23923.85 (0.01-104530750407.76) | 0.196 |
| MIP-1b | 2.06 (0.94-4.52) | 0.070 | 11.54 (0.42-314.71) | 0.147 |
| TNF-a | 2.65 (0.97-7.24) | 0.058 | 6.26 (0.81-48.48) | 0.079 |
| VEGF.Angio | 5.36 (0.69-41.82) | 0.109 | 268.23 (0.45-160960.62) | 0.087 |
| IL-8.Pro | 1.73 (0.83-3.59) | 0.140 | 6.18 (0.26-147.12) | 0.260 |
| Flt-1 | 0.01 (0-2.74) | 0.106 | 0.03 (0-119077.78) | 0.640 |
| IFN-g | 1.66 (0.77-3.6) | 0.200 | 0.95 (0.39-2.33) | 0.905 |
| MIP-1a | 1.36 (0.85-2.17) | 0.199 | 1.01 (0.84-1.22) | 0.903 |
| IL-1b | 1.86 (0.59-5.89) | 0.291 | 0.01 (0-7.38) | 0.173 |
| PlGF | 0.11 (0-4.15) | 0.235 | 0 (0-13.23) | 0.175 |
| Eotaxin | 12.79 (0.1-1705.15) | 0.307 | 4175.06 (0-115117344598300) | 0.497 |
| IL-22 | 2.36 (0.44-12.55) | 0.314 | 0.5 (0.07-3.41) | 0.476 |
| Tie-2 | 0.02 (0-22.85) | 0.265 | 0 (0-1.10752701845739e+22) | 0.426 |
| ICAM-1 | 5.66 (0.18-173.87) | 0.321 | 0.07 (0-34746884241.56) | 0.848 |
| IL-2 | 0.62 (0.22-1.71) | 0.353 | 0.94 (0.36-2.46) | 0.902 |
| SAA | 2.3 (0.34-15.41) | 0.391 | 407.05 (0-10848873585.45) | 0.491 |
| IL-7 | 2.02 (0.13-32.24) | 0.619 | 1.03 (0.98-1.09) | 0.200 |
| VEGF-C | 1.58 (0.21-11.97) | 0.661 | 29.06 (0.05-16397.85) | 0.297 |
| VCAM-1 | 0.35 (0.01-17.76) | 0.597 | 2986763.39 (0-23961655106311323648) | 0.325 |
| IL-10 | 0.82 (0.29-2.33) | 0.705 | 0.95 (0.84-1.08) | 0.466 |
| MIP-3a | 1.74 (0.34-8.89) | 0.508 | 1.47 (0.58-3.72) | 0.417 |
| Eotaxin-3 | 0.64 (0.07-5.93) | 0.692 | 0.55 (0.02-12.32) | 0.703 |
| IL-17A | 0.62 (0.15-2.57) | 0.506 | 0.81 (0.31-2.1) | 0.659 |
| IL-4 | 1.38 (0.54-3.52) | 0.502 | 1.3 (0.52-3.25) | 0.576 |
| IL-6 | 1.1 (0.7-1.74) | 0.674 | 1.02 (0.93-1.11) | 0.698 |
| IL-13 | 1.18 (0.71-1.95) | 0.524 | 0.96 (0.76-1.22) | 0.745 |
| CRP | 1.63 (0.35-7.57) | 0.532 | 4.93 (0-14669888.88) | 0.834 |
| IL-12p70 | 1.2 (0.53-2.75) | 0.659 | 0.99 (0.25-3.99) | 0.987 |
| IL-27 | 0.64 (0.07-5.69) | 0.691 | 0.92 (0-1215554748.51) | 0.994 |
| IL-16 | 1.52 (0.12-18.92) | 0.747 | 920.95 (0.01-125471108.32) | 0.258 |
| GM-CSF | 0.76 (0.1-6.13) | 0.800 | 1.51 (0.25-9.25) | 0.654 |
| IP-10 | 0.9 (0.34-2.41) | 0.834 | 0.21 (0-336.68) | 0.679 |
| VEGF-D | 0.8 (0.02-39.61) | 0.910 | 0 (0-8317245.72) | 0.534 |
| IL-17D | 1.03 (0.28-3.83) | 0.965 | 1.29 (0.72-2.31) | 0.397 |
| IL-31 | 1 (0-Inf) | 1.000 | 1 (0-Inf) | 1.000 |
| IL-12/IL-23p40 | NA | NA | NA | NA |
| IL-15 | NA | NA | NA | NA |
| IL-17A Gen B | NA | NA | NA | NA |
| IL-21 | NA | NA | NA | NA |
| IL-5 | NA | NA | NA | NA |
| MCP-1 | NA | NA | NA | NA |
| TARC | NA | NA | NA | NA |
| TNF-b | NA | NA | NA | NA |
| TSLP | NA | NA | NA | NA |

Table S10B: Pasireotide group inflammatory TRM correlations (Pepper panel)

| Marker | OR-Base (95% CI) | P-Base | OR-Ratio (95% CI) | P-Ratio |
| --- | --- | --- | --- | --- |
| MMP3 (pg/ml) | 2.63 (0.34-20.2) | 0.353 | 761416.79 (0-420146465843897024) | 0.326 |
| RANTES (pg/ml) | 11.87 (0.12-1163.2) | 0.290 | 53459098536161681418 (0-9.69635372470697e+57) | 0.312 |
| IL1RL/ST2 (pg/ml) | 0.48 (0.11-2.11) | 0.334 | 0.2 (0-9.51) | 0.417 |
| D-dimer (ng/ml) | 0.6 (0.1-3.58) | 0.576 | 0 (0-1528.45) | 0.330 |
| Paraoxonase (U/ul) | 1.82 (0.12-26.91) | 0.664 | 0 (0-2323693.45) | 0.527 |
| TNFRI (pg/ml) | 1.35 (0.1-17.45) | 0.820 | 0.28 (0-2134597457.63) | 0.912 |
| TNFRII (pg/ml) | 0.84 (0.11-6.53) | 0.866 | 141.82 (0-717488634648.7) | 0.664 |
| IL6Ra (pg/ml) | 2.03 (0.04-93.93) | 0.718 | 6.08 (0-1.0642917538371e+34) | 0.963 |
| REG3A (ng/ml) | 0.99 (0.22-4.5) | 0.986 | 2.11 (0.02-249.58) | 0.759 |

Table S11: Pasireotide group metabolomics change from baseline to D14, median/range followed by significance testing (Conventional, Amino Acid-AA, Acylcarnitines-AC)

Median/range

| Biomarker | N.Pre | Median(range).Pre | N.D14 | Median(range).D14 |
| --- | --- | --- | --- | --- |
| HBUT | 20 | 23.95 (10 - 219.6) | 19 | 25.5 (5.4 - 1195.1) |
| LACT | 20 | 2.55 (0.9 - 9.4) | 19 | 2.9 (0.7 - 4.5) |
| NEFA | 20 | 0.48 (0.15 - 1.25) | 19 | 0.58 (0.12 - 1.46) |
| TG-B | 20 | 118 (57 - 288) | 19 | 134 (61 - 281) |
| Glycerol | 20 | 0.485 (0.12 - 2.17) | 19 | 0.9 (0.13 - 2.39) |
| Gly | 20 | 256.0021 (142.5214 - 520.816) | 19 | 176.7743 (145.8621 - 497.9013) |
| Ala | 20 | 348.7707 (227.4553 - 479.3152) | 19 | 253.8248 (171.7797 - 378.1953) |
| Ser | 20 | 93.4702 (56.7379 - 159.7095) | 19 | 92.7004 (63.4555 - 150.7226) |
| Pro | 20 | 253.255 (174.2531 - 370.8679) | 19 | 137.0511 (66.7229 - 245.8402) |
| Val | 20 | 245.4076 (135.553 - 378.3829) | 19 | 289.9403 (183.9655 - 458.8643) |
| Leu/Ile | 20 | 174.9673 (116.0078 - 325.4459) | 19 | 156.5679 (113.7713 - 255.8592) |
| Met | 20 | 23.6928 (14.1255 - 44.6465) | 19 | 19.7947 (10.2932 - 48.924) |
| His | 20 | 78.5421 (47.973 - 112.4417) | 19 | 67.4494 (48.8951 - 103.4059) |
| Phe | 20 | 63.9767 (43.3352 - 108.0441) | 19 | 130.3545 (64.7003 - 662.9399) |
| Tyr | 20 | 61.7545 (41.403 - 122.7967) | 19 | 66.2509 (34.8971 - 99.0059) |
| Asx | 20 | 12.4049 (8.2755 - 18.2516) | 19 | 8.6716 (5.5347 - 11.5582) |
| Glx | 20 | 125.6141 (92.532 - 255.0449) | 19 | 104.726 (77.1977 - 166.1457) |
| Orn | 20 | 120.9775 (76.173 - 163.6922) | 19 | 103.3367 (66.0259 - 276.6608) |
| Cit | 20 | 29.8694 (14.2978 - 56.7306) | 19 | 9.0788 (3.8718 - 23.8409) |
| Arg | 20 | 46.3397 (9.4125 - 114.437) | 19 | 36.1453 (9.7334 - 96.2199) |
| C2 | 20 | 5.0805 (2.9339 - 10.3061) | 19 | 5.2698 (0.935 - 12.4137) |
| C3 | 20 | 0.3625 (0.0772 - 0.6626) | 19 | 0.2532 (0.0999 - 0.5364) |
| C4/Ci4 | 20 | 0.1861 (0.0453 - 0.3195) | 19 | 0.12 (0.0479 - 0.4305) |
| C5:1 | 20 | 0.0408 (0.0226 - 0.0699) | 19 | 0.0321 (0.0203 - 0.0544) |
| C5 | 20 | 0.1235 (0.0429 - 0.3642) | 19 | 0.0679 (0.0313 - 0.1602) |
| C4-OH | 20 | 0.0206 (0.0077 - 0.0837) | 17 | 0.0249 (0.0034 - 0.1471) |
| C6 | 20 | 0.0391 (0.0246 - 0.0986) | 19 | 0.0358 (0.0161 - 0.0867) |
| C5-OH/C3-DC | 20 | 0.0312 (0.007 - 0.0517) | 19 | 0.025 (0.0126 - 0.0762) |
| C4-DC/Ci4-DC | 20 | 0.0381 (0.0179 - 0.0582) | 19 | 0.0259 (0.0102 - 0.0515) |
| C8:1 | 20 | 0.3034 (0.0644 - 0.5862) | 19 | 0.105 (0.0528 - 0.5176) |
| C8 | 20 | 0.0748 (0.0161 - 0.1158) | 19 | 0.0449 (0.0064 - 0.1164) |
| C5-DC | 20 | 0.0406 (0.0208 - 0.103) | 19 | 0.0342 (0.0121 - 0.1112) |
| C8:1-OH/C6:1-DC | 20 | 0.0265 (0.0075 - 0.0465) | 19 | 0.0138 (0.0063 - 0.0429) |
| C6-DC/C8-OH | 20 | 0.0526 (0.0243 - 0.1091) | 19 | 0.0605 (0.0211 - 0.1226) |
| C10:3 | 20 | 0.0951 (0.0241 - 0.15) | 19 | 0.052 (0.0284 - 0.2089) |
| C10:2 | 20 | 0.023 (0.0067 - 0.0438) | 19 | 0.0138 (0.0053 - 0.039) |
| C10:1 | 20 | 0.1005 (0.0261 - 0.1754) | 19 | 0.0766 (0.0229 - 0.2316) |
| C10 | 20 | 0.1105 (0.0366 - 0.1849) | 19 | 0.0616 (0.0137 - 0.1809) |
| C7-DC | 19 | 0.0102 (0.0036 - 0.0222) | 18 | 0.0113 (0.0012 - 0.0223) |
| C8:1-DC | 20 | 0.0271 (0.0098 - 0.0654) | 19 | 0.0168 (0.0045 - 0.0442) |
| C10-OH/C8-DC | 20 | 0.0212 (0.0093 - 0.0732) | 19 | 0.0202 (0.0043 - 0.0441) |
| C12:1 | 20 | 0.0622 (0.0192 - 0.1134) | 19 | 0.0471 (0.0073 - 0.1025) |
| C12 | 20 | 0.0577 (0.0214 - 0.1362) | 19 | 0.037 (0.0068 - 0.0745) |
| C12-OH/C10-DC | 20 | 0.0061 (0.0026 - 0.0167) | 19 | 0.0045 (0.0011 - 0.0135) |
| C14:2 | 20 | 0.0369 (0.009 - 0.07) | 19 | 0.028 (0.0017 - 0.067) |
| C14:1 | 20 | 0.0501 (0.0173 - 0.1051) | 19 | 0.0402 (0.006 - 0.0912) |
| C14 | 20 | 0.025 (0.0137 - 0.0651) | 19 | 0.0186 (0.0056 - 0.0347) |
| C14:1-OH | 20 | 0.0135 (0.0062 - 0.027) | 19 | 0.0086 (0.0015 - 0.0223) |
| C14-OH/C12-DC | 19 | 0.009 (0.0036 - 0.0148) | 18 | 0.0058 (0.0026 - 0.0653) |
| C16:2 | 20 | 0.0077 (0.0015 - 0.0199) | 19 | 0.0054 (4e-04 - 0.0147) |
| C16:1 | 20 | 0.0221 (0.0084 - 0.0647) | 19 | 0.019 (0.0013 - 0.0379) |
| C16 | 20 | 0.1066 (0.0433 - 0.2717) | 19 | 0.0622 (0.0167 - 0.1449) |
| C16:1-OH/C14:1-DC | 20 | 0.0064 (0.0025 - 0.0172) | 19 | 0.0061 (0.0017 - 0.014) |
| C16-OH/C14-DC | 19 | 0.0038 (0.0017 - 0.0207) | 19 | 0.0046 (0.0015 - 0.0086) |
| C18:2 | 20 | 0.1122 (0.0241 - 0.4322) | 19 | 0.0482 (0.0122 - 0.1037) |
| C18:1 | 20 | 0.1687 (0.0394 - 0.5768) | 19 | 0.0787 (0.0142 - 0.236) |
| C18 | 20 | 0.0469 (0.0179 - 0.1325) | 19 | 0.0216 (0.008 - 0.0542) |
| C18:2-OH | 19 | 0.0035 (0.0014 - 0.0101) | 16 | 0.0034 (6e-04 - 0.0082) |
| C18:1-OH/C16:1-DC | 19 | 0.0041 (0.0017 - 0.014) | 19 | 0.0045 (0.0025 - 0.0142) |
| C18-OH/C16-DC | 20 | 0.0067 (0.0029 - 0.0307) | 19 | 0.0078 (0.0016 - 0.0179) |
| C20:4 | 20 | 0.0104 (0.001 - 0.0635) | 17 | 0.0049 (0.0021 - 0.007) |
| C20 | 20 | 0.0039 (0.0016 - 0.0095) | 18 | 0.0027 (0.001 - 0.006) |
| C18:1-DC | 20 | 0.0061 (0.0017 - 0.0237) | 19 | 0.006 (0.0016 - 0.0169) |
| C20-OH/C18-DC | 20 | 0.0062 (0.0025 - 0.0332) | 19 | 0.0052 (0.001 - 0.0103) |
| C22 | 19 | 0.0019 (0.001 - 0.0051) | 18 | 0.0016 (8e-04 - 0.0041) |

Significance testing:

| Marker (Conventional) | Estimated mean diff | P-value |
| --- | --- | --- |
| Glycerol | 0.61093 | 0.111 |
| HBUT | 0.38201 | 0.459 |
| LACT | 0.06049 | 0.782 |
| NEFA | 0.20485 | 0.491 |
| TG-B | 0.03643 | 0.801 |

| Marker (AA) | Estimated mean diff | P-value |
| --- | --- | --- |
| Cit | -1.61268 | 1.05e-08 |
| Pro | -0.93292 | 1.55e-07 |
| Ala | -0.47784 | 3.24e-06 |
| Gly | -0.48614 | 2.82e-05 |
| Phe | 1.13682 | 3.4e-05 |
| Asx | -0.59670 | 9.18e-05 |
| Val | 0.25491 | 0.00416 |
| Glx | -0.21306 | 0.0123 |
| His | -0.15865 | 0.0921 |
| Arg | -0.28413 | 0.17 |
| Leu/Ile | -0.10763 | 0.193 |
| Met | -0.10339 | 0.463 |
| Tyr | 0.09205 | 0.429 |
| Orn | -0.07344 | 0.582 |
| Ser | -0.01818 | 0.86 |

| Marker (AC) | Estimated mean diff | P-value |
| --- | --- | --- |
| C8:1 | -1.180250 | 6.56e-05 |
| C18 | -1.229776 | 3.92e-05 |
| C8:1-OH/C6:1-DC | -0.748137 | 0.000412 |
| C8:1-DC | -0.726003 | 0.000826 |
| C16 | -0.948834 | 0.000902 |
| C18:2 | -1.265114 | 0.00137 |
| C5 | -0.725679 | 0.00206 |
| C18:1 | -1.040924 | 0.00289 |
| C5:1 | -0.336095 | 0.0034 |
| C14 | -0.810603 | 0.00388 |
| C12 | -0.856540 | 0.00515 |
| C4-DC/Ci4-DC | -0.413064 | 0.00642 |
| C14:1-OH | -0.683500 | 0.00622 |
| C20:4 | -1.181388 | 0.00866 |
| C10:3 | -0.530813 | 0.0132 |
| C20 | -0.584990 | 0.0181 |
| C10 | -0.604916 | 0.0261 |
| C10:2 | -0.485488 | 0.028 |
| C20-OH/C18-DC | -0.497320 | 0.0294 |
| C3 | -0.410864 | 0.0327 |
| C8 | -0.586820 | 0.0396 |
| C22 | -0.419219 | 0.0487 |
| C10-OH/C8-DC | -0.542098 | 0.0641 |
| C16:1 | -0.637791 | 0.0634 |
| C16:1-OH/C14:1-DC | -0.456813 | 0.0646 |
| C14:2 | -0.631316 | 0.0912 |
| C12:1 | -0.497348 | 0.104 |
| C6 | -0.275847 | 0.113 |
| C12-OH/C10-DC | -0.495091 | 0.123 |
| C14:1 | -0.485535 | 0.131 |
| C16:2 | -0.559930 | 0.133 |
| C7-DC | -0.385109 | 0.145 |
| C4/Ci4 | -0.279436 | 0.173 |
| C5-DC | -0.232958 | 0.172 |
| C2 | -0.257978 | 0.293 |
| C10:1 | -0.174499 | 0.415 |
| C16-OH/C14-DC | -0.192741 | 0.473 |
| C4-OH | -0.264235 | 0.524 |
| C18:1-OH/C16:1-DC | 0.112480 | 0.586 |
| C18:2-OH | -0.143503 | 0.631 |
| C14-OH/C12-DC | -0.112927 | 0.757 |
| C18-OH/C16-DC | -0.062231 | 0.768 |
| C5-OH/C3-DC | 0.032479 | 0.844 |
| C6-DC/C8-OH | -0.005600 | 0.979 |
| C18:1-DC | -0.009942 | 0.97 |

Table S12: Pasireotide group metabolomics correlation to overall survival (Conventional-C, Amino Acid-AA, Acylcarnitines-AC)

| Marker (C) | HR-Base (95% CI) | P-Base | HR-Ratio (95% CI) | P-Ratio |
| --- | --- | --- | --- | --- |
| TG-B | 0.32 (0.06-1.68) | 0.176 | 0.34 (0-36031.18) | 0.857 |
| HBUT | 1.08 (0.6-1.93) | 0.800 | 3.56 (0.67-18.99) | 0.138 |
| LACT | 1.26 (0.53-3.03) | 0.603 | 0.82 (0.61-1.1) | 0.188 |
| Glycerol | 0.86 (0.47-1.6) | 0.644 | 0.84 (0.58-1.22) | 0.355 |
| NEFA | 0.95 (0.42-2.17) | 0.905 | 1.14 (0.78-1.67) | 0.497 |

| Marker (AA) | HR-Base (95% CI) | P-Base | HR-Ratio (95% CI) | P-Ratio |
| --- | --- | --- | --- | --- |
| Gly | 11.33 (0.9-142.49) | 0.060 | 119232724.58 (0.26-53814879257250456) | 0.067 |
| Phe | 37.46 (0.92-1520.37) | 0.055 | 0.01 (0-2.05) | 0.089 |
| Orn | 12.68 (0.73-221.57) | 0.082 | 1104127.89 (8.71-139960227065.79) | 0.020 |
| Cit | 4.71 (0.73-30.22) | 0.103 | 230.7 (0.93-57166.92) | 0.053 |
| Asx | 9.02 (0.39-209.42) | 0.170 | 4261.14 (0.79-23001324.06) | 0.057 |
| Tyr | 8.77 (0.44-173.39) | 0.154 | 113.47 (0.01-1871600.96) | 0.340 |
| Pro | 3.57 (0.29-44.42) | 0.323 | 31107 (0.23-4194561594.7) | 0.086 |
| Met | 2.72 (0.4-18.32) | 0.303 | 0.45 (0.01-34.63) | 0.720 |
| Val | 2.63 (0.09-73.86) | 0.570 | 0 (0-2.76) | 0.066 |
| His | 0.39 (0.01-12.25) | 0.589 | 0 (0-92.23) | 0.202 |
| Arg | 1.5 (0.5-4.52) | 0.466 | 6.42 (0.11-366.35) | 0.368 |
| Leu/Ile | 2.21 (0.1-47.29) | 0.613 | 0 (0-334920.07) | 0.481 |
| Ser | 1.8 (0.14-23.69) | 0.654 | 26.38 (0-41936581.05) | 0.653 |
| Ala | 1.7 (0.18-15.71) | 0.640 | 2.57 (0-363834439.3) | 0.922 |
| Glx | 0.65 (0.05-8.57) | 0.742 | 9530.89 (0-332099295890.95) | 0.301 |

| Marker (AC) | HR-Base (95% CI) | P-Base | HR-Ratio (95% CI) | P-Ratio |
| --- | --- | --- | --- | --- |
| C20-OH/C18-DC | 12.43 (1.34-115.48) | 0.027 | 0 (0-0.03) | 0.011 |
| C22 | 6.73 (1.19-38.24) | 0.031 | 0 (0-0) | 0.005 |
| C16:1-OH/C14:1-DC | 2.03 (0.54-7.72) | 0.297 | 0 (0-0.34) | 0.022 |
| C6-DC/C8-OH | 2.58 (0.64-10.39) | 0.182 | 0.02 (0-1.01) | 0.051 |
| C10-OH/C8-DC | 3.56 (0.74-17.08) | 0.112 | 0.01 (0-1.06) | 0.053 |
| C14:1-OH | 2.32 (0.56-9.58) | 0.244 | 0.01 (0-1.25) | 0.060 |
| C16-OH/C14-DC | 3.52 (0.75-16.5) | 0.111 | 0 (0-1.22) | 0.057 |
| C18-OH/C16-DC | 1.47 (0.44-4.96) | 0.535 | 0 (0-2.75) | 0.092 |
| C18:1-DC | 1.75 (0.58-5.27) | 0.320 | 0 (0-2.2) | 0.086 |
| C2 | 1.54 (0.37-6.46) | 0.553 | 3.84 (0.51-29.2) | 0.193 |
| C3 | 1.39 (0.52-3.76) | 0.511 | 0.34 (0.07-1.68) | 0.184 |
| C5-OH/C3-DC | 1.36 (0.5-3.74) | 0.547 | 0.07 (0-5.54) | 0.235 |
| C8 | 1.62 (0.38-6.89) | 0.517 | 0.19 (0.02-2.26) | 0.189 |
| C12 | 1.59 (0.46-5.49) | 0.460 | 0.15 (0.01-2.67) | 0.198 |
| C14-OH/C12-DC | 1.69 (0.53-5.32) | 0.372 | 0.14 (0-4.37) | 0.263 |
| C16 | 1.81 (0.5-6.6) | 0.369 | 0.22 (0.02-2.46) | 0.220 |
| C18 | 2.52 (0.64-9.89) | 0.186 | 0.09 (0-3.83) | 0.209 |
| C20 | 1.7 (0.45-6.44) | 0.432 | 0.01 (0-17.23) | 0.232 |
| C4-DC/Ci4-DC | 1.93 (0.46-8.05) | 0.366 | 0.04 (0-16.15) | 0.290 |
| C10:2 | 1.78 (0.49-6.42) | 0.378 | 17.33 (0.08-3942.22) | 0.303 |
| C8:1-DC | 1.43 (0.44-4.63) | 0.549 | 0.09 (0-9.78) | 0.309 |
| C7-DC | 1.94 (0.51-7.44) | 0.334 | 0.08 (0-13.09) | 0.331 |
| C14:1 | 1.44 (0.56-3.69) | 0.453 | 0.24 (0.01-4.39) | 0.336 |
| C5:1 | 2.4 (0.3-18.87) | 0.406 | 0.01 (0-155.33) | 0.358 |
| C18:2 | 0.69 (0.22-2.13) | 0.518 | 2.32 (0.32-16.56) | 0.401 |
| C4-OH | 1.58 (0.48-5.19) | 0.451 | 0.27 (0.01-6.33) | 0.415 |
| C10 | 1.65 (0.39-6.97) | 0.495 | 0.44 (0.05-3.93) | 0.465 |
| C14:2 | 1.34 (0.52-3.44) | 0.548 | 0.39 (0.02-6.47) | 0.511 |
| C18:2-OH | 0.62 (0.2-1.92) | 0.412 | 10.61 (0-63385.27) | 0.595 |
| C16:2 | 1.6 (0.62-4.16) | 0.331 | 0.51 (0.01-31.61) | 0.747 |
| C5 | 2.13 (0.48-9.58) | 0.322 | 0.9 (0.12-7.01) | 0.919 |
| C8:1 | 1.48 (0.46-4.74) | 0.506 | 1.02 (0.35-2.92) | 0.976 |
| C14 | 1.36 (0.37-4.95) | 0.644 | 0.12 (0-10.3) | 0.346 |
| C20:4 | 1.38 (0.33-5.75) | 0.654 | 0.34 (0-125.41) | 0.720 |
| C10:3 | 1.35 (0.37-4.93) | 0.649 | 1.33 (0.07-25.91) | 0.849 |
| C6 | 1.33 (0.31-5.71) | 0.704 | 0.13 (0-13.83) | 0.393 |
| C12-OH/C10-DC | 1.21 (0.41-3.58) | 0.728 | 0.17 (0-13.95) | 0.434 |
| C5-DC | 1.2 (0.42-3.46) | 0.738 | 0.79 (0.01-44.82) | 0.909 |
| C18:1-OH/C16:1-DC | 1.19 (0.34-4.15) | 0.782 | 0 (0-0.18) | 0.016 |
| C8:1-OH/C6:1-DC | 1.13 (0.35-3.67) | 0.840 | 18.97 (0.11-3410.32) | 0.267 |
| C16:1 | 1.08 (0.43-2.71) | 0.876 | 0.1 (0-5.58) | 0.264 |
| C4/Ci4 | 1.07 (0.37-3.05) | 0.901 | 1.34 (0.2-9.14) | 0.767 |
| C12:1 | 0.98 (0.24-3.98) | 0.973 | 0.13 (0.01-3.26) | 0.217 |
| C18:1 | 0.97 (0.34-2.76) | 0.953 | 1.17 (0.3-4.5) | 0.821 |
| C10:1 | 1 (0.19-5.29) | 1.000 | 24.44 (0.57-1056.92) | 0.096 |

Table S13: Pasireotide group metabolomics correlation to aGVHD(Conventional-C, Amino Acid-AA, Acylcarnitines-AC)

| Marker (C) | OR-Base (95% CI) | P-Base | OR-Ratio (95% CI) | P-Ratio |
| --- | --- | --- | --- | --- |
| HBUT | 1.61 (0.66-3.89) | 0.295 | 2.27 (0.2-25.44) | 0.507 |
| NEFA | 1.86 (0.53-6.49) | 0.333 | 0.65 (0.3-1.42) | 0.283 |
| Glycerol | 1.49 (0.64-3.45) | 0.350 | 1.14 (0.69-1.86) | 0.615 |
| TG-B | 0.4 (0.05-3.17) | 0.386 | 0.4 (0-2364316.26) | 0.908 |
| LACT | 0.67 (0.15-3.03) | 0.606 | 0.7 (0.34-1.41) | 0.313 |

| Marker (AA) | OR-Base (95% CI) | P-Base | OR-Ratio (95% CI) | P-Ratio |
| --- | --- | --- | --- | --- |
| Tyr | 253.46 (0.31-206190.07) | 0.105 | 1486497023074482688 (21.26-1.0394436787742e+35) | 0.034 |
| His | 0 (0-1.12) | 0.055 | 0 (0-74.19) | 0.100 |
| Val | 0.05 (0-2.78) | 0.142 | 0 (0-3655.6) | 0.174 |
| Leu/Ile | 0.06 (0-4.66) | 0.202 | 0.02 (0-643005611.08) | 0.750 |
| Asx | 7.2 (0.11-483.09) | 0.357 | 979941.54 (2.46-389895098928.46) | 0.036 |
| Ser | 9.95 (0.11-902.5) | 0.318 | 331451177.97 (0-60313959779382902784) | 0.138 |
| Glx | 0.96 (0.05-20.16) | 0.977 | 872855885055.57 (0-1.34957524188597e+27) | 0.123 |
| Ala | 0.98 (0.03-28.6) | 0.991 | 0 (0-127145.41) | 0.222 |
| Phe | 0.5 (0.02-15.27) | 0.688 | 0.01 (0-13.08) | 0.217 |
| Orn | 0.44 (0.01-19.99) | 0.672 | 2835.81 (0-50010652207.45) | 0.350 |
| Pro | 1.63 (0.07-39.17) | 0.764 | 1231.51 (0-87978000801.53) | 0.441 |
| Met | 1.2 (0.09-16.54) | 0.893 | 0.07 (0-104.08) | 0.473 |
| Gly | 1.75 (0.08-39.24) | 0.723 | 103.31 (0-11039998747447) | 0.720 |
| Cit | 1.8 (0.29-10.97) | 0.526 | 3.1 (0-3280.86) | 0.750 |
| Arg | 1.01 (0.23-4.47) | 0.990 | 0.44 (0-329.99) | 0.806 |

| Marker (AC) | OR-Base (95% CI) | P-Base | OR-Ratio (95% CI) | P-Ratio |
| --- | --- | --- | --- | --- |
| C16:1 | 2.99 (0.66-13.53) | 0.155 | 0.02 (0-2.34) | 0.105 |
| C16 | 4.68 (0.57-38.43) | 0.150 | 0.04 (0-1.43) | 0.079 |
| C18:1-OH/C16:1-DC | 3.84 (0.36-41.21) | 0.267 | 0 (0-44.13) | 0.120 |
| C18-OH/C16-DC | 3.36 (0.66-16.99) | 0.143 | 0 (0-5.69) | 0.099 |
| C18:1-DC | 1.87 (0.39-8.99) | 0.435 | 0 (0-1.75) | 0.064 |
| C20-OH/C18-DC | 2.54 (0.6-10.76) | 0.206 | 0 (0-5.48) | 0.104 |
| C22 | 15.3 (0.8-293.23) | 0.070 | 0 (0-2.71) | 0.061 |
| C5 | 0.55 (0.1-3) | 0.489 | 10.59 (0.21-535.2) | 0.238 |
| C5-DC | 0.44 (0.07-2.86) | 0.393 | 157.05 (0.08-307901.99) | 0.191 |
| C7-DC | 2.25 (0.39-12.91) | 0.364 | 0.01 (0-22.86) | 0.261 |
| C12:1 | 1.95 (0.37-10.23) | 0.431 | 0.1 (0-5.72) | 0.267 |
| C12 | 2.06 (0.45-9.41) | 0.353 | 0.12 (0-5.39) | 0.274 |
| C14 | 4.04 (0.55-29.5) | 0.169 | 0.02 (0-5.44) | 0.174 |
| C14-OH/C12-DC | 2.83 (0.34-23.57) | 0.337 | 0.01 (0-37.32) | 0.285 |
| C16:1-OH/C14:1-DC | 2.74 (0.55-13.61) | 0.217 | 0 (0-13.44) | 0.164 |
| C18 | 4.07 (0.41-40.38) | 0.231 | 0.02 (0-9.5) | 0.215 |
| C14:1 | 2.27 (0.62-8.31) | 0.217 | 0.17 (0.01-5.21) | 0.314 |
| C8 | 2.26 (0.49-10.48) | 0.299 | 0.14 (0-7.71) | 0.337 |
| C10-OH/C8-DC | 3.4 (0.63-18.28) | 0.153 | 0.09 (0-25.33) | 0.399 |
| C16:2 | 2.71 (0.73-10.07) | 0.138 | 0.1 (0-22.45) | 0.407 |
| C18:1 | 1.58 (0.42-5.87) | 0.497 | 0.58 (0.16-2.18) | 0.424 |
| C2 | 3.6 (0.44-29.74) | 0.234 | 2 (0.23-17.67) | 0.533 |
| C3 | 0.53 (0.09-3.14) | 0.484 | 0.51 (0.08-3.17) | 0.470 |
| C4/Ci4 | 0.59 (0.13-2.56) | 0.479 | 2.61 (0.13-50.92) | 0.527 |
| C14:1-OH | 3.27 (0.6-17.7) | 0.169 | 0.1 (0-109.16) | 0.519 |
| C5:1 | 0.22 (0.01-5) | 0.344 | 11.56 (0-13756480.04) | 0.732 |
| C10 | 2.12 (0.41-10.95) | 0.372 | 0.54 (0.02-11.7) | 0.695 |
| C12-OH/C10-DC | 1.79 (0.42-7.52) | 0.429 | 0.5 (0-178.76) | 0.817 |
| C14:2 | 1.63 (0.53-5.01) | 0.396 | 0.52 (0.02-16.21) | 0.708 |
| C16-OH/C14-DC | 2.34 (0.34-15.95) | 0.384 | 0.37 (0-3417.42) | 0.833 |
| C20 | 2.12 (0.33-13.57) | 0.427 | 0.15 (0-16802.18) | 0.750 |
| C5-OH/C3-DC | 0.37 (0.05-2.92) | 0.345 | 0.57 (0-1007.36) | 0.884 |
| C6-DC/C8-OH | 1.64 (0.27-10.01) | 0.593 | 0.23 (0-35.05) | 0.566 |
| C6 | 1.52 (0.16-14.8) | 0.721 | 0.12 (0-88.51) | 0.533 |
| C4-OH | 0.78 (0.14-4.36) | 0.774 | 5.9 (0.08-454.31) | 0.423 |
| C4-DC/Ci4-DC | 1.36 (0.18-10.43) | 0.765 | 1.23 (0-5702.69) | 0.961 |
| C8:1-OH/C6:1-DC | 0.91 (0.19-4.37) | 0.907 | 1133.49 (0.14-9203066.89) | 0.126 |
| C10:2 | 1.08 (0.2-5.92) | 0.925 | 8.16 (0.01-8594.41) | 0.554 |
| C8:1 | 0.88 (0.18-4.44) | 0.881 | 0.76 (0.14-4.05) | 0.748 |
| C10:3 | 0.88 (0.17-4.64) | 0.876 | 0.62 (0.01-39.3) | 0.820 |
| C18:2 | 1.09 (0.26-4.62) | 0.905 | 0.75 (0.08-6.72) | 0.797 |
| C20:4 | 1.15 (0.14-9.26) | 0.894 | 0.32 (0-2274.98) | 0.801 |
| C10:1 | 1 (0.19-5.23) | 0.998 | 23.49 (0.13-4237.72) | 0.234 |
| C18:2-OH | 0.97 (0.14-6.52) | 0.975 | 39.15 (0-2725052.65) | 0.519 |
| C8:1-DC | 1.02 (0.19-5.41) | 0.980 | 0.46 (0-558.13) | 0.829 |

Table S14: Pasireotide group metabolomics correlation to cGVHD(Conventional-C, Amino Acid-AA, Acylcarnitines-AC)

| Marker (C) | OR-Base (95% CI) | P-Base | OR-Ratio (95% CI) | P-Ratio |
| --- | --- | --- | --- | --- |
| LACT | 2.14 (0.45-10.17) | 0.338 | 1.31 (0.73-2.33) | 0.362 |
| NEFA | 1.64 (0.52-5.16) | 0.396 | 0.7 (0.34-1.42) | 0.321 |
| HBUT | 1.51 (0.64-3.56) | 0.346 | 0.9 (0.1-8.08) | 0.928 |
| TG-B | 1.75 (0.28-11) | 0.552 | 4.97 (0-11685595.37) | 0.830 |
| Glycerol | 1.07 (0.48-2.35) | 0.871 | 1.19 (0.75-1.89) | 0.466 |

| Marker (AA) | OR-Base (95% CI) | P-Base | OR-Ratio (95% CI) | P-Ratio |
| --- | --- | --- | --- | --- |
| Gly | 0.04 (0-11.87) | 0.268 | 0 (0-27.39) | 0.070 |
| His | 0 (0-1.23) | 0.059 | 0 (0-19.46) | 0.079 |
| Tyr | 7.28 (0.13-402.83) | 0.333 | 366119.22 (0.06-2403985237438.87) | 0.110 |
| Asx | 7.04 (0.12-405.37) | 0.345 | 1191.82 (0.06-22962266.62) | 0.159 |
| Arg | 0.22 (0.03-1.53) | 0.125 | 0 (0-9.98) | 0.145 |
| Cit | 4.28 (0.56-32.5) | 0.160 | 0.05 (0-74.52) | 0.419 |
| Glx | 7.46 (0.18-309.04) | 0.290 | 0.07 (0-8624907532.83) | 0.841 |
| Val | 0.26 (0.01-7.29) | 0.429 | 0 (0-173.16) | 0.115 |
| Orn | 3.33 (0.1-106.15) | 0.496 | 0.01 (0-13948.62) | 0.500 |
| Leu/Ile | 0.35 (0.01-16.2) | 0.588 | 0.03 (0-433409714.38) | 0.771 |
| Ser | 2.52 (0.06-110.36) | 0.632 | 0.45 (0-440586478.57) | 0.940 |
| Met | 1.18 (0.07-19.48) | 0.909 | 0 (0-0.74) | 0.044 |
| Ala | 0.73 (0.03-18.69) | 0.849 | 0 (0-9468609.6) | 0.404 |
| Phe | 0.87 (0.03-23.44) | 0.936 | 0.05 (0-33.79) | 0.368 |
| Pro | 0.68 (0.03-14.56) | 0.802 | 0.25 (0-5765594.86) | 0.873 |

| Marker (AC) | OR-Base (95% CI) | P-Base | OR-Ratio (95% CI) | P-Ratio |
| --- | --- | --- | --- | --- |
| C6 | 5.44 (0.38-77.34) | 0.211 | 0.02 (0-35.41) | 0.311 |
| C8 | 7.16 (0.96-53.63) | 0.055 | 0.06 (0-5.42) | 0.218 |
| C10 | 5.93 (0.88-40.17) | 0.068 | 0.06 (0-2.29) | 0.130 |
| C12:1 | 3.63 (0.64-20.69) | 0.146 | 0.06 (0-4.37) | 0.197 |
| C14 | 3.72 (0.56-24.7) | 0.173 | 0.06 (0-11.34) | 0.291 |
| C16:1 | 4.58 (0.94-22.39) | 0.060 | 0.02 (0-3.13) | 0.132 |
| C16 | 6.87 (0.83-56.61) | 0.073 | 0.13 (0.01-3.36) | 0.221 |
| C16-OH/C14-DC | 4.81 (0.58-39.99) | 0.146 | 0.01 (0-70.14) | 0.290 |
| C18:2 | 44.15 (1.84-1059.17) | 0.019 | 0.02 (0-0.77) | 0.037 |
| C18:1 | 19.7 (1.65-235.81) | 0.019 | 0.1 (0.01-1.05) | 0.055 |
| C18 | 8.51 (0.71-102.07) | 0.091 | 0.05 (0-21.85) | 0.332 |
| C18-OH/C16-DC | 3.23 (0.68-15.29) | 0.140 | 0 (0-46.11) | 0.239 |
| C20:4 | 4.93 (0.47-52.24) | 0.185 | 0.01 (0-93.1) | 0.297 |
| C20 | 3.86 (0.53-28.08) | 0.182 | 0 (0-324.95) | 0.306 |
| C18:1-DC | 3.56 (0.61-20.67) | 0.157 | 0 (0-10.91) | 0.154 |
| C22 | 0.21 (0.02-1.87) | 0.163 | 108976.03 (0.01-2364250027032.25) | 0.178 |
| C10:3 | 3.24 (0.5-20.88) | 0.216 | 0.13 (0-14.74) | 0.396 |
| C10-OH/C8-DC | 2.82 (0.57-13.97) | 0.205 | 0.09 (0-23.25) | 0.401 |
| C14:2 | 2.35 (0.72-7.6) | 0.155 | 0.29 (0.01-9.64) | 0.490 |
| C16:2 | 2.43 (0.69-8.55) | 0.168 | 0.17 (0-29.85) | 0.498 |
| C2 | 4.98 (0.57-43.49) | 0.146 | 1.24 (0.14-10.79) | 0.844 |
| C4-OH | 6.28 (0.86-45.68) | 0.069 | 0.37 (0.01-25.69) | 0.649 |
| C8:1-OH/C6:1-DC | 3 (0.52-17.23) | 0.219 | 3.37 (0-14773.31) | 0.776 |
| C14-OH/C12-DC | 5 (0.64-38.88) | 0.124 | 0.2 (0-131.88) | 0.629 |
| C16:1-OH/C14:1-DC | 3.22 (0.65-16) | 0.152 | 0.16 (0-660.21) | 0.667 |
| C12-OH/C10-DC | 2.45 (0.56-10.59) | 0.232 | 0.05 (0-18.24) | 0.319 |
| C7-DC | 3.08 (0.46-20.67) | 0.246 | 0.26 (0-288.91) | 0.707 |
| C14:1 | 2.01 (0.58-6.96) | 0.272 | 0.21 (0.01-5.76) | 0.356 |
| C4-DC/Ci4-DC | 3.42 (0.4-29.57) | 0.263 | 52.96 (0-1265838.3) | 0.440 |
| C10:1 | 2.79 (0.42-18.68) | 0.289 | 3.5 (0.04-312.94) | 0.585 |
| C8:1 | 2.63 (0.39-17.87) | 0.322 | 2.06 (0.2-20.81) | 0.540 |
| C20-OH/C18-DC | 1.96 (0.51-7.44) | 0.324 | 0.07 (0-201.95) | 0.512 |
| C5-OH/C3-DC | 0.39 (0.05-2.82) | 0.351 | 0.25 (0-433.03) | 0.716 |
| C12 | 2.01 (0.44-9.2) | 0.367 | 0.06 (0-2.92) | 0.154 |
| C18:2-OH | 2.51 (0.33-19.12) | 0.374 | 0.45 (0-5927.07) | 0.868 |
| C14:1-OH | 1.96 (0.42-9.18) | 0.394 | 1.07 (0-681.8) | 0.983 |
| C10:2 | 1.96 (0.34-11.18) | 0.450 | 0.36 (0-244.99) | 0.759 |
| C6-DC/C8-OH | 1.88 (0.31-11.47) | 0.492 | 1.15 (0.01-156.96) | 0.955 |
| C5:1 | 2.48 (0.12-50) | 0.554 | 4.57 (0-5756896.09) | 0.832 |
| C8:1-DC | 1.51 (0.29-7.84) | 0.625 | 0.39 (0-422.53) | 0.793 |
| C5-DC | 0.69 (0.13-3.73) | 0.668 | 1.88 (0-1188.63) | 0.847 |
| C4/Ci4 | 1.17 (0.28-4.9) | 0.828 | 9.38 (0.31-283.93) | 0.198 |
| C5 | 1.15 (0.21-6.17) | 0.872 | 0.36 (0.01-8.99) | 0.536 |
| C18:1-OH/C16:1-DC | 1.08 (0.17-6.67) | 0.938 | 0.25 (0-39474.54) | 0.820 |
| C3 | 1.02 (0.23-4.41) | 0.984 | 4.92 (0.42-57.07) | 0.203 |

Table S15: Pasireotide group metabolomics correlation to TRM(Conventional-C, Amino Acid-AA, Acylcarnitines-AC)

| Marker (C) | OR-Base (95% CI) | P-Base | OR-Ratio (95% CI) | P-Ratio |
| --- | --- | --- | --- | --- |
| LACT | 2.78 (0.46-16.67) | 0.263 | 1.28 (0.6-2.74) | 0.519 |
| TG-B | 4.69 (0.2-111.52) | 0.339 | 1093801.06 (0-2841801654704828) | 0.209 |
| Glycerol | 0.58 (0.19-1.77) | 0.339 | 1.19 (0.6-2.35) | 0.613 |
| HBUT | 1.11 (0.41-2.99) | 0.837 | 1.2 (0.04-34.91) | 0.916 |
| NEFA | 0.81 (0.21-3.05) | 0.750 | 1.07 (0.64-1.8) | 0.799 |

| Marker (AA) | OR-Base (95% CI) | P-Base | OR-Ratio (95% CI) | P-Ratio |
| --- | --- | --- | --- | --- |
| Ser | 0.12 (0-143.23) | 0.560 | 0 (0-10455.45) | 0.140 |
| Val | 89.35 (0.01-1345381.01) | 0.360 | 0 (0-390672.02) | 0.128 |
| His | 0 (0-455.74) | 0.294 | 0 (0-16412759.34) | 0.162 |
| Asx | 8811.48 (0.11-697677535.62) | 0.114 | 3523093651.56 (0-8.76866883511749e+22) | 0.162 |
| Glx | 15.13 (0.24-962.87) | 0.200 | 3251930556489.73 (0-7.04015292359739e+29) | 0.157 |
| Ala | 2.28 (0.02-228.65) | 0.725 | 1523149.24 (0-1.82025398938598e+21) | 0.422 |
| Leu/Ile | 6.72 (0-323266.17) | 0.729 | 0 (0-7.35133295073179e+23) | 0.304 |
| Arg | 2.15 (0.21-22.51) | 0.522 | 107.11 (0-3784564.8) | 0.382 |
| Gly | 1.64 (0.05-57.45) | 0.784 | 0.13 (0-1175892067178.41) | 0.893 |
| Met | 1.52 (0.06-41.76) | 0.805 | 1.49 (0-17737.18) | 0.933 |
| Tyr | 0.36 (0-84.21) | 0.711 | 6.74 (0-1815539990.3) | 0.847 |
| Orn | 2.37 (0.02-290.3) | 0.725 | 0.19 (0-19853868.37) | 0.861 |
| Cit | 2.44 (0.17-35.04) | 0.511 | 7.66 (0-123607.99) | 0.681 |
| Pro | 0.94 (0.01-66.78) | 0.979 | 23542.47 (0-648817059198490) | 0.412 |
| Phe | NA | NA | NA | NA |

| Marker (AC) | OR-Base (95% CI) | P-Base | OR-Ratio (95% CI) | P-Ratio |
| --- | --- | --- | --- | --- |
| C4-OH | 31.08 (0.17-5587.07) | 0.194 | 0 (0-63.06) | 0.168 |
| C6 | 18.3 (0.55-612.85) | 0.105 | 0 (0-12.29) | 0.149 |
| C8 | 3897.43 (0-12074601374.56) | 0.278 | 0 (0-57597.66) | 0.235 |
| C6-DC/C8-OH | 93 (0.08-103502.9) | 0.205 | 0 (0-11.9) | 0.102 |
| C10:1 | 9.03 (0.33-243.87) | 0.191 | 0.01 (0-16.66) | 0.235 |
| C10 | 59.34 (0.17-20912) | 0.172 | 0 (0-8.76) | 0.121 |
| C8:1-DC | 18.79 (0.42-834.93) | 0.130 | 0 (0-13.16) | 0.103 |
| C12 | 3719.72 (0.01-1007653905.51) | 0.198 | 0 (0-312.3) | 0.165 |
| C14:2 | 41.58 (0.14-12165.9) | 0.198 | 0 (0-118.66) | 0.155 |
| C14:1 | 33.12 (0.34-3184.42) | 0.133 | 0 (0-2797.8) | 0.235 |
| C14 | 23.08 (0.45-1195.31) | 0.119 | 0 (0-130.28) | 0.206 |
| C16:2 | 112.57 (0.36-34842.16) | 0.106 | 0 (0-1313.36) | 0.100 |
| C16:1 | 15.6 (0.5-482.56) | 0.117 | 0 (0-77.81) | 0.150 |
| C16:1-OH/C14:1-DC | 20.23 (0.12-3430.12) | 0.251 | 0 (0-53817.25) | 0.219 |
| C16-OH/C14-DC | 32.01 (0.06-17505.65) | 0.281 | 0 (0-22375.48) | 0.173 |
| C18:1 | 2161.26 (0.08-55496796.57) | 0.138 | 0 (0-23.7) | 0.168 |
| C18:1-OH/C16:1-DC | 34.05 (0.59-1972.79) | 0.089 | 0 (0-33.7) | 0.075 |
| C20-OH/C18-DC | 4.72 (0.3-73.7) | 0.269 | 0 (0-1167.32) | 0.229 |
| C16 | 424966215.08 (0-1.44308154244845e+24) | 0.276 | 0 (0-39274665205.92) | 0.268 |
| C18:2 | 15.75 (0.17-1463.55) | 0.233 | 0.01 (0-34.96) | 0.285 |
| C20:4 | 12.7 (0.18-912.73) | 0.244 | 0 (0-10477.45) | 0.272 |
| C3 | 6.53 (0.41-104.86) | 0.185 | 0.17 (0-5.47) | 0.313 |
| C10:2 | 10.57 (0.41-271.8) | 0.155 | 0.01 (0-281.98) | 0.382 |
| C8:1 | 6.14 (0.43-87.67) | 0.181 | 0.38 (0.04-3.6) | 0.401 |
| C5 | 15.39 (0.25-933.77) | 0.192 | 0.5 (0.01-35.7) | 0.750 |
| C4/Ci4 | 5.27 (0.23-118.57) | 0.296 | 65.24 (0.09-45184.26) | 0.211 |
| C18-OH/C16-DC | 3.43 (0.28-42.05) | 0.335 | 0 (0-1670.77) | 0.242 |
| C12-OH/C10-DC | 389.63 (0-49846687.66) | 0.320 | 0 (0-30766734451218180) | 0.305 |
| C5-OH/C3-DC | 4.97 (0.19-127.13) | 0.333 | 0.13 (0-1261.52) | 0.665 |
| C4-DC/Ci4-DC | 3.58 (0.2-62.56) | 0.383 | 0 (0-342.32) | 0.226 |
| C10:3 | 3 (0.29-30.72) | 0.355 | 0.01 (0-7.25) | 0.158 |
| C20 | 3.14 (0.27-36.46) | 0.359 | 0 (0-2335.38) | 0.282 |
| C2 | 3.42 (0.23-51.86) | 0.376 | 6.08 (0.13-274.74) | 0.353 |
| C18:1-DC | 2.16 (0.32-14.58) | 0.431 | 0 (0-178.58) | 0.218 |
| C7-DC | 2.84 (0.08-102.14) | 0.567 | 0 (0-103.27) | 0.147 |
| C5:1 | 0.27 (0-25.55) | 0.574 | 1.76 (0-861926947.23) | 0.956 |
| C8:1-OH/C6:1-DC | 1.68 (0.22-12.77) | 0.614 | 0 (0-173.52) | 0.320 |
| C22 | 1.49 (0.17-13.36) | 0.723 | 0 (0-622228.1) | 0.568 |
| C5-DC | 1.14 (0.12-10.77) | 0.908 | 0.87 (0-4604.41) | 0.974 |
| C18:2-OH | 1.13 (0.01-98.63) | 0.958 | 0 (0-934421159.61) | 0.489 |
| C10-OH/C8-DC | NA | NA | NA | NA |
| C12:1 | NA | NA | NA | NA |
| C14:1-OH | NA | NA | NA | NA |
| C14-OH/C12-DC | NA | NA | NA | NA |
| C18 | NA | NA | NA | NA |
