# Supplementary material for: A phase 2 trial of the somatostatin analog pasireotide to prevent GI toxicity and acute GVHD in allogeneic hematopoietic stem cell transplant
Source: PLoS One. 2021 Jun 25;16(6):e0252995. doi: 10.1371/journal.pone.0252995 (PMC8232534; doi:10.1371/journal.pone.0252995)
Supplement: S4 File — (PDF) [file pone.0252995.s004.pdf]

A Phase II Trial for the Prevention of Gastrointestinal Toxicity from Total Body Irradiation or High Dose Chemotherapy with Pasireotide

Principal Investigator:

Anthony Sung, MD
Duke University Medical Center
DUMC Box 3961
Durham, NC 27710
Phone: 919-668-5710
Fax: 919-668-1091

Co-Investigators:

Nelson Chao, MD, MBA
Cristina Gasparetto, MD
Mitchell Horwitz, MD
Gwynn Long, MD
Ashley Morris Engemann, PharmD
David Rizzieri, MD
Stefanie Sarantopoulus, MD, PhD
Keith Sullivan, MD
Richard Lopez, MD
Daniel Wild, MD

Study Statistician:
Zhiguo Li, Ph.D.

Duke IRB Protocol: 00051736

ClinicalTrials.gov Identifier: NCT02215070

TABLE OF CONTENTS

1 ABBREVIATIONS 5

2 OBJECTIVES 5
2.1 Primary Objective: 5
2.2 Secondary Objectives: 5
2.3 Exploratory Objectives: 5

3 ENDPOINTS 6
3.1 Primary Endpoint 6
3.2 Secondary Endpoints 6
3.3 Exploratory Endpoints 6

4 BACKGROUND AND RATIONALE FOR USE OF PASIREOTIDE 6

5 DESCRIPTION OF PASIREOTIDE 8
5.1 Mechanism of Action 9
5.2 Pharmacodynamics 10
5.3 Pharmacokinetics 10
5.4 Absorption and Distribution 10
5.5 Metabolism and Excretion 11
5.6 Steady-state pharmacokinetics 11
5.7 Special Populations 11
5.8 Hepatic impairment 11
5.9 Renal impairment 12
5.10 Pediatric Patients 12
5.11 Geriatric patients 12
5.12 Drug Interaction Studies: 12

6 NONCLINICAL TOXICOLOGY 12
6.1 Carcinogenesis 12
6.2 Mutagenesis 12
6.3 Impairment of Fertility 12

7 CLINICAL STUDIES 13
7.1 Clinical Studies in Children 15
7.2 Geriatric Use 15

8 STUDY DRUG INFORMATION 15
8.1 Dosage 15
8.2 Renal or hepatic insufficiency: 15
8.3 Storage and Handling 16
8.4 Adverse Events 16
8.5 Drug Interactions 19
8.5.1 Effects of Other Drugs on Pasireotide 19

8.5.2 Effects of Pasireotide on Other Drugs 19
8.6 Contraindications 19
8.7 Warnings 20
8.7.1 Hypocortisolism 20
8.7.2 Hyperglycemia and Diabetes 20
8.7.3 Bradycardia and QT Prolongation 21
8.7.4 Liver Test Elevations 21
8.7.5 Cholelithiasis 22
8.7.6 Monitoring for Deficiency of Pituitary Hormones 22

9 PATIENT ELIGIBILITY 22
9.1 Inclusion Criteria 22
9.2 Exclusion Criteria 22
9.3 Patient Numbering 24

10 MATERIAL AND DATA 24

11 TREATMENT PLAN AND MODIFICATIONS 25
11.1 Study Design: 26
11.2 Video Capsule Endoscopy 26
11.3 Biomarker Assays 27
11.3.1 Citrulline assay 27
11.3.2 Calprotectin assay 27
11.4 Dose Modifications and Treatment Interruptions/Discontinuation. 28
11.5 Study Withdrawal 29

12 ANTICIPATED TOXICITIES 29
12.1 Myelosuppression: 30
12.2 Gastrointestinal: 30
12.3 Hyperglycemia 30
12.4 Cardiac: 30
12.5 Pulmonary: 30
12.6 Hepatic: 31
12.7 Renal: 31
12.8 GVHD: 31
12.9 Neurologic: 31
12.10 Miscellaneous: 31

13 DATA MANAGEMENT AND DATA SAFETY MONITORING 32
13.1 Data Management 32
13.1.1 Electronic Case Report Forms (eCRFs) 32
13.1.2 Data Management Procedures and Data Verification 32
13.1.3 Study Closure 33
13.2 Data Safety Monitoring 33

14 SAFETY AND STUDY DATA MONITORING 33
14.1 Safety 33
14.1.1 Adverse Event Reporting. 33
14.1.2 SAE Reporting 35

14.1.3 Pregnancy 36
14.2 Data Monitoring: 37

15 STATISTICAL CONSIDERATIONS 37
15.1 Objectives 38
15.1.1 Primary Objective: 38
15.1.2 Secondary Objectives: 38
15.2 Exploratory Objectives: 38
15.3 Endpoints: 38
15.3.1 Primary Endpoint 38
15.3.2 Secondary Endpoints 39
15.3.3 Exploratory Endpoints 39
15.4 Stopping Rules 39

16 REFERENCES 41

APPENDIX A: STUDY CALENDAR FOR SPECIAL STUDIES 43

APPENDIX B: CAPSULE ENDOSCOPY PREPARATION INSTRUCTIONS 45

APPENDIX C: GUIDANCE FOR HYPERGLYCEMIA MANAGEMENT 47

APPENDIX D: QT PROLONGING MEDICATIONS (INEXHAUSTIVE) 48

APPENDIX E: STUDY DRUGS AND EXAMPLE PREPARATORY REGIMENS 50
E1. Total Body Irradiation 50
E2. Busulfan (Myerlan) NSC #750 50
E3. Cyclophosphamide (Cytoxan, CTX) NSC #26271 50
E4. Cyclosporine (Sandimmune) 51
E5. Methotrexate (MTX) 52
E6. Example Schedule 54

APPENDIX F: ACUTE GVHD SCORING 55

APPENDIX G: CHRONIC GVHD SCORING 56

APPENDIX H: SAE COVERSHEETS…………………………..………………………60

1 ABBREVIATIONS

CTX	Cytoxan
G-CSF	Granulocyte colony stimulating factor
GI	Gastrointestinal
GVHD	Graft vs Host Disease
KGF	keratinocyte growth factor
MTX	methotrexate
sc	subcutaneous
SCT	Stem Cell Transplantation
TBI	Total Body Irradiation
VCE	Video Capsule Endoscopy

2 OBJECTIVES

2.1 Primary Objective:
To evaluate the efficacy and safety of pasireotide in mitigating GI toxicity from the preparatory regimens for allogeneic stem cell transplantation.

2.2 Secondary Objectives:
To evaluate the efficacy of pasireotide in reducing the incidence and severity of acute GVHD at 100 days post-transplant compared to historical controls.

To evaluate the efficacy of pasireotide in reducing the incidence and severity of chronic GVHD at 1 year post-transplant.

To compare the rate of overall survival in patients treated with pasireotide at 1 year post-transplant to historical controls.

To compare the rate of disease free survival in patients treated with pasireotide at 1 year post-transplant to historical controls.

Historical controls will be drawn from a group of patients matched for age, disease, graft source, preparatory regimen, GVHD prophylaxis, and number of prior therapies but who will not receive pasireotide.

2.3 Exploratory Objectives:
To evaluate plasma citrulline and fecal calprotectin levels as biomarkers of GI tract health and function in SCT patients.

To evaluate GI toxicity assessments by video capsule endoscopy.

3 ENDPOINTS

3.1 Primary Endpoint

The incidence of Grades 3-4 Gastrointestinal toxicity in SCT patients who are treated with pasireotide, based on CTCAE 4.0 criteria, through transplant day 30.

3.2 Secondary Endpoints

The secondary endpoints will be:

- Incidence and maximum severity of Acute GVHD at 100 days post-transplant.
- Incidence and maximum severity of Chronic GVHD at 1 year post-transplant.
- Rate of overall survival at 1 year post-transplant.
- Rate of Disease free survival at 1 year post-transplant.

3.3 Exploratory Endpoints

As exploratory endpoints, citrulline levels as well as fecal calprotectin will be measured at baseline, transplant Day 0 and at weekly intervals for the first two weeks post-transplant. These results will be correlated with other signs and symptoms of GI toxicity for the first two weeks post-transplant.

GI toxicity will also be assessed by use of video capsule endoscopy in a subset of 10 patients and five controls. Images will be scored by an experienced endoscopist (Dr.Wild).

4 BACKGROUND AND RATIONALE FOR USE OF PASIREOTIDE

Total body irradiation (TBI) or high dose busulfan is a standard preparatory regimen for the treatment of hematological disease prior to stem cell transplantation. Unfortunately, these approaches are associated with significant acute and chronic toxicities, and injuries to the bone marrow and gastrointestinal (GI) tract are the main determinants of lethality after total-body irradiation (TBI) [1, 2]. Specifically, the use of this modality results in acute gastrointestinal toxicity such as nausea, vomiting and diarrhea. The inflammatory response is also likely important in setting up the appropriate milieu for the induction of graft versus host disease (GVHD). Some progress has been made in the management of radiation injury of the hematopoietic/immune system. However, effective and safe countermeasures against structural injury and dysfunction in the GI tract remain an unmet need [2].

In the intestine, radiation damages crypt stem cells and proliferating transit cells, thereby leading to disruption of the epithelial barrier that normally protects the subepithelial matrix from the influence of the digestive enzymes that are normally present in the intestinal lumen [3]. Among the various intraluminal factors, pancreatic enzymes exert a particularly prominent influence on development of intestinal radiation toxicity [4]. Hence controlling pancreatic enzyme secretion has been explored as a

method to attenuate acute mucosal injury and increases survival after abdominal irradiation [5, 6].

Efforts to counter the GI toxicity have included drugs such as keratinocyte growth factor (KGF) that has been shown to decrease mucositis associated with TBI. Unfortunately, the data with KGF is relatively marginal and this drug has not been universally accepted into routine clinical use. Somatostatin analogs are known as inhibitors of growth hormone (GH) and other hormones (including gastro-intestinal hormones) and, among many different activities that influence the GI tract, inhibit the secretion of pancreatic enzymes into the bowel lumen [7, 8]. Earlier studies showed that the “prototype” somatostatin analog, octreotide, markedly ameliorated mucosal injury in the small bowel after localized irradiation [9]. Subsequent randomized clinical trials have translated this to patients with severe radiation-induced GI toxicity, thus confirming therapeutic efficacy in humans [10].

While octreotide can be used in the clinical situation to reduce side effects of radiation therapy, its short half-life is a serious limitation in situations where intravenous or multiple subcutaneous (s.c.) administrations are not logistically feasible. A more recently developed synthetic somatostatin analog, SOM230 (pasireotide, Pasireotide), however, has much greater metabolic stability and thus circumvents this obstacle [11]. It was also reported in 2009 that SOM230 administered prior to or 4 h after TBI in a mouse model conferred a highly statistically significant survival benefit, even though we also demonstrated that SOM230 did not appear to have direct cytoprotective effects on the bone marrow or the intestine [12]. Hence, while post-TBI intestinal crypt survival and hematopoietic injury were similar, the intestinal barrier was structurally and functionally preserved. Moreover, protection was reversed by co-administration of pancreatic enzymes, thus strongly suggesting that reduced secretion of proteolytic enzymes into the bowel lumen was the mechanism of action [12].

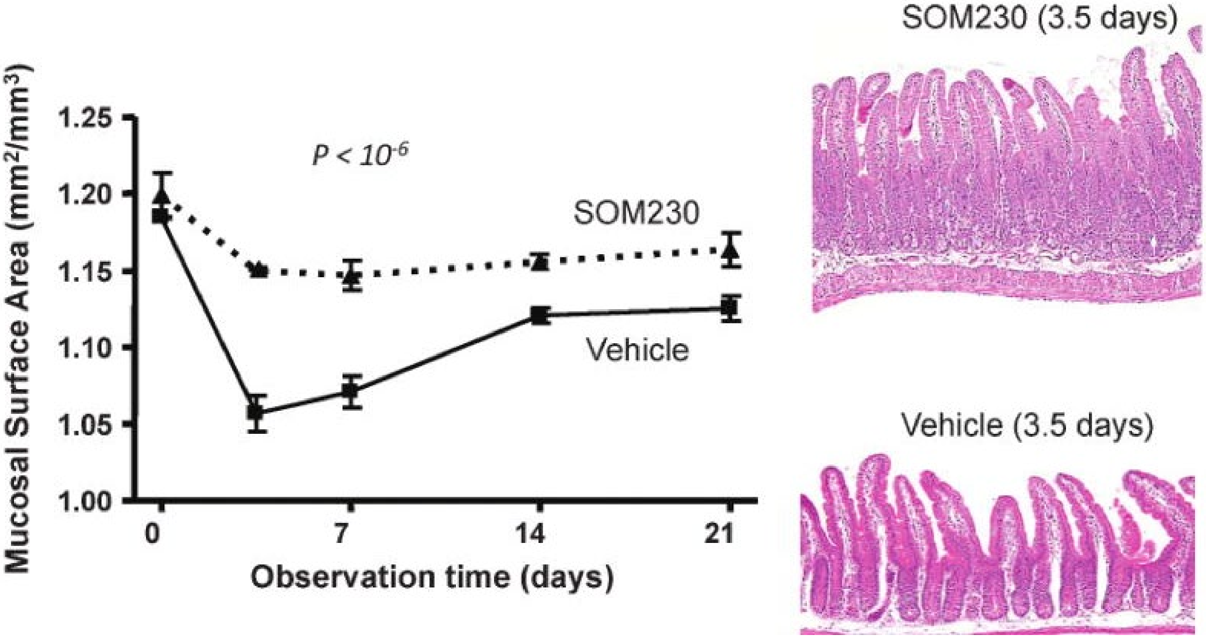

Figure 1. Effect of SOM230 administration on mucosal surface area. Panel A: TBI (8 Gy) induced a reduction in mucosal surface area. SOM230 administration significantly diminished the radiation-induced decrease in mucosal surface area ($P < 10^{-6}$) (n = 6). Panel B: Representative image of intestine from a vehicle-treated animal on day 3.5 after 8 Gy TBI. Panel C: Representative image of intestine from an SOM230-treated animal on day 3.5 after 8 Gy TBI. SOM230: 0.5 mg/kg b.i.d.

The beneficial effects of SOM230 were seen even when administered as late as 24 hrs. after total body irradiation.

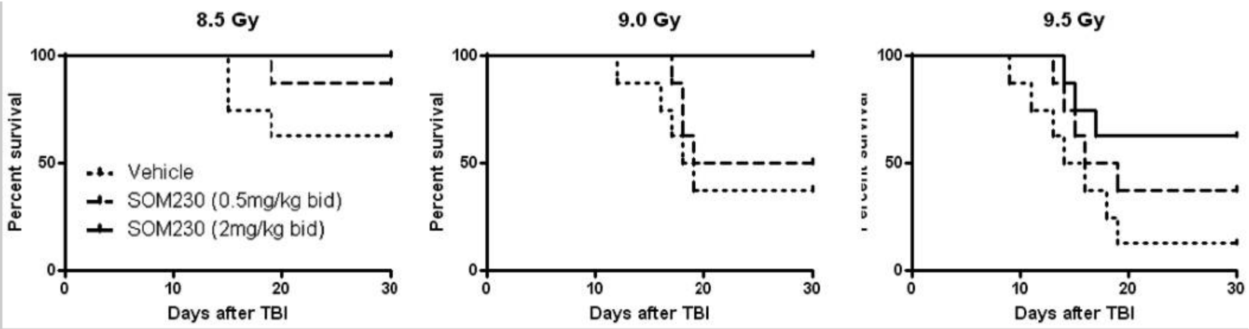

Figure 2. Dose-dependent radiation-mitigating effects of SOM230. Kaplan-Meier survival curves from mice exposed to 8.5, 9 and 9.5 Gy radiation (SOM230 administration beginning 24 h after TBI). SOM230 significantly reduced lethality in a dose-dependent manner across radiation doses ($P < 0.0001$). n = 8.

Pasireotide has recently been approved for the treatment of adult patients with Cushing's disease for whom pituitary surgery is not an option or has not been curative. Pasireotide, with its ability to perform a near complete chemical pancreatectomy, is an excellent candidate for mitigation of the toxicities associated with TBI or high dose chemotherapy. The impact on the GI tract is likely related to pasireotide's ability to block the somatostatin receptor sst 2 leading to the cessation of export of pancreatic enzymes. Moreover, its advanced development as an agent for mitigation of GI toxicities after unplanned radiation exposure further adds excitement for its use in a clinical trial.

5 DESCRIPTION OF PASIREOTIDE

Pasireotide (pasireotide) injection is prepared as a sterile solution of pasireotide diaspartate in a tartaric acid buffer for administration by subcutaneous injection. Pasireotide is a somatostatin analog. Pasireotide diaspartate, chemically known as (2-Aminoethyl) carbamic acid (2R,5S,8S,11S,14R,17S,19aS)-11-(4-aminobutyl)-5-benzyl-8-(4-benzyloxybenzyl)-14- (1H-indol-3-ylmethyl)-4,7,10,13,16,19-hexaoxo-17-phenyloctadecahydro-3a,6,9,12,15,18- hexaazacyclopentacyclooctadecen-2-yl ester, di[(S)-2-aminosuccinic acid] salt, is a cyclohexapeptide with pharmacologic properties mimicking those of the natural hormone somatostatin.

The molecular formula of pasireotide diaspartate is $C_{58}H_{66}N_{10}O_9 \cdot 2\ C_4H_7NO_4$ and the molecular weight is 1313.41.

The structural formula is:

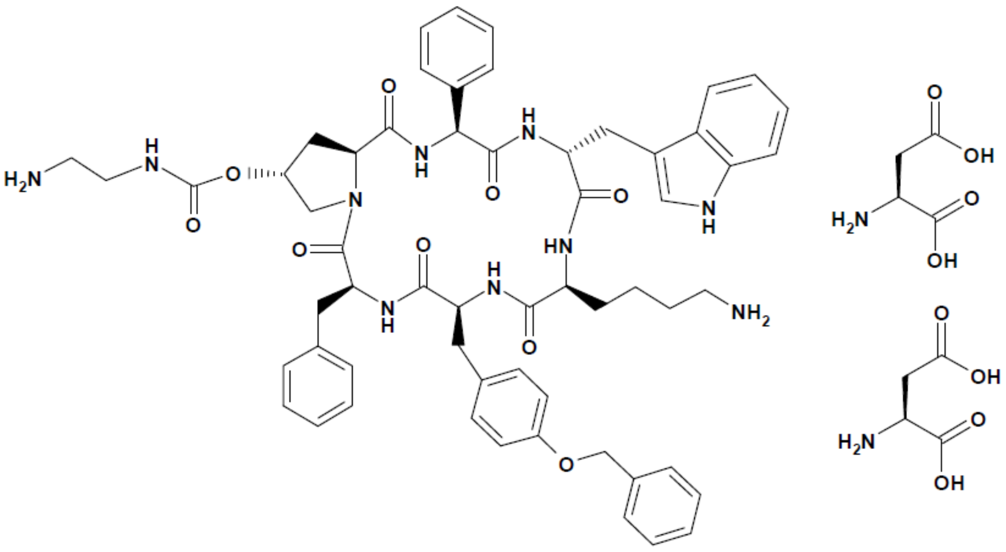

Pasireotide is supplied as a sterile solution in a single-dose, 1 mL colorless glass ampule containing pasireotide in 0.3 mg/mL, 0.6 mg/mL, or 0.9 mg/mL strengths for subcutaneous injection.

Each glass ampule contains:

	0.3 mg	0.6 mg	0.9 mg
Pasireotide	0.3762*	0.7524*	1.1286*
Mannitol	49.50	49.50	49.50
Tartaric acid	1.501	1.501	1.501
Sodium hydroxide	ad pH 4.2	ad pH 4.2	ad pH 4.2
Water for injection	ad 1ml	ad 1ml	ad 1ml

* corresponds to 0.3/0.6/0.9 mg pasireotide base

Note: Each ampule contains an overfill of 0.1ml to allow accurate administration of 1 ml from the ampule.

5.1 Mechanism of Action

Pasireotide is an injectable cyclohexapeptide somatostatin analogue. Pasireotide exerts its pharmacological activity via binding to somatostatin receptors. Five human somatostatin receptor subtypes are known: sst 1, 2, 3, 4, and 5.

These receptor subtypes are expressed in different tissues under normal physiological conditions. Pituitary corticotroph tumor cells from Cushing's disease patients frequently over-express sst5 whereas the other receptor subtypes are often not expressed or are expressed at lower levels. Pasireotide binds and activates the sst receptors resulting in inhibition of ACTH secretion, which leads to decreased cortisol secretion.

The binding affinities of endogenous somatostatin and pasireotide are shown in Table 2.

Table 2- Binding affinities of somatostatin (SRIF-14) and pasireotide to the five human sst receptor subtypes (hsst1-5)

Compound	hsst1	hsst2	hsst3	hsst4	hsst5
Somatostatin (SRIF-14)	0.93±0.12	0.15±0.02	0.56±0.17	1.5±0.4	0.29±0.04
Pasireotide	9.3±0.1	1.0±0.1	1.5±0.3	> 1000	0.16±0.01

Results are the mean±SEM of IC_{50} values expressed as nmol/l

5.2 Pharmacodynamics

Cardiac Electrophysiology

QTcF interval was evaluated in a randomized, blinded, crossover study in healthy subjects investigating pasireotide doses of 0.6 mg b.i.d. and 1.95 mg b.i.d. The maximum mean (95% upper confidence bound) placebo-subtracted QTcF change from baseline was 12.7 (14.7) ms and 16.6 (18.6) ms, respectively. Both pasireotide doses decreased heart rate, with a maximum mean (95% lower confidence bound) placebo-subtracted change from baseline of -10.9 (-11.9) beats per minute (bpm) observed at 1.5 hours for pasireotide 0.6 mg bid, and -15.2 (-16.5) bpm at 0.5 hours for pasireotide 1.95 mg b.i.d. The supra-therapeutic dose (1.95 mg b.i.d.) produced mean steady-state Cmax values 3.3-fold the mean Cmax for the 0.6 mg b.i.d. dose in the study.

5.3 Pharmacokinetics

In healthy volunteers, pasireotide demonstrates approximately linear pharmacokinetics (PK) for a dose range from 0.0025 to 1.5 mg. In Cushing's disease patients, pasireotide demonstrates linear dose-exposure relationship in a dose range from 0.3 to 1.2 mg.

5.4 Absorption and Distribution

In healthy volunteers, pasireotide peak plasma concentration is reached within Tmax 0.25-0.5 hour. Cmax and AUC are dose-proportional following administration of single and multiple doses.

No studies have been conducted to evaluate the absolute bioavailability of pasireotide in humans. Food effect is unlikely to occur since pasireotide is administered via a parenteral route.

In healthy volunteers, pasireotide is widely distributed with large apparent volume of distribution (Vz/F >100 L). Distribution between blood and plasma is concentration independent and shows that pasireotide is primarily located in the plasma (91%). Plasma protein binding is moderate (88%) and independent of concentration.

Pasireotide has low passive permeability and is likely to be a substrate of P-gp (P-glycoprotein), but the impact of P-gp on ADME (absorption, distribution, metabolism, excretion) of pasireotide is expected to be low. Pasireotide is not a substrate of efflux transporter BCRP (breast cancer resistance protein), influx transporter OCT1 (organic cation transporter 1), or influx transporters OATP (organic anion-transporting polypeptide) 1B1, 1B3, or 2B1.

5.5 Metabolism and Excretion

Pasireotide was shown to be metabolically stable in human liver and kidney microsomes systems. In healthy volunteers, pasireotide in its unchanged form is the predominant form found in plasma, urine and feces. Somatropin (growth hormone) may increase CYP450 enzymes and, therefore, suppression of growth hormone secretion by somatostatin analogs including pasireotide may decrease the metabolic clearance of compounds metabolized by CYP450 enzymes.

Pasireotide is eliminated mainly via hepatic clearance (biliary excretion) with a small contribution of the renal route. In a human ADME study 55.9 ± 6.63% of the radioactivity dose was recovered over the first 10 days post dosing, including
48.3 ± 8.16% of the radioactivity in feces and 7.63 ± 2.03% in urine.

The clearance (CL/F) of pasireotide in healthy volunteers and Cushing's disease patients is ~7.6 liters/h and ~3.8 liters/h, respectively.

5.6 Steady-state pharmacokinetics

Following multiple subcutaneous doses, pasireotide demonstrates linear pharmacokinetics in the dose range of 0.05 to 0.6 mg once a day in healthy volunteers, and 0.3 mg to 1.2 mg twice a day in Cushing's disease patients. Based on the accumulation ratios of AUC, the calculated effective half-life (t1/2,eff) in healthy volunteers was approximately 12 hours (on average between 10 and 13 hours for 0.05, 0.2 and 0.6 mg once a day doses).

5.7 Special Populations

Population PK analyses of pasireotide indicates that body weight, age, and gender do not affect pasireotide pharmacokinetics and there is no meaningful difference in pharmacokinetics between Caucasian and non-Caucasian PK parameters.

5.8 Hepatic impairment

In a clinical study in subjects with impaired hepatic function (Child-Pugh A, B and C), subjects with moderate and severe hepatic impairment (Child-Pugh B and C) showed significantly higher exposures than subjects with normal hepatic function. Upon comparison with the control group, AUCinf was increased by 12%, 56% and 42% and Cmax increased by 3%, 46% and 33%, respectively, in the mild, moderate and severe hepatic impairment groups.

5.9 Renal impairment

Clinical pharmacology studies have not been performed in patients with impaired renal function. However, renal clearance has a minor contribution to the elimination of pasireotide in humans. Renal function is not expected to significantly impact the circulating levels of pasireotide [see Use in Specific Populations (8.7)].

5.10 Pediatric Patients

No studies have been performed in pediatric patients.

5.11 Geriatric patients

No clinical pharmacology studies have been performed in geriatric patients.

5.12 Drug Interaction Studies:

There was no significant drug interaction between pasireotide and metformin, nateglinide or liraglutide.

5.13 Rationale for dose in this study:

In a phase III study of Cushing's Disease ([SOM230B2216] and [SOM230B2124] patients randomized to 0.6 mg bid and 0.9 mg bid, adverse events were similar between doses (Table 1, page 18). Therefore, in order to maximize the potential for clinical efficacy, and because we do not think there would be a significant difference in toxicity, we chose to use 0.9 mg bid as the starting dose. If significant toxicities occur, we could consider reducing to 0.6 mg bid, but we believe there is sufficient safety data to justify 0.9 mg bid as the starting dose.

6 NONCLINICAL TOXICOLOGY

6.1 Carcinogenesis

A life-time carcinogenicity study was conducted in rats and transgenic mice. Rats were given daily subcutaneous doses of pasireotide at 0.01, 0.05, 0.3 mg/kg/day for 104 weeks. There were no drug-related tumors in rats at exposures up to 7- fold higher than the maximum recommended clinical exposure at the 1.8 mg/day dose. Mice were given subcutaneous doses of pasireotide at 0.5, 1.0, 2.5 mg/kg/day for 26 weeks and did not identify any carcinogenic potential.

6.2 Mutagenesis

Pasireotide was not genotoxic in a battery of in vitro assays (Ames mutation test in Salmonella and E coli. and mutation test in human peripheral lymphocytes). Pasireotide was not genotoxic in an in vivo rat bone marrow nucleus test.

6.3 Impairment of Fertility

Subcutaneous dosing at 0.1 mg/kg/day before mating and continuing into gestation in rats at exposures less than the human clinical exposure based on body surface area comparisons across species resulted in statistically significant increased implantation

loss and decreased viable fetuses, corpora lutea, and implantation sites. Abnormal cycles or acyclicity were observed at 1 mg/kg/day (5-fold higher than the maximum therapeutic exposure based on surface area, comparisons across species).

7 CLINICAL STUDIES

A Phase III, multicenter, randomized study was conducted to evaluate the safety and efficacy of two dose levels of pasireotide over a 6-month treatment period in Cushing's disease patients with persistent or recurrent disease despite pituitary surgery or *de novo* patients for whom surgery was not indicated or who had refused surgery.

Patients with a baseline 24-hour urine free cortisol (UFC) >1.5 x upper limit of normal (ULN) were randomized to receive a pasireotide dosage of either 0.6 mg subcutaneous b.i.d. or 0.9 mg subcutaneous b.i.d. After three months of treatment, patients with a mean 24-hour UFC ≤ 2.0 x ULN and below or equal to their baseline values continued blinded treatment at the randomized dose until Month 6. Patients who did not meet these criteria were unblinded and the dose was increased by 0.3 mg b.i.d. After the initial six months in the study, patients entered an additional 6-month open-label treatment period. The dosage could be reduced by 0.3 mg b.i.d. at any time during the study for intolerability.

A total of 162 patients were enrolled in this study. The majority of patients were female (78%) and had persistent or recurrent Cushing's disease despite pituitary surgery (83%) with a mean age of 40 years. A few patients (4%) in either treatment group received previous pituitary irradiation. The median value of the baseline 24-hour UFC for all patients was 565 nmol/24 hours (normal range 30 to 145 nmol/24 hours). About two-thirds of all randomized patients completed six months of treatment.

The primary efficacy endpoint was the proportion of patients who achieved normalization of mean 24-hour UFC levels after six months of treatment and did not dose increase during this period.

24-Hour Urinary Free Cortisol Results

At Month 6, the percentages of responders for the primary endpoint were 15% and 26% in the 0.6 mg b.i.d. and 0.9 mg b.i.d. groups, respectively (Table 3). The percentages of patients with mUFC ≤ ULN or ≥ 50% reduction from baseline, a less stringent endpoint than the primary endpoint, were 34% in the 0.6 mg bid and 41% in the 0.9 mg bid groups. Dose increases appeared to have minimal effect on 24-hour UFC response. Mean and median percentage changes in UFC from baseline are presented in Table 3.

Table 3- 24-Hour Urinary Free Cortisol (UFC) Study Results at Month 6 in Patients with Cushing's Disease		
	**Pasireotide 0.6 mg b.i.d. N=82**	**Pasireotide 0.9 mg b.i.d. N=80**
**UFC Responders n/N**<br>**% (95% CI)**	12/82<br>15% (7%, 22%)	21/80<br>26% (17%, 36%)
**UFC Levels** (nmol/24hr) **Baseline**	N=78	N=72
**Mean (SD)**	868 (764)	750 (930)
**Median**	704	470
**% Change from baseline Mean (95% CI) Median**	-22% (-44%, +1%)<br>-47%	-42% (-50%, -33%)<br>-46%

Pasireotide resulted in a decrease in the mean 24-hour UFC after 1 month of treatment (Figure 1). For patients (n=78) who stayed in the trial, similar UFC lowering was observed at Month 12.

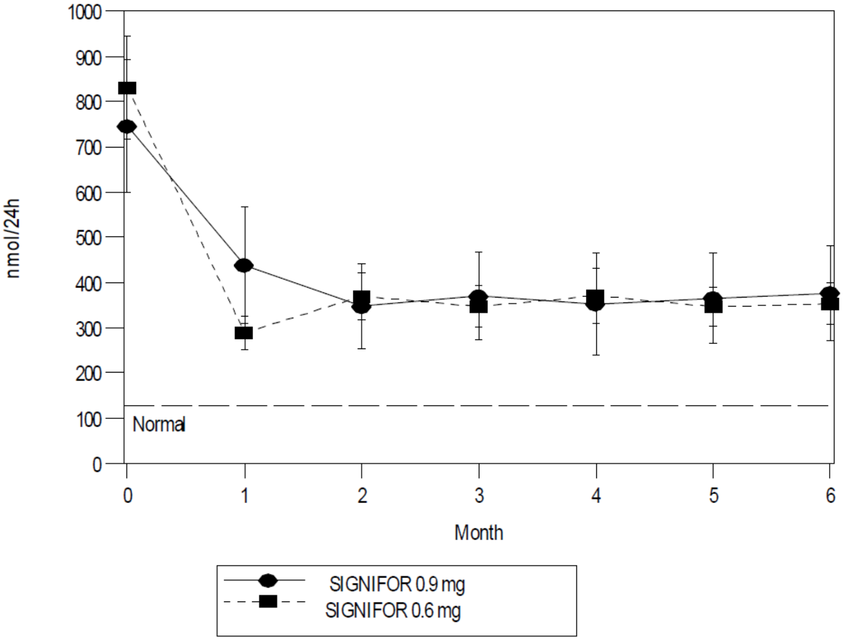

Figure 1 - Mean (±SE) Urinary Free Cortisol (nmol/24h) at time points up to Month 6 by randomized dose group

Note: Only patients who completed 6 months of treatment are included in this analysis (n=110). The reference line is the upper limit of normal for UFC, which is 145 nmol/24hour; +/-Standard errors are displayed.

Other endpoints

Decreases from baseline for blood pressure were observed at Month 6, including patients who did not receive any antihypertensive medication. However, due to the fact

that the study allowed initiation of antihypertensive medication and dose increases in patients already receiving such medications, the individual contribution of pasireotide or of antihypertensive medication adjustments cannot be clearly established.

The mean decreases from baseline at Month 6 for weight, body mass index and waist circumference were 4.4 kg, 1.6 kg/m2 and 2.6 cm, respectively. Individual patients showed varying degrees of improvement in Cushing's disease manifestations but because of the variability in response and the absence of a control group in this trial, it is uncertain whether these changes could be ascribed to the effects of pasireotide.

7.1 Clinical Studies in Children

Safety and effectiveness of pasireotide have not been established in pediatric patients.

7.2 Geriatric Use

Clinical studies of pasireotide did not include sufficient numbers of patients aged 65 and over to determine whether they respond differently from younger patients. Other reported clinical experience has not identified differences in responses between the elderly and younger patients. In general, dose selection for an elderly patient should be cautious, usually starting at the low end of the dosing range, reflecting the greater frequency of decreased hepatic, renal, or cardiac function, and concomitant disease or other drug therapy.

8 STUDY DRUG INFORMATION

8.1 Dosage

The dose of pasireotide to be used in this study is 0.9 mg by subcutaneous injection every 12 hours. It will be supplied in 0.9 mg/mL in a single-dose, 1 mL colorless glass ampules.

It is expected that patients will receive their pasireotide injections in the hospital and that these will be administered by medical staff. However, for patients who are not admitted and receive their pasireotide at home (e.g. the night before admission for conditioning), they will be taught by medical staff how to self-administer pasireotide. As a subcutaneous injection, this process is similar to giving self-injections of insulin and is done routinely by patients with Cushing’s who are receiving pasireotide. Note that this does not apply to the first dose: as per protocol, all first doses of pasireotide will be given by medical personnel in the hospital or clinic with EKG monitoring.

8.2 Renal or hepatic insufficiency:

No dosage adjustment of pasireotide in patients with impaired renal function is required.

Dose adjustment is not required in patients with mild impaired hepatic function (Child-Pugh A). Patients with moderately impaired hepatic function (Child-Pugh B). and severe hepatic impairment (Child-Pugh C) will be excluded from the study.

8.3 Storage and Handling

Store at 25° C (77°F); excursions permitted to 15°-30°C (59°-86°F), protect from light.

8.4 Adverse Events

Because clinical trials are conducted under widely varying conditions, adverse reaction rates observed in clinical trials of a drug cannot be directly compared to rates in clinical trials of another drug and may not reflect the rates observed in practice. Of note, pasireotide has not been studied in children and the below data are from trials in adults.

A total of 162 Cushing's disease patients were exposed to pasireotide in the Phase III study. At study entry, patients were randomized to receive twice a day (b.i.d.) doses of either 0.6 mg or 0.9 mg of pasireotide given subcutaneously. The mean age of patients was approximately 40 years old with a predominance of female patients (78%). The majority of the patients had persistent or recurrent Cushing's disease (83%) and few patients (≤ 5%) in either treatment group had received previous pituitary irradiation. The median exposure to the treatment was 10.4 months (0.03-37.8) with 68% of patients having at least six months exposure.

In the Phase III trial, adverse reactions were reported in 98% of patients. The most common adverse reactions (frequency ≥20% in either group) were diarrhea, nausea, hyperglycemia, cholelithiasis, headache, abdominal pain, fatigue, and diabetes mellitus. There were no deaths during the study. Serious adverse events were reported in 25% of patients. Adverse events leading to study discontinuation were reported in 17% of patients.

Adverse reactions with an overall frequency higher than 5% are presented in Table 1 by randomized dose group and overall. Adverse reactions are ranked by frequency, with the most frequent reactions listed first.

Table 1 - Adverse reactions [n (%)] with an overall frequency of more than 5% in the combined dose group in the Phase III study in Cushing's disease patients

	Pasireotide 0.6 mg bid N=82	Pasireotide 0.9 mg bid N=80	Overall N=162
Diarrhea	48 (59)	46 (58)	94 (58)
Nausea	38 (46)	46 (58)	84 (52)
Hyperglycemia	31 (38)	34 (43)	65 (40)
Cholelithiasis	25 (30)	24 (30)	49 (30)
Headache	23 (28)	23 (29)	46 (28)
Abdominal pain	19 (23)	20 (25)	39 (24)
Fatigue	12 (15)	19(24)	31 (19)
Diabetes mellitus	13 (16)	16 (20)	29 (18)
Injection site reactions	14 (17)	14 (18)	28 (17)
Nasopharyngitis	10 (12)	11 (14)	21 (13)

Alopecia	10 (12)	10 (13)	20 (12)
Asthenia	13 (16)	5 (6)	18 (11)
Glycosylated hemoglobin increased	10 (12)	8 (10)	18 (11)
Alanine aminotransferase	11 (13)	6 (8)	17 (10)
Gamma-glutamyl transferase increased	10 (12)	7 (9)	17 (10)
Edema peripheral	9 (11)	8 (10)	17 (10)
Abdominal pain upper	10 (12)	6 (8)	16 (10)
Decreased appetite	7 (9)	9 (11)	16 (10)
Hypercholesterolemia	7 (9)	9 (11)	16 (10)
Hypertension	8 (10)	8 (10)	16 (10)
Dizziness	8 (10)	7 (9)	15 (9)
Hypoglycemia	12 (15)	3 (4)	15 (9)
Type 2 diabetes mellitus	10 (12)	5 (6)	15 (9)
Anxiety	5 (6)	9 (11)	14 (9)
Influenza	9 (11)	5 (6)	14 (9)
Insomnia	3 (4)	11 (14)	14 (9)
Myalgia	10 (12)	4 (5)	14 (9)
Arthralgia	5 (6)	8 (10)	13 (8)
Pruritus	6 (7)	7 (9)	13 (8)
Lipase increased	7 (9)	5 (6)	12 (7)
Constipation	7 (9)	4 (5)	11 (7)
Hypotension	5 (6)	6 (8)	11 (7)
Vomiting	3 (4)	8 (10)	11 (7)
Back pain	4 (5)	6 (8)	10 (6)
Dry skin	5 (6)	5 (6)	10 (6)
Electrocardiogram QT prolonged	5 (6)	5 (6)	10 (6)
Hypokalemia	6 (7)	4 (5)	10 (6)
Pain in extremity	6 (7)	4 (5)	10 (6)
Sinus bradycardia	8 (10)	2 (3)	10 (6)
Vertigo	4 (5)	6 (8)	10 (6)
Abdominal distension	4 (5)	5 (6)	9 (6)
Adrenal insufficiency	4 (5)	5 (6)	9 (6)
Aspartate aminotransferase increased	6 (7)	3 (4)	9 (6)
Blood glucose increased	6 (7)	3 (4)	9 (6)

Other notable adverse reactions which occurred with a frequency less than 5% were: anemia (4%); blood amylase increased (2%) and prothrombin time prolonged (2%).

Gastrointestinal Disorders

Gastrointestinal disorders, predominantly diarrhea, nausea, abdominal pain and vomiting were reported frequently in the Phase III trial (see Table 1). These events

began to develop primarily during the first month of treatment with pasireotide and required no intervention.

Hyperglycemia and Diabetes
Hyperglycemia-related terms were reported frequently in the Phase III trial. For all patients, these terms included: hyperglycemia (40%), diabetes mellitus (18%), increased HbA1c (11%), and type 2 diabetes mellitus (9%). In general, increases in fasting plasma glucose (FPG) and hemoglobin A1c (HbA1c) were seen soon after initiation of pasireotide and were sustained during the treatment period. In the pasireotide 0.6 mg group, mean fasting plasma glucose (FPG) levels increased from 98.6 mg/dL at baseline to 125.1 mg/dL at Month 6. In the pasireotide 0.9 mg group, mean fasting plasma glucose (FPG) levels increased from 97.0 mg/dL at baseline to 128.0 mg/dL at Month 6. In the pasireotide 0.6 mg group, HbA1c increased from 5.8% at baseline to 7.2% at Month 6. In the pasireotide 0.9 mg group, HbA1c increased from 5.8% at baseline to 7.3% at Month 6.

At one month follow-up visits following discontinuation of pasireotide, mean FPG and HbA1c levels decreased but remained above baseline values. Long-term follow-up data are not available.

Elevated Liver Tests
In the Phase III trial, there were transient mean elevations in aminotransferase values in patients treated with pasireotide. Mean values returned to baseline levels by Month 4 of treatment. The elevations were not associated with clinical symptoms of hepatic disease.

In the clinical development program of pasireotide, there were 4 patients with concurrent elevations in ALT greater than 3 x ULN and bilirubin greater than 2 x ULN: one patient with Cushing's disease and three healthy volunteers. In all four cases, the elevations were noted within the first 10 days of treatment. In all of these cases, total bilirubin elevations were seen either concomitantly or preceding the transaminase elevation. The patient with Cushing's disease developed jaundice. All four cases had resolution of the laboratory abnormalities with discontinuation of pasireotide.

Hypocortisolism
Cases of hypocortisolism were reported in the Phase III study in Cushing's disease patients. The majority of cases were manageable by reducing the dose of pasireotide and/or adding low-dose, short-term glucocorticoid therapy.

Injection Site Reactions
Injection site reactions were reported in 17% of patients enrolled in the Phase III trial in Cushing's disease. The events were most frequently reported as local pain, erythema, hematoma, hemorrhage, and pruritus. These events resolved spontaneously and required no intervention.

Thyroid function

Hypothyroidism with the use of pasireotide was reported for seven patients participating in the Phase III study in Cushing's disease. All seven patients presented with a TSH close to or below the lower limit at study entry which precludes establishing a conclusive relationship between the adverse event and the use of pasireotide.

Other Abnormal Laboratory Findings
Asymptomatic and reversible elevations in lipase and amylase were observed in patients receiving pasireotide in clinical studies. Pancreatitis is a potential adverse reaction associated with the use of somatostatin analogs due to the association between cholelithiasis and acute pancreatitis.

For hemoglobin levels, mean decreases that remained within normal range were observed. Also, post-baseline elevations in PT and PTT were noted in 33% and 47% of patients, respectively. The PT and PTT elevations were minimal.

These laboratory findings are of unclear clinical significance.

8.5 Drug Interactions

8.5.1 Effects of Other Drugs on Pasireotide

Anti-Arrhythmic Medicines and Drugs that Prolong QT
Co-administration of drugs that prolong the QT interval with pasireotide may have additive effects on the prolongation of the QT interval. Caution is required when co-administering pasireotide with anti-arrhythmic medicines and other drugs that may prolong the QT interval [see Warnings and Precautions (5.3)].

8.5.2 Effects of Pasireotide on Other Drugs

Cyclosporine
Concomitant administration of cyclosporine with pasireotide may decrease the relative bioavailability of cyclosporine and, therefore, dose adjustment of cyclosporine to maintain therapeutic levels may be necessary

Bromocriptine
Co-administration of somatostatin analogues with bromocriptine may increase the blood levels of bromocriptine. Dose reduction of bromocriptine may be necessary.
The use of anticoagulant medication should be avoided. Patients currently receiving supplemental pancreatic enzymes may be enrolled, but they should not start such treatment or increase the dose while on study.

8.6 Contraindications

None

8.7 Warnings

8.7.1 Hypocortisolism

Treatment with pasireotide leads to suppression of adrenocorticotropic hormone (ACTH) secretion in Cushing's disease. Suppression of ACTH may lead to a decrease in circulating levels of cortisol and potentially hypocortisolism.

Monitor and instruct patients on the signs and symptoms associated with hypocortisolism (e.g. weakness, fatigue, anorexia, nausea, vomiting, hypotension, hyponatremia or hypoglycemia). If hypocortisolism occurs, consider temporary dose reduction or interruption of treatment with pasireotide, as well as temporary, exogenous glucocorticoid replacement therapy.

In a phase III study of patients with Cushing's Disease, adrenal insufficiency was noted in 6% of patients that received a median of 10.4 months of pasireotide therapy (0.9mg BID). Hypocortisolism is not expected to occur in this trial due to the relatively short duration of treatment. Therefore, cortisol assessment will not be required on this protocol, however, it may be performed at the investigator's discretion.

8.7.2 Hyperglycemia and Diabetes

Elevations in blood glucose levels have been seen in healthy volunteers and patients treated with pasireotide. In the Phase III trial, the development of pre-diabetes and diabetes was observed [see Clinical Studies (14)]. In this trial, nearly all patients—including those with normal glucose status at baseline, pre-diabetes, and diabetes—developed worsening glycemia in the first two weeks of treatment. Cushing's disease patients with poor glycemic control (as defined by HbA1c values >8% while receiving anti-diabetic therapy) may be at a higher risk of developing severe hyperglycemia and associated complications, e.g. ketoacidosis.

Because of this predictable adverse reaction, the glycemic status [fasting plasma glucose (FPG) or hemoglobin A1c (HbA1c)] should be assessed prior to starting treatment with pasireotide. In patients with uncontrolled diabetes, intensive anti-diabetic therapy should be initiated prior to treatment with pasireotide. Monitoring of blood glucose and/or FPG assessments should be done at baseline, and transplant day 14. During pasireotide administrations, patients will typically be hospitalized as part of their preparative regimen and will follow standard of care hyperglycemia testing and management practices for inpatients.

If hyperglycemia develops in a patient treated with pasireotide, the initiation or adjustment of anti-diabetic treatment is recommended. The optimal treatment for the management of pasireotide-induced hyperglycemia is not known. If uncontrolled hyperglycemia persists, despite appropriate medical management, the dose of pasireotide should be reduced or discontinued.

After treatment discontinuation, glycemic monitoring (e.g. FPG or HbA1c should be done according to clinical practice. Patients who were initiated on anti-diabetic therapy

as a result of pasireotide may require closer monitoring after discontinuation of pasireotide, especially if the anti-diabetic therapy has a risk of causing hypoglycemia.

8.7.3 Bradycardia and QT Prolongation

Bradycardia

Bradycardia has been reported with the use of pasireotide [see Adverse Reactions (6)]. Patients with cardiac disease and/or risk factors for bradycardia, such as history of clinically significant bradycardia, high-grade heart block, or concomitant use of drugs associated with bradycardia, should be carefully monitored. Dose adjustments of beta-blockers, calcium channel blockers, or correction of electrolyte disturbances may be necessary.

QT Prolongation

Pasireotide is associated with QT prolongation. In two thorough QT studies with pasireotide, QT prolongation occurred at therapeutic and supra-therapeutic doses. Pasireotide should be used with caution in patients who are at significant risk of developing prolongation of QTc, such as those [see Clinical Pharmacology (12.2)]:

- with congenital long QT prolongation.
- with uncontrolled or significant cardiac disease including recent myocardial infarction, congestive heart failure, unstable angina or clinically significant bradycardia.
- on anti-arrhythmic therapy or other substances that are known to lead to QT prolongation.
- with hypokalemia and/or hypomagnesemia.

A baseline ECG will be done at screening and at hours 1, 1.5, and 2 after the first dose of pasireotide.. Hypokalemia and hypomagnesemia must be corrected prior to the first dose of pasireotide administration and should be monitored and corrected per institutional practice during therapy. Prolonged QTc should be managed as per section 11.4

8.7.4 Liver Test Elevations

In the Phase III trial, 5% of patients had an ALT or AST level greater than 3 times the upper limit of normal (ULN). In the entire clinical development program of pasireotide, there were 4 cases of concurrent elevations in ALT (alanine aminotransferase) greater than 3 x ULN and bilirubin greater than 2 x ULN: one patient with Cushing's disease and three healthy volunteers [see Adverse Reactions (6)]. In these cases, total bilirubin elevations were seen either concomitantly or preceding the transaminase elevation.

Monitoring of liver tests should be done at baseline and weekly starting day 0 through transplant day +100. If ALT is normal at baseline and elevations of ALT of 3-5 times the ULN are observed on treatment, repeat the test within a week or within 48 hours if exceeding 5 times ULN. If ALT is abnormal at baseline and elevations of ALT of 3-5 times the baseline values are observed on treatment, repeat the test within a week or sooner if exceeding 5 times ULN. Tests should be done in a laboratory that can provide same-day results. If the values are confirmed or rising, interrupt pasireotide treatment

and investigate for probable cause of the findings, which may or may not be pasireotide-related. Serial measures of ALT, aspartate aminotransferase, alkaline phosphatase, and total bilirubin, should be done weekly, or more frequently, if any value exceeds 5 times the baseline value in case of abnormal baselines or 5 times the ULN in case of normal baselines. If resolution of abnormalities to normal or near normal occurs, resuming treatment with pasireotide may be done cautiously, with close observation, and only if some other likely cause has been found. See section 11.4 for dosing modification guidelines.

8.7.5 Cholelithiasis

Cholelithiasis has been frequently reported in clinical studies with pasireotide [see Adverse Reactions (6)]. For this reason, a gallbladder ultrasound will be performed at baseline (within 30 days prior to starting pasireotide) and between transplant days +4 through +6 after the last day of pasireotide dosing.

8.7.6 Monitoring for Deficiency of Pituitary Hormones

As the pharmacological activity of pasireotide mimics that of somatostatin, inhibition of pituitary hormones, other than ACTH, may occur. Monitoring of pituitary function (e.g., TSH/free T4, GH/IGF-1) should occur at screening and at transplant day +14. . Patients who have undergone transsphenoidal surgery and pituitary irradiation are particularly at increased risk for deficiency of pituitary hormones.

9 PATIENT ELIGIBILITY

9.1 Inclusion Criteria

- 18 years of age or older at the time of study enrollment.
- Histologically confirmed diagnosis for which an allogeneic transplant is utilized.
- Plan to receive an allogeneic transplant from a 4-6/6 single or dual umbilical cord blood graft, or a 7-8/8 HLA-matched sibling or unrelated donor (High resolution HLA-A, B, C, DRB1).
- Meet standard criteria as defined by the institution for a myeloablative allogeneic stem cell transplantation, with myeloablative defined as using conditioning regimens containing:
  - o TBI ≥ 1200 cGy, or
  - o Busulfan ≥ 12.8mg/kg
- Patient must have given written informed consent according to FDA guidelines.
- Willingness and ability to comply with scheduled visits, treatment plans, laboratory tests and other study procedures.

9.2 Exclusion Criteria

- Female patients who are pregnant or lactating, or are of childbearing potential (FCBP, defined as all women physiologically capable of becoming pregnant) and not practicing an effective method of contraception/birth control
  - • FCBP must have a current negative serum pregnancy test prior to transplant per institutional practice.
- Use of an investigational drug within 1 month prior to dosing.

- Concurrent enrollment on other clinical research studies that contain an interventional therapy is not permitted while subjects are receiving pasireotide or within 5 half-lives of finishing pasireotide. However, subjects may concurrently enroll in non-interventional studies (e.g. biobanking, mobile health tracking).
- Active CNS disease (related to primary malignancy) at the time of enrollment.
- Patients with existing grade 2 toxicities, except as approved by the investigator.
- Any of the following diseases or conditions:
    - Cardiac:
        - History of unexplained syncope or family history of idiopathic sudden death.
        - Sustained or clinically significant cardiac arrhythmias.
        - Risk factors for Torsades de Pointes such as:
            - Uncontrolled hypokalemia
            - Uncontrolled hypomagnesemia or hypermagnesemia
            - Cardiac failure (New York Heart Association Class II or higher)
            - Clinically significant/symptomatic bradycardia (HR<50), or high-grade AV block.
            - Known diagnosis of QT prolongation (QTc ≥470) or family history of long QT syndrome
            - Concomitant disease(s) that could prolong QT such as autonomic neuropathy (caused by diabetes, or Parkinson's disease), HIV, cirrhosis, uncontrolled hypothyroidism or cardiac failure.
            - Concomitant medications known to prolong the QT interval during the same time as pasireotide is to be administered (unless approved by PI and QTc <470; standard transplant medications that are known to prolong the QT (e.g. azoles, ondansetron, etc.) are permitted but caution is advised and patients should be closely monitored).
    - Endocrine:
        - Uncontrolled diabetes at the time of cytoreduction. All patients with diabetes must be optimized on their diabetes regimen prior to initiating pasireotide.
            - If a patient is diabetic: uncontrolled diabetes as defined by HbA1c > 8%* despite adequate therapy
        - Patients who are not biochemically euthyroid. Patients with known history of hypothyroidism are eligible if they are on adequate and stable replacement thyroid hormone therapy for at least 3 months.
        - Known diagnosis of hypocortisolism
        - Known diagnosis of pituitary hormone deficiency.
        - Known hypersensitivity to somatostatin analogs or any component of the pasireotide LAR or s.c. formulations.
    - Infectious:
        - Uncontrolled (not being treated) infections at the time of cytoreduction.

        - A positive HIV test result (ELISA and Western blot) or history of known HIV. An HIV test will not be required; however, previous medical history will be reviewed.
    - Gastrointestinal:
        - Moderately impaired hepatic function (Child-Pugh B) or severe hepatic impairment (Child-Pugh C)
        - Known gallbladder or bile duct disease, symptomatic cholelithiasis, acute or chronic pancreatitis.
        - Known malabsorption syndrome, short bowel or chologenic diarrhea not controlled by specific therapeutic means.
    - Hematologic:
        - Abnormal coagulation (PT or aPTT >30% above normal limits).
        - Continuous anticoagulation therapy. Patients who were on anticoagulant therapy must complete a washout period of at least 10 days and have confirmed normal coagulation parameters before study inclusion.
    - Miscellaneous:
        - Major surgery/surgical therapy for any cause within 1 month prior to pasireotide administration. Patients should have recovered and have a good clinical condition before entering the study.
        - Any co-morbid condition which, in the view of the Principal Investigator, renders the patient at high risk from treatment complications.
- Patients with a history of non-compliance to medical regimens or who are considered potentially unreliable or will not be able to complete the entire study.

9.3 Patient Numbering

Each subject consented to this study will be assigned a unique study ID consisting of the abbreviation of the site name followed by consecutive three-digit numbering.

In the case of screen failures, study IDs will not be reassigned and patient numbering will continue with the next consecutive number at that site. For example:

Duke	Massachusetts General
DUK-001	MGH-001
DUK-002	MGH-002
DUK-003	MGH-003

10 MATERIAL AND DATA

This protocol will require collection of basic demographic data, data on GVHD, and all toxicity data (related and unrelated to pasireotide).

Study-specific data to be collected following initiation of study pasireotide:

- Basic demographic information to be collected at screening.
- Data will be recorded regarding the subject's conditioning regimen, GVHD prophylaxis, and any GVHD treatment received.
- Routine labs and LFTs will be collected at screening, baseline, and transplant Days 0, +7, +14, continuing weekly through Day +100.
- Serum hCG pregnancy test will be performed at screening.
- Fasting plasma glucose or HbA1c, TSH, Free T4, Growth Hormone, and IGF-1 to be assessed at screening and on transplant Day +14.
- Plasma citrulline samples will be collected at baseline and on transplant days 0, +7, and +14.
- Fecal calprotectin samples will be collected at baseline and on transplant days 0, +7, and +14. A +/- 1 day window is allowed for the collection of fecal samples.
- 12-Lead ECGs will be completed at screening, and at 1 hour, 1.5 hours, and 2 hours after the first dose of pasireotide..
- A gallbladder ultrasound will be completed at baseline (acceptable to perform within 30 days prior to pasireotide initiation) and transplant days +4 through +6.
- Toxicity assessments will be completed at baseline, *daily* while on pasireotide and recorded at transplant days +7, +14, +21, +28 and +35. The maximum grade of each toxicity occurring in the preceding period will be recorded at each assessment point.
- Starting at Transplant Day 0, acute graft-versus-host disease is to be graded weekly through day +100, and on Day +180, and +365 using the BMT CTN Manual of Procedures, Version 3 (BMT CTN, 2013)[17]. The maximum grade of GVHD that occurred during each assessment period should be reported.
- Chronic graft-versus-host disease is to be graded at Transplant Day +100, +180, +270, and +365 using the 2014 NIH Consensus Criteria (Jagasia et. Al., 2014)[18]. The maximum grade of GVHD that occurred during each assessment period should be reported.
- Overall and disease-free survival, along with cause of death (if applicable) is to be assessed at transplant day +100, +180, and +365.

Duke Only:

- A subset of 10 patients will complete a video capsule endoscopy between Transplant Days +4 through +6.

11 TREATMENT PLAN AND MODIFICATIONS

The plan would be an initial phase II study to measure efficacy, as well as toxicity, of pasireotide in decreasing grade 3-4 GI toxicities through Transplant Day +30.

Study Phase:

The proposed study will be a phase II, non-randomized study. If the phase II study demonstrates improved efficacy, we would propose to move this to a phase III. Data collected from these patients would be significant to help inform the design and potential efficacy of this drug.

11.1 Study Design:

The study design will be a non-randomized phase II. Forty patients receiving an ablative preparatory regimen will receive pasireotide subcutaneous (0.9 mg Q12H.) one day prior to initiation of the preparatory regimen (at least 15 hours prior to initiation of conditioning) and continuing for four days following stem cell transplant not to exceed 14 days total dosing of pasireotide (i.e. stop on Day +4 after transplant or Day 14 of pasireotide, whichever is sooner - maximum of 28 doses). Myeloablative preparatory regimens are defined as those including either TBI ≥ 1200 cGy or busulfan ≥ 12.8 mg/kg. We will select matched controls from existing patients who did not enroll on the pasireotide study to minimize the time it takes to complete the trial.

The most common regimens combine TBI with cyclophosphamide (TBI/Cy) or busulfan with cyclophosphamide (Bu/Cy) (Appendix E). However, any regimen meeting the above definition of myeloablative preparatory regimen may be used.

The study will collect data at screening, at baseline prior to initiation of the drug (day of study drug start), transplant day 0, day +7, day +14 and weekly thereafter until day +100, and on days +180, +270, and +365.. The total days on pasireotide therapy will be recorded as well as any SAE that is outside the expected for stem cell transplantation. We will also follow the incidence and severity of acute and chronic GVHD.

11.2 Video Capsule Endoscopy

At Duke only, a video capsule endoscopy will be performed in a subset of ten study patients between transplant days +4 through +6. This substudy is descriptive in nature and only used to collect a source of preliminary data that may suggest further study.

Patients must agree to participate in this portion of the study and will be asked to sign a clinical consent for performance use of the video capsule endoscopy. Patients will be given detailed instructions to prepare for the procedure (Appendix B).

An investigator who is blinded to the group allocation of the patients/volunteers separately will review the images obtained from each of the capsule examinations. Images will be examined for evidence of the four following types of abnormalities: reddened/edema/villous blunting, erosion, ulcer and stenosis. Each of these categories will be scored from 0-3 and summed to obtain an overall index that will range from 0 (normal study) to 12 (severely abnormal in all categories).

A baseline video capsule endoscopy will not be performed, as at baseline patients will not yet have begun myeloablative conditioning (e.g., pasireotide starts Day -5, myeloablative conditioning starts Day -4). Therefore, endoscopies should be normal in all patients (all scores = 0). As a result, we do not think it is ethical to subject patients to

a video capsule endoscopy at baseline as we do not think there is any scientific benefit. Furthermore, we do not propose to compare video capsule endoscopy at end of trial to baseline; rather, we propose to compare video capsule endoscopy at transplant Day 4-6 in 10 treated patients to Day 4-6 in 5 untreated control patients.

Of note, because citrulline and calprotectin may vary at baseline, and because we do plan to evaluate changes in these studies over time, we will collect these studies at baseline, Day 0, Day +7, and Day +14.

11.3 Biomarker Assays

11.3.1 Citrulline assay
Measurement of citrulline concentration has been used as a marker for cytotoxic treatment-induced intestinal damage and it is highly reproducible. The citrulline concentration appears to be a quantitative parameter that is independent of the underlying cause for epithelial cell loss and functions well in the post-SCT setting [13].

- Citrulline Assay: Collect one 6.0ml Sodium Heparin (NaH – green top) tube at each timepoint. Sample should be labeled with patient's study ID, sample type, and date and time of sample collection. Process sample within 2 hours.
    - Thoroughly mix the blood by inverting the tube 8 to 10 times.
    - Centrifuge at approximately 2500 rpm for 10 minutes until serum is well separated.
    - Use pipette to aliquot the plasma evenly into 3 sterile 2ml cryovials.
    - Ensure that each cryovial is labeled with patient's study ID, sample type, and date and time of sample collection; no PHI should be included.
    - Freeze at -80°C until shipment.
    - Ship frozen on dry ice.

11.3.2 Calprotectin assay
Calprotectin has been described as another biomarker of GI injury. During radiation-induced inflammation, leucocytes infiltrate the mucosa and increase the level of fecal calprotectin [15, 16]. Calprotectin will be measured with an ELISA kit (CALPRO, Oslo, Norway) in accordance with the manufacturer's instructions.

- Calprotectin Assay: Collect at least a 50 mg (walnut-sized) stool sample into a sterile specimen container (with no additive) at each timepoint (+/- 24 hours). Sample should be labeled with patient's study ID, sample type, and date and time of sample collection. Refrigerate until processed. Process sample within 24 hours as follows:
    - Stool should be mixed using a disposable sterile spatula (if solid) or dropper (if liquid) and transferred into cryovials using that same spatula or dropper.
    - Aliquot stool sample evenly into 9 sterile 2-ml cryovials.
    - Ensure that each cryovial is labeled with patient's study ID, sample type, and date and time of sample collection; no PHI should be included.

- Freeze at -80°C until shipment.
- Ship frozen on dry ice.

Please ship all samples to the attention of:

Chao Lab c/o Megan Baker
905 S. LaSalle St
Genome Sciences Research Building I, Rm 4022
Durham, NC 27710

11.4 Dose Modifications and Treatment Interruptions/Discontinuation

If the patient experiences toxicities requiring dose reduction of pasireotide, the reduced dose will be maintained for all subsequent doses unless further toxicities occur. No dose re-escalation is permitted.

If pasireotide dosing is held, the study calendar is not affected and all study visits will remain due at the originally scheduled time. Pasireotide therapy should end by Transplant Day +4 or 14 days of treatment (28 total doses), whichever is sooner.

Pasireotide Dose Adjustment Schedule

Dose Level 0 (Starting Dose)	0.9mg Q12H
Dose Level -1	0.6mg Q12H
Dose Level -2	0.3mg Q12H
Dose Level -3	Discontinue Pasireotide

If the patient experiences a toxicity ≥ Grade 3 (CTCAE v.4) suspected to be ***related to pasireotide***, hold pasireotide until the toxicity resolves to ≤ Grade 3 and restart pasireotide with a one level dose reduction according to the table above.

If the patient experiences a toxicity ≥ Grade 4 that is judged to be ***unrelated to pasireotide***, hold pasireotide until the toxicity resolves to < Grade 4 then restart pasireotide at the same dose. No dose reduction is required.

Toxicities of Special Interest

Hyperglycemia – should be managed per institutional practice [guidance for glucose management suggested in Appendix C]

- For Glucose > 250 but < 500 mg/dL, treat according to institutional practice. Pasireotide dosing may continue at same dose.
- For Glucose >500 mg/dL, hold pasireotide and treat according to institutional practice. Pasireotide may be restarted with a one level dose reduction when glucose has been reduced to < 500mg/dL.

Elevated LFTs

- If ALT, AST, or Alkaline Phosphatase is ≥ Grade 3, ***regardless of attribution***, hold pasireotide. Pasireotide may be restarted with a one level dose reduction when toxicity has resolved to < Grade 3.

In addition, if any of the following criteria are met, study drug may be discontinued at the discretion of the investigator:

- Unacceptable toxicity that, in the judgment of the investigator, compromises the ability to continue study-specific procedures or is considered to not be in the subject's best interest
- Subject request to discontinue for any reason
- Proven non-compliance with treatment or scheduled evaluation
- Discontinuation of the study at the request a regulatory agency, or the governing institutional review board (IRB)

11.5 Study Withdrawal

The subject is to be withdrawn from study in the event of:

- Disease relapse,
- Subject never receives pasireotide,
- Graft failure (withdrawal to occur at the time when new antineoplastic therapy or conditioning chemotherapy for a 2nd transplant is initiated),
- Transfer to Hospice,
- or other such events approved by study PI.

12 ANTICIPATED TOXICITIES.

Safety will be assessed based on CTCAE v. 4.0 criteria. Patients receiving stem cell transplantation have an expected rate of events. While we realize 21CFR312.32 (http://www.accessdata.fda.gov/scripts/cdrh/cfdocs/cfcfr/cfrsearch.cfm?fr=312.32) does not draw a distinction between suspected events related to the study drug and suspected events related to normal medical care (i.e. HSCT), because HSCT is associated with such a high rate of expected adverse events, we believe the distinction between "suspected-related to HSCT" and "suspected-related to study drug" is relevant, and prefer this terminology to "expected", "labeled", and "non-labeled" (though we retain the category of "unexpected" as suggested and per 21CFR312.32). We will specifically assess for unexpected adverse events as these patients are closely monitored on a daily basis.

Clinically significant adverse reactions associated with the administration of pasireotide that appear in "Warnings" sections of this protocol include:

- Hyperglycemia and Diabetes
- Bradycardia and QT prolongation
- Liver test elevations
- Cholelithiasis
- Pituitary hormone deficiency

- Hypocortisolism

The following toxicities are anticipated following allogeneic stem cell transplantation and will therefore not be reportable to the IRB unless they occur at a frequency that suggests association with the study drug.

12.1 Myelosuppression:
Following donor stem cell engraftment, persistent pancytopenia is common following allogeneic stem cell transplantation. Marrow aplasia may be incited by an adverse drug reaction or by infection such as cytomegalovirus.

12.2 Gastrointestinal:
Nausea and vomiting can be expected during the post-transplant period. The most common cause for GI disturbance is drug-related or residual mucosal damage from the bone marrow preparative regimen. Mucositis and diarrhea may also occur and will be supported with standard measures.

12.3 Hyperglycemia

Hyperglycemia has been observed in patients using pasireotide therapy. Based on this finding, we suggest that optimal glucose control should be achieved before starting a patient on pasireotide.

12.4 Cardiac:
High-dose chemotherapy can cause acute cardiotoxicity in doses used in this protocol (congestive heart failure, pericardial effusion, arrhythmias, EKG changes). Rarely, these may prove to be lethal. Pericardial effusions can also occur as an unusual manifestation of graft versus host disease.

A small number of cases of QT prolongation have been reported in some participants in pasireotide clinical trials. QT is one of the measurements taken during an ECG (heart tracing) and increase (prolongation) in QT may lead to an irregular heartbeat which in rare instances can develop into a sudden, life-threatening condition. The majority of cases that were reported in the pasireotide clinical trials resolved or improved spontaneously without discontinuation of the study drug. Though the risk is small, these findings may indicate a potential risk for an irregular heart rate associated with the treatment with pasireotide.

12.5 Pulmonary:
Pulmonary toxicity from the preparative regimen has been described, and often presents as diffuse alveolar hemorrhage. Hemorrhage may also occur within the first 100 days following transplantation, although the inciting event is likely not the bone marrow preparative regimen. Diffuse interstitial pneumonitis of unknown etiology (infectious, toxic, or multifactorial) is a well-described toxicity of intensive chemotherapy and TBI regimens and may prove lethal. TBI may also cause late pulmonary dysfunction.

12.6 Hepatic:
Tacrolimus or other hepatoxic drugs may result in elevated levels of hepatic enzymes and may also cause elevations in bilirubin.

12.7 Renal:
Mild elevations of BUN and creatinine may be seen in patients treated with high dose chemotherapy and/or Tacrolimus. These are generally reversible. Renal dysfunction, which is usually mild and reversible, may rarely be seen after TBI at the doses used in this protocol. A syndrome of moderate renal insufficiency and hemolysis (Thrombotic-thrombocytopenic purpura/hemolytic uremic syndrome) has been seen 5-18 months post-bone marrow transplantation after intensive multi-agent conditioning +/- TBI. This also is usually reversible, but the long-term sequelae are not yet clear.

12.8 GVHD:
Grade II-IV acute GVHD occurs in 20-40% of patients receiving matched-sibling or partially matched allogeneic stem cell transplantation. GVHD typically causes skin rash, liver function test abnormalities and colitis. The severity of the acute GVHD is quite variable, however the severe cases can be fatal. Chronic GVHD occurs greater than 100 days following stem cell transplantation and affects approximately 50% of recipients. Chronic GVHD is manifested in the skin, liver, mucous membranes and less commonly in the lung and intestinal tract. 10 to 20% of those patients who develop chronic GVHD will ultimately die of complications stemming from this disease.

12.9 Neurologic:
Posterior reversible encephalopathy syndrome (PRES) may occur as a consequence of the total body irradiation.

12.10 Miscellaneous:
- Transfusion reactions
- Glucose intolerance due to multiple medications (e.g. calcineurin inhibitors and glucocorticoids
- Late effects of transplant regimens including: cataracts, infertility, growth impairment, hypothyroidism, and dental caries.
- Headache, insomnia, psychosis, mood changes, disorientation, seizures from metabolic imbalance.
- Well-characterized drug reactions - allergic manifestations, "red man" syndrome. well-characterized drug side effects from drugs used routinely in transplant recipients (e.g.; preparative regimen chemotherapy, immunosuppressive drugs, antimicrobials)
- Common side effects of antiemetics, analgesics, anti-inflammatory agent and known complications of steroid therapy.
- Complications from intravenous catheters, thrombotic occlusion, infection, local reactions, cardiac arrhythmia.Post-transplant microangiopathy.

13 DATA MANAGEMENT AND DATA SAFETY MONITORING

13.1 Data Management

13.1.1 Electronic Case Report Forms (eCRFs)

This study will utilize eCRFs in a REDCap database for data reporting. REDCap is a software tool that does not require client local software and can be accessed from anywhere on the Internet and is secured on a Duke Health Technology Services (DHTS) server. This database will be developed and maintenance performed with support of the School of Medicine (SOM) Duke Office of Clinical Research (DOCR). SOM's DOCR has partnered with the School of Medicine (SOM) to implement REDCap (developed by Vanderbilt's CTSA and currently used and supported by more than 1600 consortium partners. REDCap provides: 1) a stream-lined process for rapidly building a database; 2) an intuitive interface for collecting data (with data validation and audit trail); 3) automated export procedures for seamless data downloads to common statistical packages (SAS, SPSS, etc.); 4) branching logic, file uploading, and calculated fields; and 5) a quick and easy protocol set-up.

REDCap accounts are stored within the DTMI LDAP server hosted by the Duke Office of Information Technology (OIT). Authentication occurs via the OIT implementation of Kerberos. All connections to the system, both external and internal, occur over encrypted channels. Access to components of the system is role-based and can only be granted by administrators of the system. All collected information is stored on a database server hosted by Duke Health Technology Services (DHTS). The database server resides behind the DHTS internal firewall and access to the server is controlled via firewall rules. All collected data are backed up daily, both on the local server and by the DHTS enterprise backup system. The Office of Research Informatics-App Engineering-DHTS (ORI) via ServiceNow (919 668-7286) is responsible for managing the server for REDCap. Duke Office of Clinical Research (Ceci Chamorro 919-668-9262) is responsible for managing the database platform for REDCap. At the time of this submission, REDCap is on version 6.5.15.[19]

The eCRFs will be the primary data collection documents for this study. Sites will update the eCRFs in a timely manner following acquisition of new source data.

13.1.2 Data Management Procedures and Data Verification

Data collected for the study and entered into the RedCap database will be verified against original source documents. Source documentation for study data points may be redacted, labeled with the subjects' study ID, and sent to the coordinating site via email or fax. This source documentation will be filed in individual subject study binders for verification at monitoring visits and study audits.

Alternatively, source documentation may be verified remotely through direct access to the electronic medical record via secure methods approved by Duke and the PI of the study.

13.1.3 Study Closure
Following completion of the studies, the PI will be responsible for ensuring the following activities:

- Data clarification and/or resolution
- Accounting, reconciliation, and destruction/return of used and unused study drugs
- Review of site study records for completeness
- Shipment of all remaining laboratory samples to the designated laboratories

13.2 Data Safety Monitoring
The DCI Monitoring Team will conduct monitoring visits to ensure subject safety and to ensure that the protocol is conducted, recorded, and reported in accordance with the protocol, standard operating procedures, good clinical practice, and applicable regulatory requirements. As specified in the DCI Data and Safety Monitoring Plan, the DCI Monitoring Team will conduct routine monitoring after the third subject is enrolled, followed by annual monitoring of 1- 3 subjects until the study is closed to enrollment and subjects are no longer receiving study interventions that are more than minimal risk.

The Safety Oversight Committee (SOC) will perform annual reviews on findings from the DCI Monitoring Team visit and additional safety and toxicity data submitted by the Principal Investigator.

Additional monitoring may be prompted by findings from monitoring visits, unexpected frequency of serious and/or unexpected toxicities, or other concerns and may be initiated upon request of DUHS and DCI leadership, the CPC, the Safety Oversight Committee (SOC), the sponsor, the Principal Investigator, the Duke Clinical Trials Quality Assurance (CTQA) office or the IRB. All study documents must be made available upon request to the DCI Monitoring Team and other authorized regulatory authorities, which may include but is not limited to the FDA. Every reasonable effort will be made to maintain confidentiality during study monitoring.

14 SAFETY AND STUDY DATA MONITORING

14.1 Safety

14.1.1 Adverse Event Reporting.
The investigator is required by Federal Regulations to report adverse events (AEs) that occur through the duration of the study according to the appropriate procedures listed below. The investigator is required to notify the FDA, and Duke University Health System (DUHS) Institutional Review Board (IRB) Office if an event is serious, unexpected, and related or possibly related to the drug.

We will record all ***grades 2-4*** adverse events ***(excluding lab values)*** from the time of consent through 30 days ***after*** the last dose of pasireotide. It will be noted if the event arose from a preexisting or grade 1 toxicity.

- Each event needs to be categorized as one of the following:
  - Expected – Related to transplant (sections 12.1-12.10)
  - Expected – Related to Pasireotide (section 12.0)
  - Unexpected – Related to Transplant
  - Unexpected – Related to Pasireotide

All "life-threatening" and "serious adverse events" will be reported regardless of suspicion of etiology. In addition, we plan to report "suspected-related to study drug" and "unexpected" events. We do not propose to report adverse events that are "suspected-related to HSCT" that are not "life-threatening" or "serious adverse events" as nearly 100% of patients undergoing HSCT will experience adverse events that are "suspected-related to HSCT" (e.g. historical rate of GI toxicity of myeloablative HSCT itself is 90%, not to mention myelosuppression (100% of patients) and other common adverse events related to HSCT).

Definitions: An adverse event is any untoward medical occurrence associated with the use of a drug in humans, whether or not considered drug related.

A serious adverse event (SAE): an adverse event or suspected adverse reaction is considered "serious" if, in the view of either the investigator, it results in any of the following outcomes:

- Death
- A life-threatening adverse event in the view of the investigator its occurrence places the patient at immediate risk of death
- Inpatient hospitalization or prolongation of existing hospitalization
- A persistent or significant incapacity or substantial disruption of the ability to conduct normal life functions
- A congenital anomaly/birth defect

Important medical events that may not result in death, be life-threatening, or require hospitalization may be considered serious when, based upon appropriate medical judgment, they may jeopardize the patient and may require medical or surgical intervention to prevent one of the outcomes listed above.

Suspected adverse reaction means any adverse event for which there is a reasonable possibility that the drug caused the adverse event. For the purposes of IND safety reporting, "reasonable possibility" means there is evidence to suggest a causal relationship between the drug and the adverse event. Suspected adverse reaction implies a lesser degree of certainty about causality than adverse reaction, which means any adverse event caused by a drug.

Unexpected adverse event or unexpected suspected adverse reaction. An adverse event or suspected adverse reaction is considered "unexpected" if it is not listed in the investigator brochure or is not listed at the specificity or severity that has been observed; or, if an investigator brochure is not required or available, is not consistent with the risk information described in the general investigational plan or elsewhere in the current application.

Any SAE will be reported to Duke, Novartis, and the FDA as soon as possible, but in no case later than 7 calendar days after receipt of the information. Each notification to FDA will be transmitted to the review division in the Center for Drug Evaluation and Research or in the Center for Biologics Evaluation and Research that has responsibility for review of the IND

This study will utilize the NCI Common Toxicity Criteria for Adverse Events (CTCAE) version 4.0 to determine the severity of the reaction for adverse event reporting. Any reaction that is reportable (serious, unexpected and related/possibly related to the study) must be reported using the FDA #3500 MedWatch form and the Novartis SAE coversheets (Appendix H). All AEs reported will be recorded on an electronic AE Case Report Form (CRF).

14.1.2 SAE Reporting

The principal investigator has the obligation to report all serious adverse events to the FDA, IRB, and Novartis Pharmaceuticals Drug Safety and Epidemiology Department (DS&E).

All events reported to the FDA by the investigator are to be filed utilizing the Form FDA 3500A (MedWatch Form) and the Novartis SAE coversheets (Appendix H).

To ensure patient safety, every SAE, regardless of suspected causality, occurring after the patient has provided informed consent and until at least 30 days after the patient has stopped pasireotide must be reported to Duke (**Fax: 919-668-1091**) and Novartis within 24 hours of learning of its occurrence **(Fax: 1-877-778-9739)** using the coversheets found in Appendix H. This includes serious, related, labeled (expected) and serious, related, unlabeled (unexpected) adverse experiences. All deaths during treatment or within 30 days following completion of active protocol therapy must be reported within 5 working days.

Any SAEs experienced after this 30 day period should only be reported to Novartis if the investigator suspects a causal relationship to the study drug. Recurrent episodes, complications, or progression of the initial SAE must be reported as follow-up to the original episode within 24 hours of the investigator receiving the follow-up information. A SAE occurring at a different time interval or otherwise considered completely unrelated to a previously reported one should be reported separately as a new event. The end date of the first event must be provided.

The original copy of the SAE Report and the fax confirmation sheet must be kept within the Trial Master File at the study site.

Follow-up information is sent to the same fax number as the original SAE Report Form was sent, using a new fax cover sheet, stating that this is a follow-up to the previously reported SAE, and giving the date of the original report. Each re-occurrence, complication, or progression of the original event should be reported as a follow-up to that event regardless of when it occurs. The follow-up information should describe whether the event has resolved or continues, if and how it was treated, whether the blind was broken or not (if applicable), and whether the patient continued or withdrew from study participation.

If the SAE is not previously documented in the SOM230 Investigator Brochure or Package Insert (new occurrence) and is thought to be related to the Novartis study drug, a DS&E associate may urgently require further information from the investigator for Health Authority reporting. Novartis may need to issue an Investigator Notification (IN), to inform all investigators involved in any study with the same drug that this SAE has been reported. Suspected Unexpected Serious Adverse Reactions (SUSARs) will be collected and reported to the competent authorities and relevant ethics committees in accordance with Directive 2001/20/EC or as per national regulatory requirements in participating countries.

For Comparator Drugs/Secondary Suspects (Concomitant Medications), all serious adverse experiences will be forwarded to the comparator drug company by the investigator.

14.1.3 Pregnancy

The potential reproductive risk for humans is unknown. Women of childbearing potential should be advised to use highly effective contraception methods while they are receiving SOM230 and up to 8 weeks after treatment has been stopped.

To ensure patient safety, each pregnancy occurring while the patient is on study treatment must be reported to Duke and Novartis within 24 hours of learning of its occurrence. The pregnancy should be followed up to determine outcome, including spontaneous or voluntary termination, details of the birth, and the presence or absence of any birth defects, congenital abnormalities, or maternal and/or newborn complications. The newborn will be followed for at least 12 months.

Pregnancy should be recorded on a Clinical Trial Pregnancy Form and reported by the investigator to Duke and to the oncology Novartis Drug Safety and Epidemiology Department (DS&E). Pregnancy follow-up should be recorded on the same form and should include an assessment of the possible relationship to the study treatment and any pregnancy outcome. Any SAE experienced during pregnancy must be reported on the SAE Report Form.

14.2 Data Monitoring:

This clinical research study will be monitored both internally by the PI and externally by the Duke University Medical Center Cancer Center's Protocol Review and Monitoring system in accord with their NCI- approved "Institutional Protocol Monitoring Procedures and Guidelines for NIH-Sponsored Research Involving Human Subjects."

In terms of internal review, the PI and the Co-PI will continuously monitor and tabulate adverse events. Appropriate reporting to the Duke University Medical Center IRB will be made. If an unexpected frequency of grade III or IV events occur, depending on their nature, action appropriate to the nature and frequency of these adverse events will be taken. This may require a protocol amendment, or potential closure of the study. The severity of adverse events will be classified according to the NCI CTC.

The DCI Monitoring Team will conduct monitoring visits to ensure subject safety and to ensure that the protocol is conducted, recorded, and reported in accordance with the protocol, standard operating procedures, good clinical practice, and applicable regulatory requirements. As specified in the DCI Data and Safety Monitoring Plan, the DCI Monitoring Team will conduct routine monitoring after the third subject is enrolled, followed by annual monitoring of 1 – 3 subjects until the study is closed to enrollment and subjects are no longer receiving study interventions that are more than minimal risk.

The DCI Safety Oversight Committee (SOC) will perform annual reviews on findings from the DCI Monitoring Team visit and additional safety and toxicity data submitted by the Principal Investigator.

Additional monitoring may be prompted by findings from monitoring visits, unexpected frequency of serious and/or unexpected toxicities, or other concerns and may be initiated upon request of DUHS and DCI leadership, the CPC, the Safety Oversight Committee (SOC), the Duke University Office of Audit, Risk, and Compliance- Human Subjects Research Compliance Office, the sponsor, the Principal Investigator, or the IRB. All study documents must be made available upon request to the DCI Monitoring Team and other authorized regulatory authorities, which may include but is not limited to the National Institute of Health, National Cancer Institute, and the FDA. Every reasonable effort will be made to maintain confidentiality during study monitoring.

The Duke University Office of Audit, Risk, and Compliance – Human Subjects Research Compliance (OARC HSRC) office may conduct confidential audits to evaluate compliance with the protocol and the principles of GCP. The PI agrees to allow the OARC HSRC auditor(s) direct access to all relevant documents and to allocate his/her time and the time of the study team to the OARC HSRC auditor(s) in order to discuss findings and any relevant issues.

15 STATISTICAL CONSIDERATIONS

15.1 Objectives

15.1.1 Primary Objective:
To evaluate the efficacy and safety of pasireotide in mitigating GI toxicity from the preparatory regimens of allogeneic stem cell transplantation compared to historical controls.

The control group will be selected from about 400 existing patients who did not enroll on this study. This group will be matched based on age, disease, GVHD prophylaxis, preparatory regimen (TBI or busulfan), graft source, and number of prior therapies.

Efficacy will be assessed as the incidence of all Grade III-IV gastrointestinal toxicities occurring from the baseline visit through transplant day +30. Toxicities will be graded according to CTCAE v. 4.0 and labeled using CTCAE criteria found under the heading of “Gastrointestinal disorders”. This will include symptoms of nausea, vomiting, diarrhea, abdominal pain, distention, mucositis, and the like. It will be noted if the toxicity arose from a preexisting or grade 1 toxicity.

15.1.2 Secondary Objectives:
To evaluate the efficacy of pasireotide in reducing the incidence and severity of acute GVHD at 100 days post-transplant compared to historical controls.

To evaluate the efficacy of pasireotide in reducing the incidence and severity of chronic GVHD at 1 year post-transplant.

To compare the rate of overall survival in patients treated with pasireotide at 1 year post-transplant to historical controls.

To compare the rate of disease free survival in patients treated with pasireotide at 1 year post-transplant to historical controls.

15.2 Exploratory Objectives:
To evaluate plasma citrulline and fecal calprotectin levels as biomarkers of GI tract health and function in SCT patients and the correlation between these biomarkers and GI toxicity. The control population would consist of bone marrow transplant patients, being seen concurrently and who also consented to participate in Pro00006268.

To evaluate GI toxicity assessment by video capsule endoscopy. We will enroll 10 subjects who take pasireotide and 5 controls who do not receive pasireotide.

15.3 Endpoints:

15.3.1 Primary Endpoint
The primary endpoint for the study will be the incidence of grades III-IV GI toxicity in SCT patients who are treated with pasireotide.

We plan to enroll 40 patients in the trial and select 40 matched controls. After collecting data, we will use the Chi-square test to test for the hypothesis H0: p0≤p1 versus H1: p0>p01, where p0 and p1 are the rates of GI toxicity for SCT patients who don't take pasireotide and who take pasireotide, respectively. Historical data show that the rate of GI toxicity without the drug is about 90%, and we expect that the GI toxicity rate in patients who receive pasireotide will decrease to about 50%. With this number of patients, the power of the test is close about 0.99 with a type I error rate 0.05 under our expected rates of GI toxicity. We can still achieve 80% power under a very conservative estimate of GI toxicity rate for those who take drug of 0.65.

15.3.2 Secondary Endpoints

The incidence and severity of acute or chronic GVHD in SCT patients who receive pasireotide. We will test for the hypothesis H0: q0=q1 versus H1: q0>q1 using the Chi-square test, where q1 and q0 are the rates of GVHD in patients with and without pasireotide respectively.

We will compare GVHD incidence rates between treatment group versus historical control group using survival type regression modeling that takes competing risks into account. We will examine cause-specific Cox proportional hazards models as well as the proportional subdistribution hazard regression model developed by Fine and Gray. While the primary treatment comparison will be unadjusted, adjustment for covariates to explore the impact of various risk factors will also be performed.

Survival outcomes will be analyzed in a similar fashion to GVHD, as described above.

15.3.3 Exploratory Endpoints

As exploratory endpoints, citrulline and fecal calprotectin levels will be measured at baseline and transplant day 0, +7, and +14 and descriptive statistics such as means and standard deviations will be calculated. Logistic regression analysis will be performed to evaluate the correlation between the highest measured biomarker level and the incidence of GI toxicity.

Video Capsule

The means of mucositis in the video endoscopy group and the control group will be compared using the Student's t test.

15.4 Stopping Rules

We will monitor 6-month treatment related mortality (TRM) in two stages. At DUMC, the TRM rate has been 0.2 with standard care (consistent with rates in the literature of 16-30%). We will consider a rate of .35 or higher to be unacceptable. We will use the following sequential rules to determine early stopping due to excessive TRM.

**Stage**	**Number of patients accrued**	**Stop trial if 6-month TRM ≥**
1	15	6
2	30	11

This rule will have probability of 0.13 of stopping if the actual TRM rate is at the acceptable rate of 0.20 and a probability of 0.86 of stopping early if the true TRM rate is at the unacceptable rate of 0.35.

16 REFERENCES

1. Dainiak, N., et al., *The hematologist and radiation casualties.* Hematology Am Soc Hematol Educ Program, 2003: p. 473-96.
2. Hauer-Jensen, M., et al., *Radiation damage to the gastrointestinal tract: mechanisms, diagnosis, and management.* Curr Opin Support Palliat Care, 2007. **1**(1): p. 23-9.
3. Nejdfors, P., et al., *Intestinal permeability in humans is increased after radiation therapy.* Dis Colon Rectum, 2000. **43**(11): p. 1582-1587; discussion 1587-8.
4. Morgenstern, L. and N. Hiatt, *Injurious effect of pancreatic secretions on postradiation enteropathy.* Gastroenterology, 1967. **53**(6): p. 923-9.
5. Hauer Jensen, M., et al., *Influence of pancreatic secretion on late radiation enteropathy in the rat.* Acta Radiol Oncol, 1985. **24**(6): p. 555-60.
6. Morgenstern, L., et al., *Prolongation of survival in lethally irradiated dogs by pancreatic duct ligation.* Arch Surg, 1970. **101**(5): p. 586-9.
7. Shinozaki, H., et al., *Effect of somatostatin on pancreatic enzyme secretion.* Gastroenterol Jpn, 1988. **23**(6): p. 673-9.
8. Williams, S.T., et al., *Effect of octreotide acetate on pancreatic exocrine function.* Am J Surg, 1989. **157**(5): p. 459-62.
9. Wang, J., et al., *The synthetic somatostatin analogue, octreotide, ameliorates acute and delayed intestinal radiation injury.* Int J Radiat Oncol Biol Phys, 1999. **45**(5): p. 1289-96.
10. Yavuz, M.N., et al., *The efficacy of octreotide in the therapy of acute radiation-induced diarrhea: a randomized controlled study.* Int J Radiat Oncol Biol Phys, 2002. **54**(1): p. 195-202.
11. Ma, P., et al., *Pharmacokinetic-pharmacodynamic comparison of a novel multiligand somatostatin analog, SOM230, with octreotide in patients with acromegaly.* Clin Pharmacol Ther, 2005. **78**(1): p. 69-80.
12. Fu, Q., et al., *The somatostatin analog SOM230 (pasireotide) ameliorates injury of the intestinal mucosa and increases survival after total-body irradiation by inhibiting exocrine pancreatic secretion.* Radiat Res, 2009. **171**(6): p. 698-707.
13. Lutgens, L.C., et al., *Monitoring myeloablative therapy-induced small bowel toxicity by serum citrulline concentration: a comparison with sugar permeability tests.* Cancer, 2005. **103**(1): p. 191-9.
14. Gupta, P.K., et al., *Development of high-throughput HILIC-MS/MS methodology for plasma citrulline determination in multiple species.* Analytical Methods, 2011. **3**(8): p. 1759-1768.
15. Hille, A., et al., *A prospective study of faecal calprotectin and lactoferrin in the monitoring of acute radiation proctitis in prostate cancer treatment.* Scand J Gastroenterol, 2008. **43**(1): p. 52-8.
16. Larsen, A., et al., *Faecal calprotectin and lactoferrin as markers of acute radiation proctitis: a pilot study of eight stool markers.* Scand J Gastroenterol, 2004. **39**(11): p. 1113-8.
17. Blood and Marrow Transplant Clinical Trials Network, *Technical Manual of Procedures*. 2013. **3**: p. 6-7.

18. Jagasia, M., et al., *National Institutes of Health Consensus Development Project on Criteria for Clinical Trials in Chronic Graft-versus-Host Disease: I. The 2014 Diagnosis and Staging Working Group report.* Biology of Blood and Marrow Transplant. 2015 Mar; **21**(3): p389-401.e1. doi:10.1016/j.bbmt.2014.12.001. Epub 2014 Dec 18.
19. P.A. Harris, R. Thielke, R. Taylor, J. Payne, N. Gonzalez, J.G. Conde. Research Electronic Data Capture (REDCap) - A metadata-driven methodology and workflow process for providing translational research informatics support. Journal of Biomedical Informatics, 2008 (doi:10.1016/j.jbi.2008.08.010).

APPENDIX A: Study Calendar for Special Studies

Assessments solely related to the stem cell transplant will be done per institutional standards and are not included below.

**Studies specific for pasireotide**	**Screening[10]**	**Baseline/Start of Study Drug (day before conditioning)**	**Day 0[6]**	**Day +4**	**Day +7**	**Day +14**	**Weekly through Day +100**	**Day +30 +/-7 days**	**Day +60 +/-7 days**	**Day +100 +/-14 days**	**Day +180 +/-14 days**	**Day +270 +/-14 days**	**Day +365 +/-14 days**
Demographics	x												
Conditioning Regimen	X												
GVHD Medications[11]			x		x	x		x	x	x	x		x
Liver function tests[1, 3]	x	x	x		x	x	x			x			
Routine blood tests[4]	x	x	x		x	x	x			x			
ECG	x	x[5]											
Qualitative Serum HcG pregnancy test for FCBP	x												
Fasting plasma glucose or HbA1c	x					x							
Thyroid function tests: TSH/free T4, GH/IGF-1	x					x							
Gallbladder ultrasound		X[8]		x[7]									
Citrulline assay [12.3.1]		x	x		x	x							
Calprotectin assay [12.3.2][9]		x	x		x	x							
Video Capsule Endoscopy [12.2][2]				x									
Toxicity Assessments		x	x		x	X[12]							

Acute GVHD Assessments			x		x	x	x			x	x		x
Chronic GVHD assessments										x	x		x
Survival Analysis										x	x		x

[1]ALT, aspartate aminotransferase, alkaline phosphatase, and total bilirubin, should be done weekly, or more frequently, if any value exceeds 5 times the baseline value in case of abnormal baselines or 5 times the ULN in case of normal baselines.
[2] For subset of 10 subjects; only performed at Duke site. To be performed between transplant Day +4 and +6.
[3] LFTs will occur at baseline and weekly starting Day 0 through day +100.
[4] CBC, Chemistry profile, PT/PTT, INR. At baseline/start of study drug visit, labs are to be performed prior to first dose of pasireotide.
[5] ECG will be done at 1, 1.5, and 2 hrs after first infusion of Pasireotide.
[6] Day 0 = the day of transplant.
[7] Gallbladder ultrasound to be completed between Transplant Day +4 and +6
[8] To be performed within 30 days prior to start of pasireotide.
[9] Baseline calprotectin sample to be collected within 72 hours prior to start of pasireotide. All other calprotectin samples may be collected +/- 1 day from expected assessment day.
[10] All screening assessments should be completed within 30 days prior to the start of pasireotide.
[11] Medications used for GVHD prophylaxis and all information regarding any GVHD treatment received during each study period will be recorded at each time point.
[12] Toxicity assessments to be completed at baseline, daily while on pasireotide, and then weekly through Transplant Day +35. Maximum grade of each toxicity to be recorded at Baseline, Days 0, +7, +14, +21, +28, and +35.

APPENDIX B: CAPSULE ENDOSCOPY PREPARATION INSTRUCTIONS

Your doctor has ordered a Video Capsule Endoscopy to evaluate your small intestines for conditions such as ulcers and bleeding. This procedure involves swallowing a capsule the size of a large vitamin pill. The capsule will pass through your small intestines taking pictures. The pictures will be transmitted to a small computer which you will wear on a belt around your waist. The test lasts 6 to 8 hours, during which time you may leave and go about your daily activities. At the end of that time, you will be asked to return the recording computer to the Gastroenterology (GI) Clinic, where the images will be downloaded for analysis. The video capsule is disposable and will usually pass naturally in your bowel movement within 24-48 hours. You will be asked to sign a consent form prior to the procedure because of the rare possibility that the capsule may not pass.

Please read and follow the directions below prior to your procedure. There will be a change to diabetic medications the DAY OF the procedure. If you are diabetic, call the GI Resource Nurse at 919-684-6437 to assist you with this. Call the GI Scheduling Hub at 919-684-6437 if you need to reschedule your appointment or for any other questions.

Three (3) Days Before the Procedure : stop taking iron supplements

One (1) Day Before The Procedure:
Stop taking antacids (Tums, Rolaids), Pepto-Bismol, or Carafate, which coats the camera lens.
AM : You may eat a regular breakfast.
11am: After breakfast, CONSUME ONLY CLEAR LIQUIDS for the remainder of the day and evening. No more solid food. Avoid anything that is colored red or purple. See below for list of clear liquids.

7pm: TAKE ONE DOSE (17 grams) OF MIRALAX over-the-counter laxative, mixed in water. (**Note: It is NOT necessary to move your bowels after this dose!)

12 Midnight : DO NOT EAT OR DRINK ANYTHING ELSE . If you have any medications due, you may take them with sips of water.

Day of the Procedure:
6am : You may take your morning medications with small sips of water. Do not take anything further by mouth.

o Diabetics: check blood sugar. Alter diabetic medications as discussed with GI Resource Nurse

Wear loose, comfortable 2-piece clothing. Avoid belts, dresses, pantyhose, or anything else restrictive around your waist. Men are no longer required to shave the chest or abdomen.

8 - 9am : Arrive at assigned (GI) Clinic. Location and time will be specified on your appointment letter. After meeting with the nurse and doctor, the recording computer will be attached to you with a belt and suspenders. You will then swallow the capsule with a glass of water.

Once the capsule is swallowed, specific instructions on diet and care of recording equipment will be given to you. A general guideline to the diet is as follows:
0-2 hours after swallowing capsule: Do not eat or drink.
2 hours after swallowing capsule: Clear liquids and medications.
4 hours after swallowing capsule: Light snacks.
6 hours after swallowing capsule: Regular diet.

The Capsule Endoscopy will last 6 to 8 hours. You will be allowed to leave, drive, and go about your daily activities (with some exceptions).

4 - 5pm : Return the equipment to the GI Clinic. Resume regular diet and activities.

Clear Liquid Diet List : (avoid anything that is colored Red or Purple)
Beverages: Soft Drinks ± orange, ginger ale, cola, Sprite, 7-Up, Gatorade G/G2, Kool-Aid, Strained fruit juices without pulp ± apple, white grape, orange, lemonade, Water, tea, coffee (no milk or non-dairy creamer)

Soups: Low-sodium chicken or beef bouillon/broth (broth may be strained off regular soup)

Desserts: Jell-O (lemon, lime, or orange; no fruit or toppings), Popsicles (no sherbet or fruit bars)

APPENDIX C: Guidance for hyperglycemia management

Hyperglycemia is known to be associated with the treatment with somatostatin analogues (SSA). Clinical studies of pasireotide in healthy volunteers and in patients with Cushing's disease, acromegaly or carcinoid syndrome have reported transient, asymptomatic increases in fasting and postprandial glucose levels.

Novartis has conducted 2 clinical studies ([SOM230B2216] and [SOM230B2124]) to further understand the mechanism of pasireotide-induced hyperglycemia and to evaluate the clinical utility of anti-diabetes agents in the management of pasireotide-induced hyperglycemia. Preliminary results suggest that pasireotide induces insulin suppression, particularly in the postprandial period, as being the key mechanistic driver of hyperglycemia.

Based on the mechanisms of pasireotide-induced hyperglycemia and findings from the [SOM230B2124] study, appropriate management for the pasireotide-induced hyperglycemia includes the use of oral anti-diabetic agents for mild to moderate hyperglycemia, such as incretin enhancers (e.g. GLP-1analogues or DPP4 inhibitors or insulin secretagogues). Metformin is not recommended. Insulin should be used for moderate to severe hyperglycemia.

Taking the above into account, hyperglycemia should be monitored and managed according to institutional practice.

APPENDIX D: QT Prolonging Medications

The following drugs are generally recognized to have an association with QT prolongation. This list is not conserved to be all inclusive.

Drugs that are generally accepted by the QTdrug.org Advisory Board of the Arizona CERT to have a risk of causing torsade de pointes are listed below

Generic Name	Brand Name	Class
Bepridil	Vascor®	Anti-anginal / heart pain
Amiodarone	Cordarone®	Anti-arrhythmic / abnormal heart rhythm
Amiodarone	Pacerone®	Anti-arrhythmic / abnormal heart rhythm
Disopyramide	Norpace®	Anti-arrhythmic / abnormal heart rhythm
Dofetilide	Tikosyn®	Anti-arrhythmic / abnormal heart rhythm
Ibutilide	Corvert®	Anti-arrhythmic / abnormal heart rhythm
Procainamide	Pronestyl®	Anti-arrhythmic / abnormal heart rhythm
Procainamide	Procan®	Anti-arrhythmic / abnormal heart rhythm
Quinidine	Cardioquin®	Anti-arrhythmic / abnormal heart rhythm
Quinidine	Quinaglute®	Anti-arrhythmic / abnormal heart rhythm
Sotalol	Betapace®	Anti-arrhythmic / abnormal heart rhythm
Clarithromycin	Biaxin®	Antibiotic / bacterial infection
Sparfloxacin	Zagam®	Antibiotic / bacterial infection
Erythromycin	Erythrocin®	Antibiotic;GI stimulant / bacterial infection; increase GI motility
Erythromycin	E.E.S.®	Antibiotic;GI stimulant / bacterial infection; increase GI motility
Arsenic trioxide	Trisenox®	Anti-cancer / Leukemia
Astemizole	Hismanal®	Antihistamine / Allergic rhinitis
Terfenadine	Seldane®	Antihistamine / Allergic rhinitis
Pentamidine	Pentam®	Anti-infective / pneumocystis pneumonia
Pentamidine	NebuPent®	Anti-infective / pneumocystis pneumonia
Probucol	Lorelco®	Antilipemic / Hypercholesterolemia
Chloroquine	Aralen®	Anti-malarial / malaria infection
Halofantrine	Halfan®	Anti-malarial / malaria infection
Domperidone	Motilium®	Anti-nausea / nausea
Mesoridazine	Serentil®	Anti-psychotic / schizophrenia
Thioridazine	Mellaril®	Anti-psychotic / schizophrenia

Haloperidol	Haldol®	Anti-psychotic / schizophrenia, agitation
Pimozide	Orap®	Anti-psychotic / Tourette's tics
Chlorpromazine	Thorazine®	Anti-psychotic/ Anti-emetic / schizophrenia/ nausea
Cisapride	Propulsid®	GI stimulant / heartburn
Levomethadyl	Orlaam®	Opiate agonist / pain control, narcotic dependence
Methadone	Dolophine®	Opiate agonist / pain control, narcotic dependence
Methadone	Methadose®	Opiate agonist / pain control, narcotic dependence
Droperidol	Inapsine®	Sedative;Anti-nausea / anesthesia adjunct, nausea

APPENDIX E: Study Drugs and Example Preparatory Regimens

The following is only an example of transplant preparatory regimen that can be used on this trial, however, any regimen that meets the definition of myeloablative as defined in this protocol is acceptable (TBI ≥ 1200 cGy, or Busulfan ≥ 12.8mg/kg). Similarly, GVHD prophylaxis (e.g. cyclosporine) may be as per institutional standard.

E1. Total Body Irradiation
Source: Total body irradiation will be delivered in a fractionated fashion over four days in a total of 8 fractions at 150 cGy per fraction to a total of 12 Gy using a Varian Linear Accelerator.

Toxicity: Acute - myelosuppression, complete ablation is expected in the setting of bone marrow transplantation. Severe nausea and vomiting, anorexia, diarrhea, alopecia, fever and mucositis, are common. Pulmonary fibrosis has also been reported. LFTs elevation can also be observed.

E2. Busulfan (Myerlan) NSC #750
Source and Pharmacology: Alkylating agent utilized intravenously. The drug is metabolized to several metabolites that are excreted into the urine. Approximately 25% to 35% of busulfan is eliminated via the kidneys as methanesulfonic acid. Busulfan has an elimination half-life around 2.5 hours.

Formulation: IV formulation.

Storage and Stability: Check manufacturer's expiration date. Store at room temperature.

Supplier: Commercially available from Otsuka.

Toxicity: Acute DLT - myelosuppression, primarily leukopenia, with a nadir of 8-14 days; complete ablation is expected in the setting of bone marrow transplantation. Nausea and vomiting, anorexia, diarrhea, alopecia, fever, mucositis, and hyperpigmentation are common. Liver dysfunction (increased LFTs) and renal or bladder dysfunction (increased BUN, creatinine, necrosis) may be seen. Pulmonary fibrosis has also been reported. Peripheral neuropathies and paresthesias have been occasionally observed. Seizures may occur in patients receiving busulfan therapy; all patients in this study should be prophylaxed with phenytoin (diphenylhydantoin).

Route of Administration: IV preparation

E3. Cyclophosphamide (Cytoxan, CTX) NSC #26271
Source and Pharmacology: An alkylating agent, related to nitrogen mustard, which is biochemically inert until it is metabolized to its active components by the liver phosphamidases. It is non-phase-specific. The drug is excreted exclusively by the kidney after parenteral administration. The plasma half-life ranges from 4-6.5 hours.

Formulation and Stability: A freeze-dried powder available in 100 mg and 500 mg vials with diluent which contains 0.9% benzyl alcohol in water. The unreconstituted form of the drug is stable at room temperature for at least 2 years. Reconstitute with 5 ml of diluent to 100 mg vial and 10 ml to the 500 mg vial. Also available as 1 g and 2 g vials without diluent. Reconstitute with 10 ml and 20 ml sterile water or bacteriostatic water. The reconstituted solution is stable at room temperature for 48 hours. Solutions that have a slight haze should be discarded.

Supplier: Commercially available.

Toxicity: Acute DLT - myelosuppression, primarily leukopenia, with a nadir of 8-14 days; complete ablation is expected in the setting of bone marrow transplantation. Hemorrhagic cystitis is a potential complication, especially of high-dose therapy. All patients receiving high-dose CTX should have a three-way foley catheter with continuous irrigation in place for the duration of CTX therapy and 24 additional hours after the last dose. If the patient is too small for a three-way foley catheter, a regular foley catheter should be used and continuous bladder irrigation can be omitted. Other adverse reactions include anorexia, nausea and vomiting, alopecia, fluid retention, hyponatremia, inappropriate ADH, immunosuppression, and gonadal suppression with associated sterility. Pulmonary fibrosis is rare. Very high doses over a short period of time may be associated with myocardial necrosis, transiently blurred vision, and cardiac toxicity with arrhythmias. Consideration to holding a dose of CTX should be given for patients who develop otherwise unexplained tachycardia or a drop in QRS voltage of >25% during cyclophosphamide administration.

Route of Administration: The total daily dose will be given as a 1-hour IV infusion in D5NS. Patients should receive additional hydration with 3000 ml/m^2/day of appropriate maintenance IV fluids.

E4. Cyclosporine (Sandimmune)

Source and Pharmacology: A cyclic polypeptide immunosuppressant agent consisting of 11 amino acids. The exact mechanism of action of cyclosporine is not known, but experimental evidence suggests that its effectiveness is due to specific and reversible inhibition of immuno-competent lymphocytes in the G_0 or G_1-phase of the cell cycle. T-lymphocytes are preferentially inhibited. The T-helper cell is the main target, although the T-suppressor cell may also be suppressed. Absorption of cyclosporine from the gastrointestinal tract is incomplete and variable. Peak concentrations (C_{max}) in blood and plasma are achieved at about 3.5 hours. The disposition of cyclosporine from blood is biphasic with a terminal half-life of approximately 19 hours (range: 10-27 hours). Elimination is primarily biliary with only 6% of the dose excreted in the urine.

Formulation and Stability: Oral solution in 50 ml bottles (100 mg/ml), soft gelatin capsules (25 mg and 100 mg/capsule), and IV 5 ml ampoules (50 mg/ml). Store at room temperature. The oral cyclosporine is in an olive oil solution which should be utilized within two months after the bottle is opened. It may be mixed in whole milk,

chocolate milk, or fruit juice to mask the taste and should be taken immediately after mixing. The IV solution should be mixed in D5W to a concentration of 2.5-10 mg/ml and should be used within 24 hours after mixing.

Supplier: Commercially available from Sandoz Pharmaceuticals.

Toxicity: Renal dysfunction; increased blood pressure; hirsutism; tremors or seizures; paresthesias; liver toxicity; hyperkalemia or hypokalemia; low serum magnesium; gastrointestinal complaints including anorexia, nausea, and ileus; pancytopenia; gingival hyperplasia; skin pigmentation changes; mental depression; capillary leak syndrome; lymphoproliferative disorders (malignant lymphoma)

Route of Administration: Continuous IV infusion, or PO at treating physician's discretion.

Precautions: Nephrotoxicity is felt to be related to whole blood drug level and for that reason whole blood samples will be obtained at least 3 times weekly (Monday, Wednesday, and Friday) throughout the first 21 days post-transplant (or until switched to PO therapy) for the determination of cyclosporine levels. Blood samples must be obtained from a line which is not in use for constant infusion of cyclosporine. The whole blood cyclosporine level should not be <100 ng/ml and/or >300 ng/ml. Another guide to cyclosporine dosing can be assessed by the serum creatinine level. The cyclosporine dose should be reduced by 25% if the serum creatinine is between 1.5-1.7 mg%. The dose should be further reduced 50% if the creatinine is 1.8-2.0 mg% and the drug held if the serum creatinine is >2.0 mg%. Furthermore the drug dose may need to be modified in patients with liver disease, significant grand mal tremors, significant pancytopenia, and poorly-controlled hypertension. Cyclosporine therapy may be restarted when the whole blood drug level is <300 ng/ml and the serum creatinine returns to treatable levels.

E5. Methotrexate (MTX)

MTX is an analog of aminopterin, the folic acid antagonist introduced in 1948 for the treatment of acute leukemia. As one of the antimetabolites, it functions as a decoy substrate for critical biochemical reactions leading to cell death. The amino group substitution found in MTX results in a less potent antifolate but a drug with more predictable toxicity profile and equivalent clinical results. The precise mechanism by which MTX prevents GVHD is not understood; however, it is likely related to MTX's ability to inhibit cellular growth and division. Therefore, antigen activated T cells that are rapidly proliferating would be particularly sensitive to this antimetabolite. MTX can induce tolerance following marrow transplantation. In canine transplantation, MTX given after the graft was effective in controlling GVHD and in inducing tolerance when donor and recipient were matched for the dog leukocyte antigen system.

Pharmacology

MTX was one of the first chemotherapy agents introduced for the therapy of malignancies. Studies have detailed its pharmacokinetics and mechanisms of action at the intracellular level. How these mechanisms translate to its *in vivo* effects and how it

functions in the prevention of GVHD, facilitation of engraftment, and induction of tolerance are not well understood. At the cellular level, MTX exerts its cytotoxic effect by inhibiting dihydrofolate reductase (DHFR). DHFR is the intracellular enzyme responsible for converting folic acid to reduced folate cofactors. The reduced state of the folates, namely the tetrahydrofolates are responsible for the transport of single carbon groups that are required for purine and thymidylate synthesis. Once the single carbon group is delivered, the oxidized folates must be converted back to tetrahydrofolates by DHFR. MTX binds to DHFR, thereby preventing its ability to reduce the oxidized folates to tetrahydrofolate and blocking further purine or thymidylate synthesis.

Administration

Following the administration of MTX approximately 50% is bound to plasma proteins, especially albumin, with the highest tissue-plasma equilibrium reported in the kidney, liver, GI tract and muscle. The GI tract is an important site of distribution and metabolism of MTX, perhaps accounting for one of the primary sites of toxicity. One concern in using MTX is its clearance in those patients who have pleural or ascitic fluid since these reservoir spaces can have a substantial impact on the disposition of MTX. These spaces can be a reservoir from which MTX can be continuously released, leading to severe toxicity. At the low IV doses, MTX clearance correlates with glomerular filtration and renal excretion of unmetabolized drug is the major route for elimination.

Polyglutamate formation is also important to MTX metabolism. The formation of MTX polyglutamates intracellularly may account for its retention within the cell and its cytotoxic effects. The enzyme folyl polyglutamate synthetase adds glutamate residues to folates or antifolates such as MTX. These polyglutamate moieties are then retained inside the cell leading to prolonged antifolate activity. The polyglutamate derivatives of MTX appear to be as toxic to DHFR as the native compound. If the retained MTX polyglutamates are produced differentially in lymphocytes compared to other normal cells, the effective concentrations of the drug would differ. For example, these differences in intracellular concentrations may account for selective toxicities and may selectively inhibit certain subpopulations of cells.

Toxicity

The most common adverse effects of MTX when used for GVHD prophylaxis are hematopoietic, renal, hepatic, and GI mucosal toxicity. Patients may have an elevation in creatinine and elevation of bilirubin levels following the administration of MTX. As many of these are also the side-effects of CSP or tacrolimus, dose attenuation of MTX and CSP or tacrolimus may be necessary. Full target doses may not be achievable in patients with grade III-IV mucositis resulting from the preparatory regimen because of airway obstruction and severe oropharyngeal bleeding.

Clinical use

The use of MTX following allogeneic BMT has been exclusively with the IV formulation. The drug doses administered have been relatively small given the inability to dose escalate MTX significantly. Initial studies in Seattle tested MTX as a single agent on a

weekly basis up to day +100 but, because of the toxicity of weekly administration of MTX, the dose was changed to four doses and combined with CSP. MTX, when used with CSP or tacrolimus, generally is injected IV on days +1, +3, +6 and +11 following BMT.

E6. Example Schedule

	TBI/Cytoxan	Busulfan/Cytoxan
Day	Treatment	Treatment
-8	Hickman at 0700<br>TBI X 1 (150 cGy)	Admit 9200<br>Start Busulfan 12 am<br>0.8 mg/kg IV Q6hrs X 16 doses
-7	TBI X 2 (150 cGy each)	Busulfan Q6hrs
-6	TBI X 2 (150 cGy each)	Busulfan Q6hrs
-5	TBI X 2 (150 cGy each)	Busulfan Q6hrs last dose 6 pm
-4	TBI X 2 (150 cGy each)	Cyclophosphamide 60/mg/kg
-3	Cytoxan 60 mg/kg	Cyclophosphamide 60 mg/kg
-2	Cytotoxan 60 mg/kg	Rest
-1	Rest	Rest
0	Stem Cell Infusion	Stem cells infused

APPENDIX F: Acute GVHD Scoring

Acute GVHD Scoring (BMT CTN, 2013)

Stage	**Skin**	**GI**	**Liver**
**1**	< 25% rash	Diarrhea > 500ml/d or persistent nausea	Bilirubin 2-3mg/dl
**2**	25-50%	> 1000 ml/d	Bilirubin 3-6 mg/dl
**3**	> 50%	> 1500 ml/d	Bilirubin 6-15 mg/dl
**4**	Generalized erythroderma with bullae	Large volume diarrhea and severe abdominal pain ± ileus	Bilirubin > 15 mg/dl

**Grade**	**Skin**	**GI**	**Liver**
I	Stage 1-2	0	0
**II**	Stage 3 or	Stage 1 or	Stage 1
**III**	---	Stage 2-4	Stage 2-3
**IV**	Stage 4	---	Stage 4

APPENDIX G: Chronic GVHD Scoring

Chronic GVHD: 2014 NIH Consensus Criteria (Jagasia et. al., 2014)

Organ Scoring

	SCORE 0	SCORE 1	SCORE 2	SCORE 3
**PERFORMANCE SCORE:** _____<br>☐ **KPS**<br>☐ **ECOG**<br>☐ **LPS**	☐ Asymptomatic and fully active (ECOG 0; KPS or LPS 100%)	☐ Symptomatic, fully ambulatory, restricted only in physically strenuous activity (ECOG 1, KPS or LPS 80-90%)	☐ Symptomatic, ambulatory, capable of self-care, >50% of waking hours out of bed (ECOG 2, KPS or LPS 60-70%)	☐ Symptomatic, limited self-care, >50% of waking hours in bed (ECOG 3-4, KPS or LPS <60%)
**SKIN†**<br>**SCORE % BSA:** _____				
GVHD features to be scored by BSA:<br>**Check all that applies:**<br>☐ Maculopapular rash/erythema<br>☐ Lichen planus-like features<br>☐ Sclerotic features<br>☐ Papulosquamous lesions or ichthyosis<br>☐ Keratosis pilaris-like GVHD	☐ No BSA involved	☐ 1-18% BSA	☐ 19-50% BSA	☐ >50% BSA
**SKIN FEATURES SCORE:** _____		☐ No sclerotic features	☐ Superficial sclerotic features "not hidebound" (able to pinch)	**Check all that applies:**<br>☐ Deep sclerotic features<br>☐ "Hidebound" (unable to pinch)<br>☐ Impaired mobility<br>☐ Ulceration
Other skin GVHD features (NOT scored by BSA)<br>**Check all that applies:**<br>☐ Hyperpigmentation<br>☐ Hypopigmentation<br>☐ Poikiloderma<br>☐ Severe or generalized pruritus<br>☐ Hair involvement<br>☐ Nail involvement				
☐ *Abnormality present but explained entirely by non-GVHD documented cause (specify):*______________________________				
† Skin scoring should use both percentage of BSA involved by disease signs and the cutaneous features scales. When a discrepancy exists between the percentage of total body surface (BSA) score and the skin feature score, OR if superficial sclerotic features are present (Score 2), but there is impaired mobility or ulceration (Score 3), the higher level should be used for the final skin scoring.				

	SCORE 0	SCORE 1	SCORE 2	SCORE 3
**MOUTH**<br>*Lichen planus-like features present:*<br>☐ Yes<br>☐ No	☐ No symptoms	☐ Mild symptoms **with** disease signs but not limiting oral intake significantly	☐ Moderate symptoms with disease signs **with** partial limitation of oral intake	☐ Severe symptoms with disease signs on examination **with** major limitation of oral intake
☐ *Abnormality present but explained entirely by non-GVHD documented cause (specify):*______________________________				

	SCORE 0	SCORE 1	SCORE 2	SCORE 3
**EYES**<br>*Keratoconjunctivitis sicca (KCS) confirmed by Ophthalmologist:*<br>☐ Yes<br>☐ No	☐ No symptoms	☐ Mild dry eye symptoms not affecting ADL (requirement of lubricant eye drops < 3 x per day)	☐ Moderate dry eye symptoms partially affecting ADL (requiring lubricant eye drops > 3 x per day or punctal plugs), **WITHOUT** new vision impairment due to KCS	☐ Severe dry eye symptoms significantly affecting ADL (special eyeware to relieve pain) **OR** unable to work because of ocular symptoms **OR** loss of vision due to KCS
☐ *Abnormality present but explained entirely by non-GVHD documented cause (specify):*______________________________				
	**SCORE 0**	**SCORE 1**	**SCORE 2**	**SCORE 3**
**GI Tract**<br>***Check all that applies:***<br>☐ Esophageal web/ proximal stricture or ring<br>☐ Dysphagia<br>☐ Anorexia<br>☐ Nausea<br>☐ Vomiting<br>☐ Diarrhea<br>☐ Weight loss*<br>☐ Failure to thrive	☐ No symptoms	☐ Symptoms without significant weight loss* (<5%)<br><br>*within last 3 months	☐ Symptoms associated with mild to moderate weight loss* (5-15%)<br><br>*within last 3 months	☐ Symptoms associated with significant weight loss* >15%, requires nutritional supplement for most calorie needs or esophageal dilation<br><br>*within last 3 months
☐ *Abnormality present but explained entirely by non-GVHD documented cause (specify):*______________________________				
	**SCORE 0**	**SCORE 1**	**SCORE 2**	**SCORE 3**
**LIVER**	☐ Normal total bilirubin and ALT < 2 x NUL	☐ Normal total bilirubin and ALT >2 x NUL	☐ Elevated total Bilirubin but <3 mg/dL NUL	☐ Elevated total bilirubin > 3 x NUL
☐ *Abnormality present but explained entirely by non-GVHD documented cause (specify):*______________________________				
	**SCORE 0**	**SCORE 1**	**SCORE 2**	**SCORE 3**
**LUNGS****<br>**Symptoms score**:___	☐ No symptoms	☐ Mild symptoms (shortness of breath after climbing one flight of steps)	☐ Moderate symptoms (shortness of breath after walking on flat ground)	☐ Severe symptoms (shortness of breath at rest; requiring 0_2)
**Lung obstructive function score: ____**<br>FEV1: ____	☐ FEV1≥80%	☐ FEV1 60-79	☐ FEV1 40-59%	☐ FEV1 <39%
Pulmonary function tests: ____				

☐ Not performed
☐ *Abnormality present but explained entirely by non-GVHD documented cause (specify):*______________________________
**Lung scoring should be performed using both the symptoms and FEV1 scores whenever possible. FEV1 should be used in the final lung scoring where there is discrepancy between symptoms and FEV1 scores.

	SCORE 0	SCORE 1	SCORE 2	SCORE 3
**JOINTS AND FASCIA**<br>P-ROM score<br>Shoulder (1-7):___<br>Elbow (1-7): ____<br>Wrist/finger (1-7):___<br>Ankle (1-4):___<br><br>***P-ROM Chart***	☐ No symptoms	☐ Mild tightness of arms or legs, normal or mild decreased range of motion (ROM) **AND** not affecting ADL	☐ Tightness of arms or legs **OR** joint contractures, erythema thought due to fasciitis, moderate decrease ROM **AND** mild to moderate limitation of ADL	☐ Contractures **WITH** significant decrease of ROM ***AND*** significant limitation of ADL (unable to tie shoes, button shirts, dress self etc.)

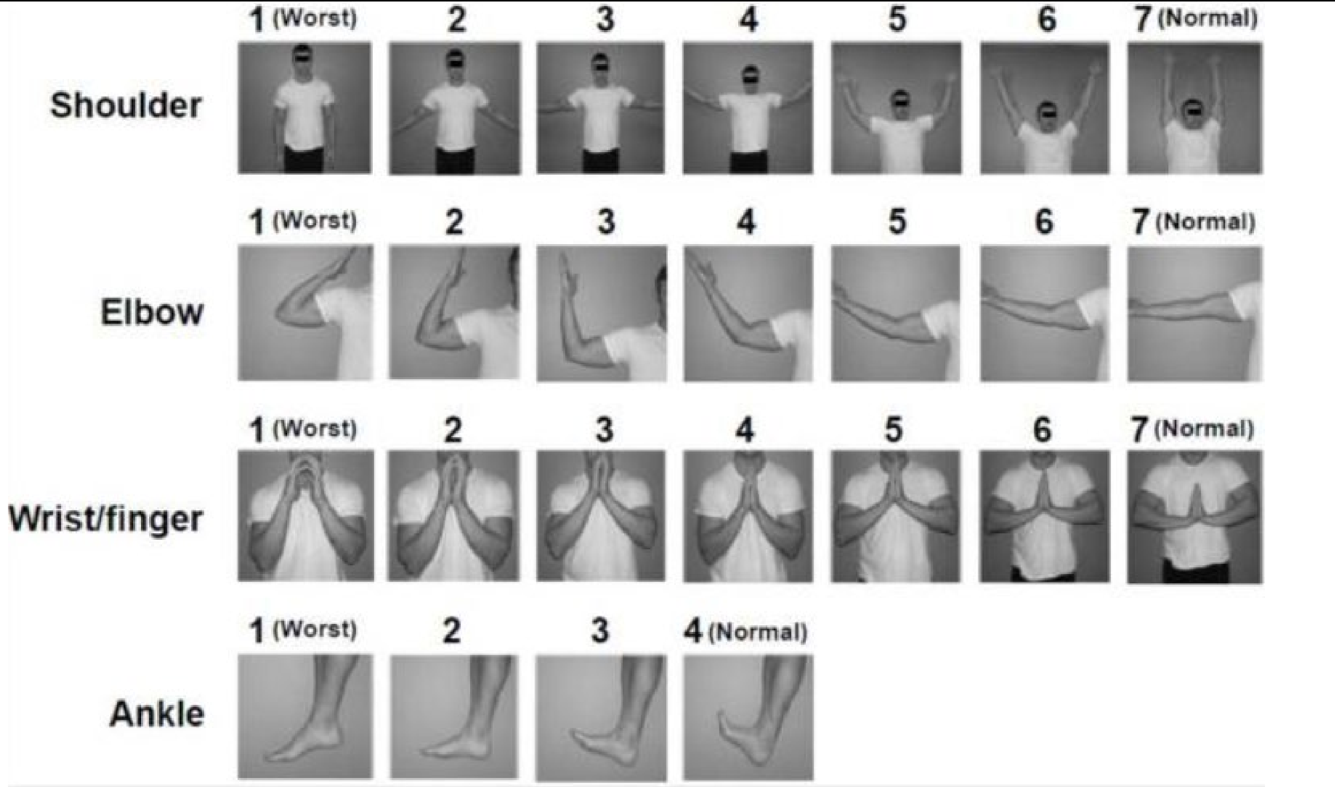

☐ *Abnormality present but explained entirely by non-GVHD documented cause (specify):*_________________________

	SCORE 0	SCORE 1	SCORE 2	SCORE 3
**GENITAL TRACT**<br>(*See Supplemental table‡*)<br>**Check all that applies**<br>☐ Not examined<br>*Currently sexually active*<br>☐ Yes<br>☐ No	☐ No signs	☐ Mild signs‡ and females with or without discomfort on exam	☐ Moderate signs‡ and may have signs* of discomfort on exam	☐ Severe signs‡ with or without symptoms

Check all signs that apply:

☐ Lichen planus-like features
☐ Lichen sclerosis-like features
☐ Vaginal scarring (female)
☐ Clitoral/labial agglutination (female)
☐ Labial resorption (female)
☐ Erosions
☐ Fissures
☐ Ulcers
☐ Phimosis (male)
☐ Urethral meatus scarring/ stenosis (male)

Female genitalia: Severity of signs:
1) **Mild** (any of the following); erythema on vulvar mucosal surfaces, vulvar lichen-planus or vulvar lichen-sclerosis
2) **Moderate** (any of the following); erosive inflammatory changes of the vulvar mucosa, fissures in vulvar folds
3) **Severe** (any of the following); labial fusion, clitoral hood agglutination, fibrinous vaginal adhesions, circumferential
fibrous vaginal banding, vaginal shortening, synechia, dense sclerotic changes, and complete vaginal stenosis
Male genitalia: Diagnostic features include lichen planus-like or lichen sclerosis-like features and phymosis or urethral scarring or stenosis. Severity of signs: **Mild** – lichen planus-like feature; **Moderate** – lichen sclerosis-like feature or moderate erythema; **Severe** – phimosis or urethral/meatal scarring

☐ *Abnormality present but NOT thought to represent GVHD (specify cause):*___________________________________
☐ *Abnormality thought to represent GVHD PLUS other causes (specify cause):*__________________________________

Other indicators, clinical features or complications related to chronic GVHD (check all that apply and assign a score to its severity (0-3) based on its functional impact where applicable none – 0,mild -1, moderate -2, severe – 3)

☐ Ascites (serositis)___
☐ Pericardial Effusion___
☐ Pleural Effusion(s)___
☐ Nephrotic syndrome___
☐ Myasthenia Gravis___
☐ Peripheral Neuropathy___
☐ Polymyositis___
☐ Weight loss* without GI symptoms
☐ Eosinophilia > 500µl___
☐ Platelets <100,000/µl ___
☐ Others (specify):_________________

**Overall GVHD Severity** *(Opinion of the evaluator)*	☐ No GVHD	☐ Mild	☐ Moderate	☐ Severe

**Mild chronic GVHD**	**Moderate chronic GVHD**	**Severe chronic GVHD**
1or 2 organs involved (not lung) *plus*<br>Score in involved organs 1 *plus*<br>Lung score 0	3 or more organs involved *plus*<br>Score of 1 in each organ<br>OR<br>At least 1 organ (not lung) with a score of 2<br>OR<br>Lung score 1	At least 1 organ with a score of 3<br>OR<br>Lung score of 2 or 3

Appendix H: SAE Report Coversheets

NOVARTIS

Interventional Clinical Trial SAE Fax Cover Sheet

To: Local Novartis Drug Safety and Epidemiology Safety Desk **1-877-778-9739**

Investigator contact details:
Fax number : ___________________________
Phone number : _________________________

Study Name	
Centre Number	
Patient Number	

Relationship between study treatment and event(s) is:

Suspected/Unknown

Investigator Signature	

This document contains important safety information.

If fax is received in error, please forward to 1 877 778 9739

NOVARTIS

Interventional Clinical Trial SAE Fax Cover Sheet

To: Local Novartis Drug Safety and Epidemiology Safety Desk **1-877-778-9739**

Investigator contact details:
Fax number : ______________________________
Phone number : ____________________________

Study Name	
Centre Number	
Patient Number	

Relationship between study treatment and event(s) is:

Not Suspected

Investigator Signature	

This document contains important safety information.

If fax is received in error, please forward to 1 877 778 9739
