# Supplementary material for: A phase 2 trial of the somatostatin analog pasireotide to prevent GI toxicity and acute GVHD in allogeneic hematopoietic stem cell transplant
Source: PLoS One. 2021 Jun 25;16(6):e0252995. doi: 10.1371/journal.pone.0252995 (PMC8232534; doi:10.1371/journal.pone.0252995)
Supplement: S5 File — (DOCX) [file pone.0252995.s005.docx]

*Blood-based Biomarker Analysis*

A multiplex protein array system from Meso Scale Diagnostic (MSD) was used to analyze biomarkers of immune/inflammatory response. We used the V-PLEX Human Biomarker 54-Plex Kit (Cat#K15248D-1, MSD, Rockville, MD) which evaluates CRP, Eotaxin, Eotaxin-3, FGF (basic), GM-CSF, ICAM-1, IFN-γ, IL-10, IL-12/IL-23p40, IL-12p70, IL-13, IL-15, IL-16, IL-17A, IL-17A/F, IL-17B, IL-17C, IL-17D, IL-1RA, IL-1α, IL-1β, IL-2, IL-21, IL-22, IL-23, IL-27, IL-3, IL-31, IL-4, IL-5, IL-6, IL-7, IL-8, IL-8 (HA), IL-9, IP-10, MCP-1, MCP-4, MDC, MIP-1α, MIP-1β, MIP-3α, PlGF, SAA, TARC, Tie-2, TNF-α, TNF-β, TSLP, VCAM-1, VEGF-A, VEGF-C, VEGF-D, and VEGFR-1/Flt-1.

In addition, plasma samples were used for quantitative evaluation of the Duke Pepper Panel. The Pepper Panel includes the following markers: Adiponectin, IL-2, IL-6, TNFa, TNFRI*, TNFRII*, D-Dimer*, G-CSF, regulated on activation, normal T cell expressed and secreted (RANTES), matrix metalloproteinase-3 (MMP-3), Paraoxonase, VCAM-1.(61) However, of the 12 Pepper Panel markers, 6 were included in the 54 plex panel, and 6 were analyzed separately (denoted by *). Other biomarkers of GVHD (e.g., ST2 and REG3A) and IL6Ra were also analyzed by ELISAs (MSD and R&D Systems Inc., Minneapolis, MN). IL1RL/ST2 (MesoScaleDiscovery Cat#F214H-3), MMP-3 (MesoScaleDiscovery Cat#F21ZF-3), RANTES (MesoScaleDiscovery Cat#F21ZN-3), TNFRI/TNFRII (MesoScaleDiscovery Cat#F210V-3/F21ZS-3): D-Dimer (Sekisui Diagnostics Cat#602), IL6Ra (R&D Systems Cat#DR600), REG3a (MBL Cat#5323/5310), Paraoxonase (Invitrogen Molecular Probes Cat#E33702). Blood samples from pre-HCT (baseline) and day 14 were included in the analysis.

*Blood and Stool Metabolomics*

For blood-based metabolomics assays we evaluated 65 metabolic biomarkers, including 3-Hydroxybutyrate, lactate, non-esterified fatty acids, triglycerides, glycerol, amino acids (N=15), and acylcarnitines (N=45). Measurements were made by flow-injection tandem mass spectrometry, using sample preparation methods described previously (62, 63). Measurements of analytes in EDTA-plasma were performed using a Beckman DxC 600 clinical analyzer. Reagents for 3-hydroxybutyrate and non-esterified fatty acids (NEFA) were from Wako (Mountain View, CA), and lactate and triglycerides from Beckman (Brea, CA). We additionally measured free glycerol using the initial absorbance in the triglycerides assay, a signal which is normally blanked in the test procedure preceding the addition of lipase. Amino acids and acylcarnitines were analyzed by flow injection electrospray ionization tandem mass spectrometry and quantified by isotope or pseudo-isotope dilution using methods described previously (62, 63).

Extracts from stool samples were prepared at 10 mg per mL, vortexed and homogenized on a shaker for 30 min before 10,000 g centrifugation to collect the supernatant. After further 2-fold dilution, stool samples were assayed for Beta-Defensin 2 using a kit from ALPCO ( Salem, NH, thru ß-Defensin 2 ELISA kit, cat# K 6500, Immundiagnostik AG, Stubenwald-Allee 8a, 64625 Bensheim, Germany). Calprotectin was assayed using a kit from Inova Diagnostic (San Diego, CA) after further 100-fold dilution.

*Stool Microbiome*

For microbiome analyses, stool samples were shipped to and sequenced at Memorial Sloan Kettering Cancer Center according to previously published methods (24, 64).

*Statistical analysis for Biomarker studies*

The 54-plex immune/inflammatory response biomarkers, Pepper Panel, and Metabalomics biomarkers were included in the blood-based analysis. OS time was defined as time from transplant to death/last follow-up. Blood samples from pre-HCT (baseline) and day 14 were used for purposes of comparison

Expression levels were log-transformed and analyzed as continuous measures. Pair-wise comparisons in the biomarkers of pre-treatment (baseline) and post-treatment (day 14) in each arm were done using the Wilcoxon signed-rank test (65). Association of each biomarker and OS was assessed using Cox proportional hazards models. Hazards ratios (HR) and Wald test p-values were reported. Associations of each biomarker and GVHD and TRM was assessed using logistic regression models. Odds ratios (OR) and score test p-values were reported. These models tested the association of baseline biomarker levels with the outcome, and were then repeated to test the association the ratio of change from baseline to day 14 instead. Multiple comparisons were addressed within a framework of control of False Discovery Rate (FDR) using the method by Storey (66-68).

All analyses were performed using the R Statistical Environment (69), and extension packages from CRAN(66, 69). The analyses were conducted with adherence to the principles of reproducible analysis using the knitr package for generation of dynamic reports (70).

*Statistical analysis for Microbiome studies:*

Microbiome samples were prepared and sequenced over several sequencing runs, which included samples from patients not included in this study. Quality control was conducted on the raw sequencing results using fastQC (71), and MultiQC (72) was used to compile the results by sequencing run. Forward and reverse read trimming lengths were based on the summarized read quality. Samples were then processed by sequencing run using DADA2 (73), following the developer-designed workflow (v1.12, https://benjjneb.github.io/dada2/tutorial.html), using the same trimming and filtering criteria across all runs. Taxonomy was assigned to the resulting amplicon sequence variants (ASVs) based on taxonomy and species reference data from the Ribosomal Database Project (RDP) (74) (trainset 16 preformatted reference fasta, https://zenodo.org/record/801828). Finally, samples were filtered to only those belonging to the study patient population and with a collection date within 28 days pre- or post-patient transplant date. The pre-transplant period was further divided into pre- or post-start of conditioning.

Diversity was calculated for each sample using the Shannon Index (75). To account for variability in diversity observed across samples, mean Shannon diversity was calculated for each patient for each time period. Baseline mean Shannon diversity was then defined for each patient as either pre-conditioning mean Shannon diversity or during-conditioning mean Shannon diversity, depending on which period had more samples available for that patient.

Mean post-transplant diversity was modeled using generalized linear models (GLMs) as a function of treatment (pasireotide, yes or no), with additional covariates to adjust for mean baseline diversity, whether baseline was measured before or during the conditioning period, and an indicator for the receipt of TBI during conditioning. An interaction term for use of pasireotide and receipt of TBI was then added to test for differences in the effect of pasireotide between conditioning regimens.

The model was then further expanded by the addition of a covariate indicating whether post-transplant diversity was measured after the onset of febrile neutropenia (FN), which served as a surrogate indicator for the receipt of broad-spectrum antibiotics. An alternative method of accounting for the effect of antibiotics was also tested, replacing the FN indicator with one based on receipt of antibiotics targeting anaerobes, (specifically Amoxicillin, Ampicillin, Amoxicillin/Clavulanate, Piperacillin/Tazobactam, Meropenem, Ertapenem, Metronidazole, Moxifloxacin, or Clindamycin).

In a parallel investigation, Relative abundance (RA) of bifidobacteria and clostridia (all clusters, and I, IV, XIVa, XV and XVI only) were calculated for each sample based on the total numbers of reads annotated at the genus level. As above, mean RA for each microbe was computed for each time period. The association of the use of pasireotide with mean post-transplant RA for each microbe was tested using GLMs. Similar to the above model, mean post-transplant RA was modeled as a function of treatment (pasireotide, yes or no), mean baseline RA, whether baseline was measured before or during conditioning, indicator of TBI, pasireotide-by-TBI interaction term, and an indicator of FN. Mean RA values were log-transformed for the model.

The p-values from these analyses were not adjusted to account for multiple testing. All inferential microbiome analyses were carried out using the R Statistical Environment(69) along with extension packages from the Comprehensive R Archive Network (CRAN; <https://cran.r-project.org/>), including tidyverse (76)and lubridate(77), and the Bioconductor project(78). The analyses were carried out with adherence to the principles of reproducible analysis using the knitr package (79) for generation of dynamic reports and Duke’s gitlab (<https://gitlab.oit.duke.edu/>) for source code management. The code for replicating the statistical analysis of the microbiome data will be accessible through a public source code repository (<https://gitlab.oit.duke.edu/dcibioinformatics/pubs/sung-microbiome-pastireotide>).

References:

61. Parker D, Sloane R, Pieper CF, Hall KS, Kraus VB, Kraus WE, et al. Age-Related Adverse Inflammatory and Metabolic Changes Begin Early in Adulthood. The journals of gerontology Series A, Biological sciences and medical sciences. 2019;74(3):283-9.

62. An J, Muoio DM, Shiota M, Fujimoto Y, Cline GW, Shulman GI, et al. Hepatic expression of malonyl-CoA decarboxylase reverses muscle, liver and whole-animal insulin resistance. Nat Med. 2004;10(3):268-74.

63. Ferrara CT, Wang P, Neto EC, Stevens RD, Bain JR, Wenner BR, et al. Genetic networks of liver metabolism revealed by integration of metabolic and transcriptional profiling. PLoS Genet. 2008;4(3):e1000034.

64. Peled JU, Devlin SM, Staffas A, Lumish M, Khanin R, Littmann ER, et al. Intestinal Microbiota and Relapse After Hematopoietic-Cell Transplantation. Journal of clinical oncology : official journal of the American Society of Clinical Oncology. 2017;35(15):1650-9.

65. Whitley E, Ball J. Statistics review 6: Nonparametric methods. Critical care (London, England). 2002;6(6):509-13.

66. Owzar K, Barry WT, Jung SH. Statistical Considerations for Analysis of Microarray Experiments. Cts-Clinical and Translational Science. 2011;4(6):466-77.

67. Storey JD. A direct approach to false discovery rates. J Roy Stat Soc B. 2002;64:479-98.

68. Storey JD. False Discovery Rate. In: Lovric M, editor. International Encyclopedia of Statistical Science. Berlin, Heidelberg: Springer Berlin Heidelberg; 2011. p. 504-8.

69. R Core Team (2020). R: A language and environment for statistical computing. R Foundation for Statistical Computing V, Austria. URL <https://www.R-project.org/>.

70. . Yihui X. Dynamic Documents with R and knitr. 2nd edition. Chapman and Hall/CRC 2015.

71. Andrews S. FastQC A Quality Control tool for High Throughput Sequence Data 2014. Available from: <https://www.bioinformatics.babraham.ac.uk/projects/fastqc>.

72. Ewels P, Magnusson M, Lundin S, Kaller M. MultiQC: summarize analysis results for multiple tools and samples in a single report. Bioinformatics. 2016;32(19):3047-8.

73. Callahan BJ, McMurdie PJ, Rosen MJ, Han AW, Johnson AJ, Holmes SP. DADA2: High-resolution sample inference from Illumina amplicon data. Nat Methods. 2016;13(7):581-3.

74. Cole JR, Wang Q, Fish JA, Chai B, McGarrell DM, Sun Y, et al. Ribosomal Database Project: data and tools for high throughput rRNA analysis. Nucleic Acids Res. 2014;42(Database issue):D633-42.

75. Shannon CWW. The Mathematical Theory of Communication. Urbana: University of Illinois Press; 1949.

76. al We. Welcome to the tidyverse. Journal of Open Source Software. 2019;4(43):1686.

77. Wickham GGaH. Dates and Times Made Easy with lubridate. Journal of Statistical Software. 2011;40(3):1-25.

78. Gentleman RC CV, Bates DM, Bolsted B, Dettling M, Dudoit S, et al. Biocundoctor open software development for computational biology and bioinformatics. Genome Biol. 2004;5(10):R80.

79. X Y. Dynamic Documents with R and knitr 2nd edition. Hall/CRC Ca, editor2015.
